# Injury primes mutation-bearing astrocytes for dedifferentiation in later life

**DOI:** 10.1016/j.cub.2023.02.013

**Published:** 2023-03-27

**Authors:** Holly Simpson Ragdale, Melanie Clements, Wenhao Tang, Elitza Deltcheva, Catia Andreassi, Alvina G. Lai, Wai Hoong Chang, Maria Pandrea, Ivan Andrew, Laurence Game, Imran Uddin, Michael Ellis, Tariq Enver, Antonella Riccio, Samuel Marguerat, Simona Parrinello

**Affiliations:** 1 Samantha Dickson Brain Cancer Unit, UCL Cancer Institute, University College London, London WC1E 6DD, UK; 2 MRC London Institute of Medical Sciences, Du Cane Road, London W12 0NN, UK; 3 Institute of Clinical Sciences, Faculty of Medicine, Imperial College London, Du Cane Road, London W12 0NN, UK; 4 UCL Cancer Institute, University College London, London WC1E 6DD, UK; 5 UCL Laboratory for Molecular Cell Biology, University College London, London WC1E 6BT, UK; 6 Institute of Health Informatics, University College London, London NW1 2DA, UK; 7 CRUK City of London Centre Single Cell Genomics Facility, UCL Cancer Institute, University College London, London WC1E 6DD, UK; 8 Genomics Translational Technology Platform, UCL Cancer Institute, University College London, London WC1E 6DD, UK

**Keywords:** astrocytes, dedifferentiation, brain injury, aging, p53, mTOR signaling, EGFR signaling, neural stem cells, neuroinflammation

## Abstract

Despite their latent neurogenic potential, most normal parenchymal astrocytes fail to dedifferentiate to neural stem cells in response to injury. In contrast, aberrant lineage plasticity is a hallmark of gliomas, and this suggests that tumor suppressors may constrain astrocyte dedifferentiation. Here, we show that p53, one of the most commonly inactivated tumor suppressors in glioma, is a gatekeeper of astrocyte fate. In the context of stab-wound injury, p53 loss destabilized the identity of astrocytes, priming them to dedifferentiate in later life. This resulted from persistent and age-exacerbated neuroinflammation at the injury site and EGFR activation in periwound astrocytes. Mechanistically, dedifferentiation was driven by the synergistic upregulation of mTOR signaling downstream of p53 loss and EGFR, which reinstates stemness programs via increased translation of neurodevelopmental transcription factors. Thus, our findings suggest that first-hit mutations remove the barriers to injury-induced dedifferentiation by sensitizing somatic cells to inflammatory signals, with implications for tumorigenesis.

## Highlights

• The tumor suppressor p53 restricts injury-induced plasticity of cortical astrocytes
• p53 loss destabilizes astrocyte identity in the context of injury in early life
• Increased neuroinflammation at the injury site drives dedifferentiation upon aging
• EGFR activation by injury signals mediates dedifferentiation downstream of p53 loss

## Graphical abstract

**Figure:**
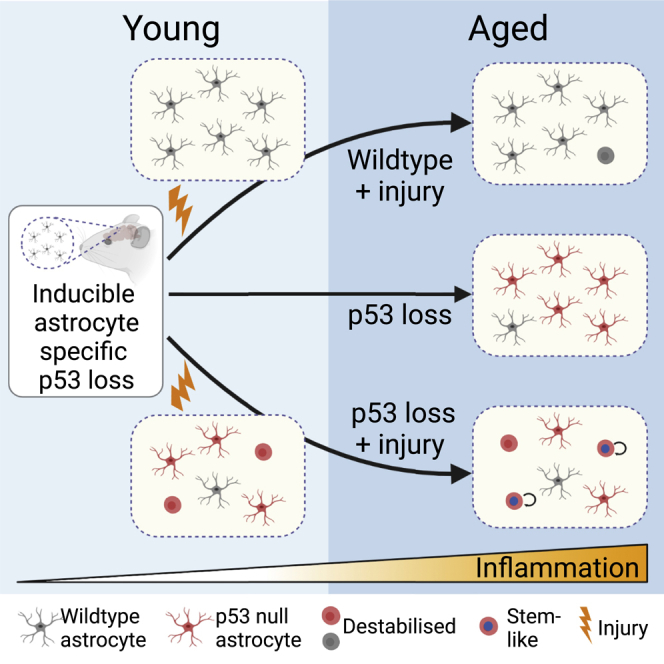

## Teaser

Simpson Ragdale et al. show that p53 inactivation, a common event in glioma, remains silent in healthy cortical astrocytes but leads to dedifferentiation to a neural stem-cell-like state upon injury. This results from p53 loss sensitizing astrocytes to neuroinflammation, which is induced by early-life injury and potentiated by aging.

## Introduction

Parenchymal astrocytes retain a latent neurogenic program, which can be reactivated by brain injury.^1^^,^^2^^,^^3^^,^^4^^,^^5^ Striatal astrocytes were shown to convert to cells with neural stem cell properties (NSC-like cells) and generate new neurons in response to excitotoxic damage or stroke, in a Notch-dependent manner.^2^^,^^3^^,^^4^ Consistently, the deletion of *Rpbj-k* elicited astrocyte neurogenesis following cortical stab-wound injury.^1^ However, in the wild-type brain, the majority of astrocyte subtypes remain within their lineage upon injury. This includes cortical astrocytes, which upregulate stemness markers, but fail to dedifferentiate *in vivo*, despite doing so when dissociated and cultured *in vitro*.^5^^,^^6^

In contrast, the astrocyte lineage barrier is fully subverted in tumorigenesis. Inactivation of the tumor suppressor protein p53 and overexpression of oncogenic H-RasV12 dedifferentiated cortical astrocytes to tumor-initiating glioma stem cells.^7^ Furthermore, within established gliomas, differentiation of astrocyte-like cells is partial and unstable.^8^^,^^9^ However, whether glioma-relevant genes play a role in controlling astrocyte plasticity in the normal brain remains unclear.

It is also poorly understood how the disruption of these genes may intersect with the dedifferentiation-promoting injury microenvironment. This is of relevance to glioma, as injury programs have emerged as major players in disease progression and recurrence, which develops within the inflammatory post-treatment microenvironment.^10^^,^^11^^,^^12^ In this context, p53 is a particularly interesting candidate as it inhibits somatic cell reprogramming^13^^,^^14^^,^^15^ and is one of the most commonly inactivated tumor suppressors in glioma.^16^ In the adult subventricular zone (SVZ) neurogenic niche, p53 modulates neurogenesis by suppressing the proliferation of transit-amplifying progenitors and their differentiation to neuroblasts.^17^^,^^18^^,^^19^ Furthermore, our analysis of the Zamboni et al. dataset^1^ comparing wild-type astrocytes from healthy or stab-wound-injured cortices revealed a significant enrichment of p53 signatures in injured astrocytes (Figure S1A). A parallel enrichment was also observed in reactive human astrocytes in the Li et al. dataset^20^ (Figure S1B). Together, this evidence suggests that p53 may function as a barrier to injury-induced astrocyte dedifferentiation.

In this study, we explored the role of p53 in the response of cortical astrocytes to stab-wound injury in young adulthood and the long-term impact of its deletion on astrocyte fate.

## Results

### Acute p53 loss synergizes with injury signals to downregulate astrocyte lineage identity *in vivo*

**Figure 1:**
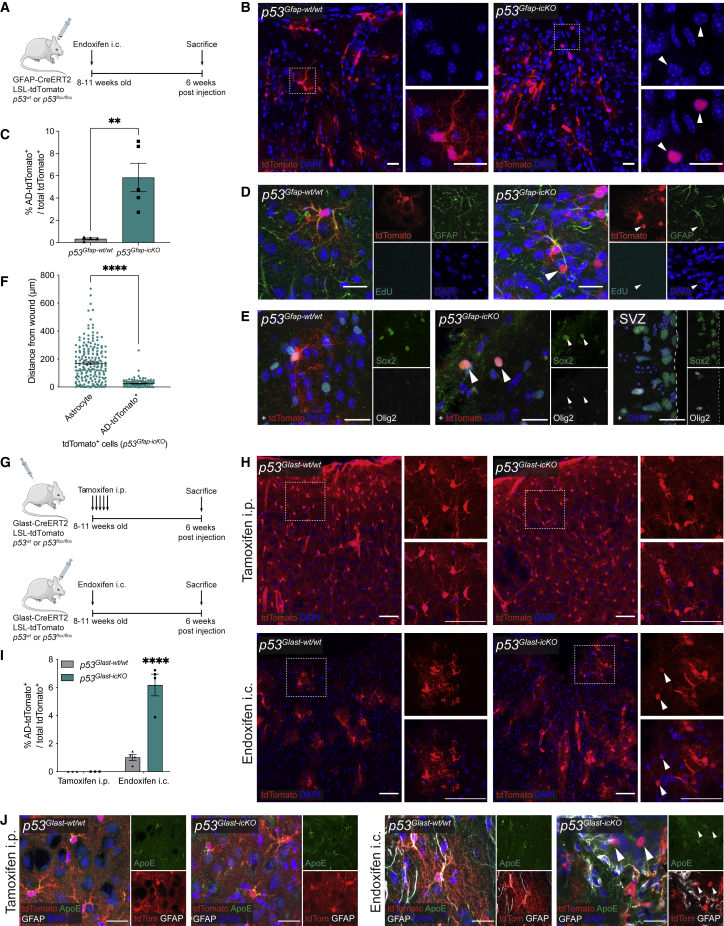
p53 loss and injury synergize to downregulate astrocyte lineage identity *in vivo* (A) Schematic of experimental outline. Endoxifen was injected intracranially (i.c.) into the cortex of 8- to 11-week-old *GFAP-CreERT2; LSL-tdTomato; p53*^*wt/wt*^ (*p53*^*Gfap-wt/wt*^) or *GFAP-CreERT2; LSL-tdTomato; p53*^*flox/flox*^ (*p53*^*Gfap-icKO*^) mice. 6 weeks postinjection, mice were sacrificed, and fate-mapped astrocytes (tdTomato^+^) were assessed by immunostaining. (B) Representative images of tdTomato^+^ lineage-traced cells in indicated genotypes. Astrocyte-derived rounded cells without processes (AD-tdTomato^+^) are indicated by white arrowheads. Scale bars, 20 μm. (C) Quantification of AD-tdTomato^+^ cells as a percentage of total tdTomato^+^ cells. (Mean ± SEM; *p53*^*Gfap-wt/wt*^, n = 4; *p53*^*Gfap-icKO*^, n = 5. ^∗∗^p < 0.01, unpaired two-tailed t test.) (D and E) Representative images of GFAP and EdU staining (D) and Olig2 and Sox2 staining (E). AD-tdTomato^+^ cells are negative for GFAP, EdU, and Olig2 but are Sox2^+^. In (E), subventricular zone (SVZ) is included as positive staining control. Scale bars, 20 μm. (F) Quantification of the perpendicular distance of cells from the injury site in n = 4 *p53*^*Gfap-icKO*^ animals. (Mean ± SEM; ^∗∗∗∗^p < 0.0001, unpaired two-tailed t test.) (G) Schematic of experimental outline. Tamoxifen was injected intraperitoneally (i.p.) or endoxifen was injected i.c. into the cortex of 8- to 11-week-old *Glast-CreERT2; LSL-tdTomato; p53*^*wt/wt*^ (*p53*^*Glast-wt/wt*^) or *Glast-CreERT2; LSL-tdTomato; p53*^*flox/flox*^ (*p53*^*Glast-icKO*^) mice. 6 weeks postinjection, mice were sacrificed, and fate-mapped astrocytes (tdTomato^+^) were assessed by immunostaining. (H) Representative images of tdTomato^+^ lineage-traced cells in indicated conditions. White dashed box indicates location of higher magnification image (right). Representative AD-tdTomato^+^ cells are indicated by white arrowheads. Scale bars, 50 μm. (I) Quantification of AD-tdTomato^+^ cells as a percentage of the total tdTomato^+^ cells. (Mean ± SEM; tamoxifen i.p., n = 3, and endoxifen i.c., n = 4 for each genotype. ^∗∗∗∗^p < 0.0001 *p53*^*Glast-icKO*^ endoxifen i.c. compared with each other condition. Comparisons of all other conditions were not significant [p > 0.05], two-way ANOVA with Tukey’s multiple-comparisons test.) (J) Representative images of GFAP and ApoE staining in indicated conditions. Representative AD-tdTomato^+^ cells are indicated by white arrowheads. Scale bars, 20 μm. See also Figure S1.

To assess the impact of *p53* loss on adult cortical astrocytes in the context of injury, we generated a conditional and inducible mouse model driven by glial fibrillary acidic protein (GFAP), which is lowly or not expressed in most healthy cortical astrocytes, but upregulated upon injury during reactive astrogliosis.^21^^,^^22^^,^^23^ GFAP-CreERT2 mice were crossed to a conditional *p53* knockout line (*p53*^*flox/flox*^) alongside a tdTomato fluorescent reporter line (Rosa26:CAG-LoxP-STOP-LoxP-tdTomato or LSL-tdTomato) to generate *p53*^*Gfap-icKO*^ and recombination was induced in adult animals by direct stereotactic injection of endoxifen, an active metabolite of tamoxifen, into the cortex^23^^,^^24^^,^^25^^,^^26^ (Figure 1A). This enabled concomitant *p53* deletion and fate mapping of recombined cells and their progeny. It also ensured selective recombination of peri-injury cortical astrocytes in the absence of confounding recombination of SVZ progenitor cells (Figure S1C), which could migrate to the cortex and differentiate to astroglia *in situ*.^27^ The stab-wound injury caused by the needle itself was used as an injury model, as reported.^10^ *p53*^*wt/wt*^ mice crossed to GFAP-CreERT2 and LSL-tdTomato (*p53*^*Gfap-wt/wt*^) lines served as controls. 6 weeks after injection, a time at which astrocyte proliferation subsides and the injury response reaches a chronic remodeling phase,^28^ animals were sacrificed and tdTomato^+^ astrocytes assessed by immunostaining (Figure 1A). The majority of tdTomato^+^ cells retained the characteristic branched-astrocyte morphology in both *p53*^*Gfap-wt/wt*^ and *p53*^*Gfap-icKO*^ mice (Figure 1B). However, in *p53*^*Gfap-icKO*^ animals a significant proportion of recombined cells retracted their processes and became rounded (astrocyte-derived tdTomato^+^ cells or AD-tdTomato^+^) (Figures 1B and 1C). Importantly, immunostaining for RFP and cleaved caspase-3, respectively, confirmed that this phenotype was not due to loss of tdTomato fluorescence in the astrocyte processes (Figure S1D), nor was it due to cells dying (Figure S1E), but reflected a true shape change. AD-tdTomato^+^ cells were also not ectopically recombined oligodendrocyte progenitor cells (OPCs), as they did not co-express Olig2 and the OPC marker PDGFR-alpha (Figure S1F).

To determine whether this morphological change was accompanied by a fate change, we examined the expression of differentiation and stemness markers. AD-tdTomato^+^ cells had lost the mature astrocyte markers GFAP (Figure 1D) and ApoE (Figure S1G), but retained lineage markers Sox2 (Figure 1E) and nuclear factor I A (NFIA) (Figure S1G). Furthermore, AD-tdTomato^+^ cells remained negative for the stemness marker Olig2 and for Ascl1, a key marker of early astrocyte neurogenesis^2^^,^^3^ (Figures 1E and S1H), did not re-enter the cell cycle (Figures 1D and S1I) or acquire expression of the neuroblast marker doublecortin (DCX) (Figure S1H). Together, these results suggest that p53 loss destabilizes astrocyte identity following injury, but is insufficient to induce dedifferentiation or initiate neurogenesis at this early time point.

AD-tdTomato^+^ cells were found almost exclusively in the immediate vicinity of the wound (<100 μm), suggesting a dependency on injury signals, the levels of which are highest within this area^28^ (Figure 1F). To test this directly and rule out a general role in the maintenance of astrocyte fate, we examined effects of acute p53 loss on adult astrocytes in the intact cortex. To this end, we used a Glast-CreERT2 driver, which enables more efficient and widespread targeting of cortical astrocytes than the GFAP-CreERT2 line following systemic tamoxifen administration.^23^^,^^29^ Glast-CreERT2 mice and LSL-tdTomato were crossed to *p53*^*flox/flox*^ or *p53*^*wt/wt*^ animals to generate *p53*^*Glast-icKO*^ and *p53*^*Glast-wt/wt*^, respectively, and recombination was induced by intraperitoneal (i.p.) tamoxifen injections in adult animals (Figure 1G, top). In parallel, *p53*^*Glast-icKO*^ and *p53*^*Glast-wt/wt*^ were subjected to stereotactic intracortical endoxifen injections as described for the GFAP-CreERT2 line, to enable side-by-side comparison of p53 loss in healthy and injured brains (Figure 1G, bottom). As in *p53*^*Gfap-icKO*^ mice, we observed significant numbers of rounded AD-tdTomato^+^ that downregulated mature astrocyte markers in endoxifen-injected *p53*^*Glast-icKO*^ but not *p53*^*Glast-wt/wt*^ animals (Figures 1H–1J). This indicates that the destabilization of astrocyte fate is a general response of p53-deficient astrocytes to injury. In contrast, in uninjured, tamoxifen-injected mice of both genotypes, astrocyte morphology and identity remained unaltered (Figure 1H–1J). Thus, p53 restricts astrocyte plasticity selectively following injury and its loss synergizes with injury signals to destabilize astrocyte fate.

### The aging cortical microenvironment induces injury-primed p53 null astrocytes to dedifferentiate *in vivo*

**Figure 2:**
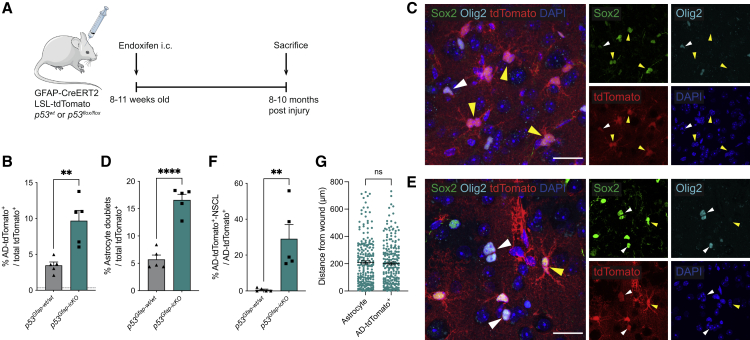
The aging microenvironment drives dedifferentiation of injury-primed p53-deficient cortical astrocytes *in vivo* (A) Schematic of experimental outline. Endoxifen was injected intracranially into the cortex of 8- to 11-week-old *GFAP-CreERT2; LSL-tdTomato; p53*^*wt/wt*^ (*p53*^*Gfap-wt/wt*^) or *GFAP-CreERT2; LSL-tdTomato; p53*^*flox/flox*^ (*p53*^*Gfap-icKO*^). 8–10 months postinjection, mice were sacrificed, and fate-mapped astrocytes (tdTomato^+^) were assessed by immunostaining. (B) Quantification of AD-tdTomato^+^ cells as a percentage of total tdTomato^+^ cells. (Mean + SEM; n = 5 per condition, ^∗∗^p < 0.01, unpaired two-tailed t test.) Dashed line indicates basal levels of AD-tdTomato^+^ cells in young injured *p53*^*Gfap-wt/wt*^ mice from Figure 1C. (C) Representative image of astrocytic tdTomato^+^ cell doublets (yellow arrowheads) and an Olig2^+^/Sox2^+^ AD-tdTomato^+^-NSCL cell (white arrowhead) in *p53*^*Gfap-icKO*^. Scale bars, 25 μm. (D) Quantification of tdTomato^+^ astrocyte doublets as a percentage of total tdTomato^+^ cells. (Mean + SEM; n = 5 per condition, ^∗∗∗∗^p < 0.0001, unpaired two-tailed t test.) (E) Representative image of Olig2^+^/Sox2^+^ AD-tdTomato^+^-NSCL cells (white arrowheads) in *p53*^*Gfap-icKO*^, including a cell doublet indicative of recent division. Scale bars, 25 μm. (F) Quantification of AD-tdTomato^+^-NSCL cells as a percentage of AD-tdTomato^+^ cells. (Mean + SEM; n = 5 per condition; ^∗∗^p < 0.01, unpaired two-tailed t test.) (G) Quantification of the perpendicular distance of cells from the injury site in n = 5 aged injured *p53*^*Gfap-icKO*^ animals. (Mean ± SEM; ns, not significant; Mann-Whitney test.) See also Figure S2.

We reasoned that although at 6 weeks postinjury p53-deficient astrocytes underwent a modest change in identity, their fate may be further affected over a longer time period. We therefore examined the cortices of *p53*^*Gfap-wt/wt*^ and *p53*^*Gfap-icKO*^ mice at 8–10 months following endoxifen intracortical injections, corresponding to mice of >1 year of age (Figure 2A). Interestingly, we found an increase in the proportion of AD-tdTomato^+^ cells in both genotypes, suggesting that the aging tissue microenvironment is sufficient to drive partial loss of astrocyte identity following early-life injury, even without *p53* deletion (Figure 2B). However, *p53*^*Gfap-icKO*^ injured cortices displayed an increase in astrocyte doublets, which is indicative of recent astrocyte proliferation (Figures 2C and 2D) and, more strikingly, uniquely contained a subset of AD-tdTomato^+^ cells that co-expressed the stemness and transit-amplifying progenitor markers Sox2, Olig2, and Ascl1, indicating that they may have acquired stem-like characteristics (Figures 2C, 2E, 2F, and S2A). These cells were also lowly proliferative, in that they occasionally appeared as doublets (Figure 2E) or were positive for the proliferation marker Ki67 (Figure S2B). Remarkably, we also detected rare Sox2^−^, Olig2^−^, and NeuN^+^ tdTomato^+^ cells with a neuronal morphology selectively in the aged *p53*^*Gfap-icKO*^-injured brains, suggesting that some p53-deficient astrocytes may acquire neurogenic potential (Figures S2C and S2D). These data indicate that upon aging, p53-deficient AD-tdTomato^+^ progress to a dedifferentiated neural stem-cell-like state (AD-tdTomato^+^-NSCL cells).

Importantly, these phenotypes were not a general consequence of aging, as we did not detect AD-tdTomato^+^ cells, astrocyte doublets or AD-tdTomato^+^-NSCL cells in aged-matched uninjured *p53*^*Glast-wt/wt*^ or *p53*^*Glast-icKO*^ animals 8–10 months following systemic tamoxifen administration (Figures S2E–S2H). Thus, injury in early life primes p53-deficient astrocytes to dedifferentiate upon aging.

### Exacerbated neuroinflammation at the wound site underlies age-dependent dedifferentiation of p53-deficient astrocytes following early-life injury

We next sought to understand what properties of the aging brain microenvironment may render it permissive to full dedifferentiation of AD-tdTomato^+^ cells. Increased neuroinflammation is a hallmark of aging.^30^^,^^31^ Furthermore, the positioning of AD-tdTomato^+^ cells near the needle tract at 6 weeks postinjury is suggestive of a role for inflammatory signals (Figure 1F) and qPCR analysis of young and old cortical astrocytes revealed a small but significant increase in p53 levels, which is indicative of shared inflammation-related mechanisms between injury and aging responses (Figure S3A). We therefore hypothesized that later-life dedifferentiation may be driven by age-dependent neuroinflammation.

To test this, we first examined resident microglia, which play a major role in age-dependent neuroinflammation.^30^ Iba-1 and CD68 immunofluorescence analysis was carried out in intact or injured cortices at 6 weeks and 10 months after injury to examine microglia morphology and activation, respectively (Figures S3B–S3D). As expected, this revealed that microglia acquired morphological changes associated with activation and priming, such as deramification and shortening of their cellular processes, in the peri-injury regions (both proximal and distal to the needle tract) of both young and old brains, as well as in intact old brains (Figures S3B and S3C). However, CD68 levels differed between groups, with microglia in the proximal wound displaying the highest CD68 expression regardless of age, and a selective increase in expression in the distal wound region of old animals (Figure S3D). These observations suggest that the inflammatory response to a stab-wound injury in early life exacerbates age-dependent neuroinflammation, resulting in more extensive and pronounced inflammation in the periwound region than either injury in the young brain or aging alone. To understand how these changes might contribute to later-life dedifferentiation, we examined the distribution of AD-tdTomato^+^ cells at the wound site in aged animals. Unlike in young mice, where destabilization of astrocyte fate only occurred adjacent to the needle tract (Figure 1F), at 8–10 months postinjection we found AD-tdTomato^+^ cells in both proximal and distal wound regions (Figure 2G). Together, these findings are consistent with microglia-mediated inflammation promoting dedifferentiation of p53-deficient astrocytes.

**Figure 3:**
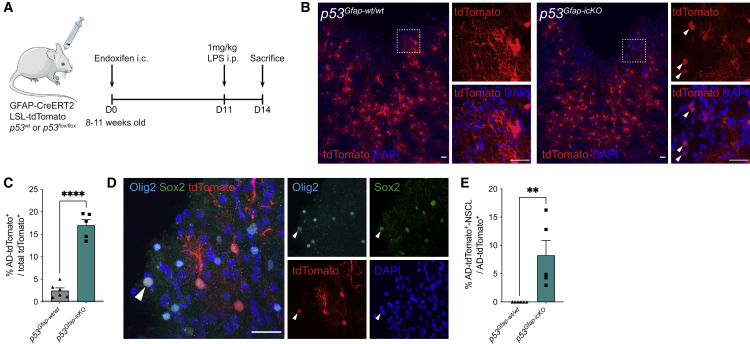
Increased neuroinflammation underlies age-dependent dedifferentiation of p53-deficient astrocytes following early-life injury (A) Schematic of experimental outline. Endoxifen was injected intracranially into the cortex of 8- to 11-week-old *GFAP-CreERT2; LSL-tdTomato; p53*^*wt/wt*^ (*p53*^*Gfap-wt/wt*^) or *GFAP-CreERT2; LSL-tdTomato; p53*^*flox/flox*^ (*p53*^*Gfap-icKO*^) mice. 11 days later, lipopolysaccharide (LPS, 1 mg/kg) was injected intraperitoneally (i.p.) to induce peripheral inflammation and microglial activation. 3 days later, mice were sacrificed, and fate-mapped astrocytes (tdTomato^+^) were assessed by immunostaining. (B) Representative images of tdTomato^+^ lineage-traced cells in indicated genotypes after endoxifen and LPS injection. Astrocyte-derived rounded cells without processes (AD-tdTomato^+^) are indicated by white arrowheads. Scale bars, 25 μm. (C) Quantification of AD-tdTomato^+^ cells as a percentage of total tdTomato^+^ cells. (Mean + SEM; LPS *p53*^*Gfap-wt/wt*^, n = 6; LPS *p53*^*Gfap-icKO*^, n = 5; ^∗∗∗∗^p < 0.0001, unpaired two-tailed t test.) Dashed line indicates basal levels of AD-tdTomato^+^ cells in young injured *p53*^*Gfap-wt/wt*^ mice from Figure 1C. (D) Representative image of AD-tdTomato^+^-NSCL cells (white arrowheads) in LPS injected *p53*^*Gfap-icKO*^. Scale bars, 25 μm. (E) Quantification of AD-tdTomato^+^-NSCL cells as a percentage of AD-tdTomato^+^ cells. (Mean + SEM; LPS *p53*^*Gfap-wt/wt*^, n = 6; LPS *p53*^*Gfap-icKO*^, n = 5; ^∗∗^p < 0.01, unpaired two-tailed t test.) See also Figure S3.

To test this hypothesis, we next performed gain-of-function studies. Young *p53*^*Gfap-wt/wt*^ and *p53*^*Gfap-icKO*^ mice were subjected to stab-wound injury alongside intraperitoneal (i.p.) administration of lipopolysaccharide (LPS), which increases neuroinflammation through activation of microglia.^32^^,^^33^^,^^34^ LPS was administered at day 11 postinjury and animals were sacrificed 3 days later to achieve maximal amplification of the endogenous inflammatory response (which peaks between 1 and 2 weeks postinjury) while avoiding systemic LPS-induced toxicity^28^ (Figure 3A). As expected, this treatment regime resulted in microglia activation throughout the brain,^33^^,^^34^ which was further increased at the injury site and greater than matched PBS-treated controls (Figure S3E). We found that the combination of stab-wound injury, p53 loss, and microglia-mediated inflammation was sufficient to recapitulate the phenotype of injured and aged *p53*^*Gfap-icKO*^ brains, leading to the formation of AD-tdTomato^+^ cells, a subset of which were lowly proliferative AD-tdTomato^+^-NSCL cells (Figures 3B–3E and S3F–S3H). As in aged brains, LPS also induced AD-tdTomato^+^ cells in wild-type brains, confirming that partial loss of astrocyte identity in the absence of mutations depends on inflammation (Figure 3C). Together, these findings suggest that early-life injury synergizes with age-related inflammation to convert the cortical microenvironment to a permissive milieu for the dedifferentiation of p53-deficient astrocytes.

### Dedifferentiation of p53-deficient astrocytes is EGFR dependent

**Figure 4:**
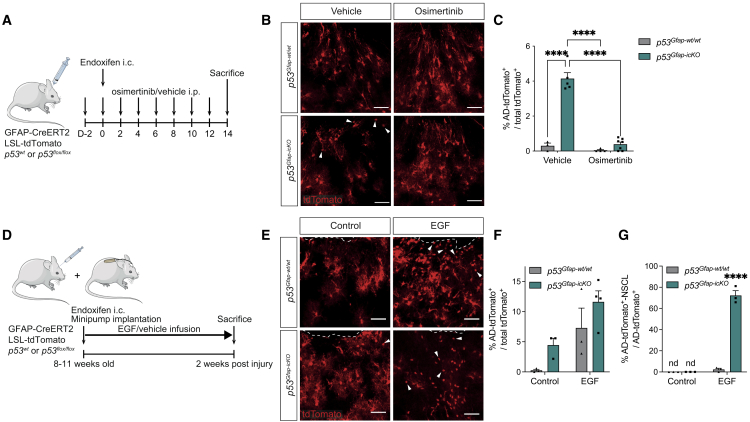
Dedifferentiation of p53-deficient astrocytes is EGFR dependent (A) Schematic of experimental outline. *GFAP-CreERT2; LSL-tdTomato; p53*^*wt/wt*^ (*p53*^*Gfap-wt/wt*^) or *GFAP-CreERT2; LSL-tdTomato; p53*^*flox/flox*^ (*p53*^*Gfap-icKO*^) mice were injected intracranially (i.c.) with endoxifen into the cortex to induce astrocyte-specific *p53* recombination and tdTomato labeling. Mice were also i.p. injected with osimertinib or vehicle control 2 days before i.c. injection, and every other day thereafter. Mice were sacrificed 2 weeks post i.c. injection and fate-mapped (tdTomato^+^) astrocytes were assessed. (B) Representative images of tdTomato^+^ fate-mapped cells in osimertinib experiment. White arrowheads indicate AD-tdTomato^+^ cells. Scale bars, 50 μm. (C) Quantification of percentage of AD-tdTomato^+^ cells as a percentage of the total tdTomato^+^ cells. *p53*^*Gfap-wt/wt*^ vehicle, n = 3; *p53*^*Gfap-icKO*^ vehicle, n = 5; *p53*^*Gfap-wt/wt*^ osimertinib, n = 4; *p53*^*Gfap-icKO*^ osimertinib, n = 7. (D) Schematic of experimental outline. 8- to 11-week-old *GFAP-CreERT2; LSL-tdTomato; p53*^*wt/wt*^ (*p53*^*Gfap-wt/wt*^) or *GFAP-CreERT2; LSL-tdTomato; p53*^*flox/flox*^ (*p53*^*Gfap-icKO*^) mice were stereotactically injected i.c. with endoxifen into the cortex to induce astrocyte-specific *p53* recombination and tdTomato labeling. Infusion cannula attached to an osmotic minipump continuously infused EGF or vehicle control into injection site. After 2 weeks of infusion, mice were sacrificed, and fate-mapped (tdTomato^+^) astrocytes were assessed by immunostaining. (E) Representative image of tdTomato^+^ cell morphology. White arrowheads indicate representative AD-tdTomato^+^ cells. Scale bars, 50 μm. (F) Quantification of AD-tdTomato^+^ cells as a percentage of the total tdTomato^+^ cells. (n = 3 per condition except *p53*^*Gfap-icKO*^ EGF, n = 4.) (G) Quantification of AD-tdTomato^+^-NSCL cells as a percentage of AD-tdTomato^+^ cells. (n = 3 per condition). (For all graphs, mean + SEM; nd, not detected; ns, not significant; ^∗∗∗∗^p < 0.0001, two-way ANOVA with Tukey’s multiple-comparisons test.) See also Figures S3 and S4.

We next sought to identify the signaling pathways that respond to inflammatory signals to drive dedifferentiation of p53 deficient astrocytes. Epidermal growth factor receptor (EGFR) signaling underpins the activation of quiescent NSCs^35^ and increases neurogenesis of *Rbpj-k*-deficient striatal astrocytes.^3^ Furthermore, EGF levels increase in the peri-injury brain microenvironment.^21^ We therefore examined the role of EGFR through a series of complementary experiments. First, we compared expression levels of EGFR ligands in injured and contralateral cortices from young brains, as well as in uninjured young and old cortices (Figures S3I and S3J) and found a significant increase relative to controls in both. A similar increase was also observed in published datasets of microglia activation following LPS treatment^36^ or upon aging,^37^ consistent with EGFR signaling potentially responding to neuroinflammation (Figures S3K and S3L). Second, we carried out gain- and loss-of-function experiments. Young adult *p53*^*Gfap-wt/wt*^ and *p53*^*Gfap-icKO*^ mice were injected intracranially (i.c.) with endoxifen and treated with osimertinib, a selective EGFR inhibitor, or vehicle control for 2 weeks (Figure 4A), which effectively dampened EGFR signaling in the periwound region (Figures S4A and S4B). We found that osimertinib reduced the percentage of AD-tdTomato^+^ cells in young adult *p53*^*Gfap-icKO*^ mice to *p53*^*Gfap-wt/wt*^ control levels, indicating that increased EGFR signaling is necessary for the destabilization of astrocyte identity following p53 loss (Figures 4B and 4C). To test if EGFR activation is also sufficient, we infused recombinant EGF, or saline control, to the injury site of young adult mice through osmotic minipumps implanted at the time of intracortical endoxifen administration (Figure 4D). Strikingly, EGF infusion phenocopied p53 effects observed in both young and aged brains: in *p53*^*Gfap-wt/wt*^ mice, it induced the formation of AD-tdTomato^+^ cells to an extent similar to p53 deletion in young injured brains (Figures 4E and 4F); in *p53*^*Gfap-icKO*^ mice it increased the overall proportion of AD-tdTomato^+^ cells and, as in the aged and LPS-treated brain, selectively led to their progression to AD-tdTomato^+^-NSCL cells, as judged by the presence of doublets, Ki67^+^ and Sox2^+^/Olig2^+^/Ascl1^+^ AD-tdTomato^+^ cells (Figures 4E–4G and S4C–S4H). Together, these experiments indicate that p53 phenotypes are EGFR dependent.

### scRNA-seq reveals temporal progression of p53-induced astrocyte dedifferentiation

To explore the mechanisms by which p53 loss and EGFR signaling synergize to drive dedifferentiation, we turned to an established and more tractable *in vitro* model. Primary astrocytes were purified from the cortices of postnatal day 3 *p53*^*flox/flox*^; LSL-tdTomato mice,^38^ recombined *in vitro* using GFAP-Cre or empty vector adenoviruses to generate *p53*^*−/−*^ and *p53*^*+/+*^ astrocytes, respectively, and cultured for 7 days in EGF-containing NSC media to mimic the injured microenvironment. Parallel cultures were maintained in bone morphogenetic protein 4 (BMP4) to mimic the prodifferentiative signals of the intact cortex^39^ (Figure S4I, top). Clonogenic assays were then used to assess dedifferentiation. Similar to our observations *in vivo*, p53 deletion did not dedifferentiate astrocytes in BMP4, nor did EGF treatment of p53 wild-type cells (Figure S4J). However, p53 deletion combined with EGF-induced dedifferentiation to cultured NSC-like cells, as indicated by the appearance of discreet colonies of small tdTomato^+^ cells, which acquired Ascl1 and Olig2 protein expression, strongly increased Sox2 protein levels, and were highly proliferative (Figures S4J–S4N). Furthermore, replating of EGF-treated *p53*^*−/−*^, but not *p53*^*+/+*^, cultures in suspension formed neurospheres capable of self-renewal and multilineage differentiation (Figures S4O–S4Q). To determine whether these findings are relevant to human astrocytes, we repeated these experiments in primary human astrocytes (and human NSC-derived astrocytes) using lentiviral shRNA constructs to acutely downregulate p53 (Figures S4R–S4X). We found all mouse phenotypes to be reproduced in human cells, with p53-knockdown (but not control-vector transduced) astrocytes dedifferentiating to NSC-like cells in the presence of EGF and retaining their astrocytic phenotype in BMP4. Thus, our *in vitro* models recapitulate *in vivo* phenotypes to a large extent and support the notion that p53 deletion synergizes with EGFR signaling to dedifferentiate cortical astrocytes to NSC-like cells.

**Figure 5:**
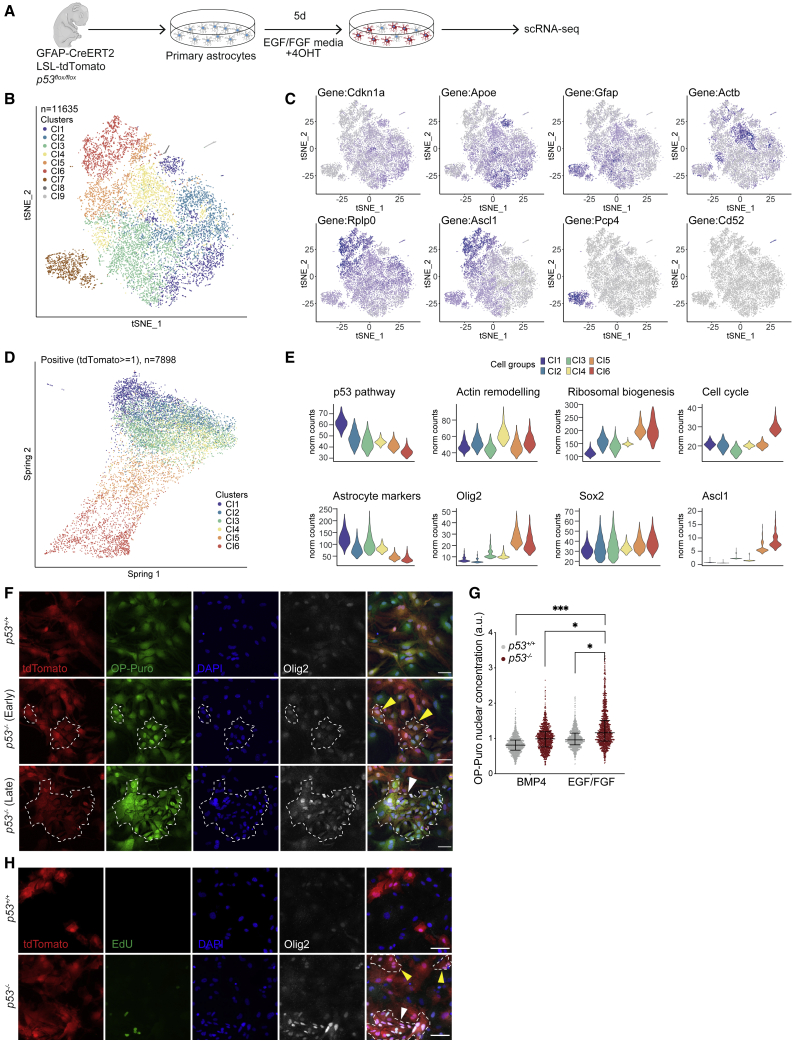
scRNA-seq reveals temporal progression of astrocyte dedifferentiation *in vitro* (A) Schematic of experimental outline. Primary cortical astrocytes were isolated from postnatal P3 *GFAP-CreERT2; LSL-tdTomato; p53*^*flox/flox*^ (*p53*^*Gfap-icKO*^) pups. Following immunopanning, astrocytes were incubated with 4-hydroxytamoxifen (4OHT) in EGF/FGF media for 5 days to induce *p53* and tdTomato allele recombination and astrocyte dedifferentiation, before collection for 10x Genomics scRNA-seq. (B) t-SNE plot of all cells. (C) Expression of key upregulated gene per cluster shown on t-SNE plots as in (B). (D) SPRING analysis of cells dynamic trajectories, after filtering to remove contaminant clusters (Cl7–9) and tdTomato negative cells (tdTomato reads = 0). (E) Violin plots of pathway analysis (top) or indicated genes (bottom) in ordered clusters. (F) Representative images of OP-puro labeling and Olig2 staining in EGF/FGF media. Shown is the increased protein synthesis (OP-puro labeling) and Olig2 expression in both early (yellow arrowhead, smaller cell clusters) and late (white arrowhead, larger cell clusters) dedifferentiated colonies in *p53*^*−/−*^ cultures, defined by change in cell size and morphology relative to astrocytes. Colonies are marked by dashed lines. Scale bars, 50 μm. (G) Quantification of OP-puro nuclear protein staining intensity from (F). Each point represents an individual cell. Line represents median with interquartile range. (n = 3 independent experiments; ^∗^p < 0.05, ^∗∗∗^p < 0.001; two-way ANOVA with Tukey’s multiple-comparisons test on average intensity per condition per replicate.) (H) Representative images of Olig2 staining and EdU incorporation in *p53*^*+/+*^ and *p53*^*−/−*^ cultures in EGF/FGF media. Late Olig2^+^ clusters (white arrowheads) are highly proliferative and correspond to Cl6 cells. Scale bars, 50 μm. See also Figures S4 and S5.

Having validated our *in vitro* system, we next sought to define the cellular mechanisms that underlie p53-dependent astrocyte dedifferentiation using scRNA-seq. To enable analysis of cells at various stages of dedifferentiation, we prepared primary postnatal cortical astrocytes from *p53*^*Gfap-icKO*^ mice and induced recombination *in vitro* using 4-hydroxytamoxifen (4OHT) for 5 days in the presence of EGF (Figures 5A and S4I, bottom), which leads to heterogeneous recombination times. As expected, this model also resulted in astrocyte dedifferentiation to NSC-like cells selectively in p53 deleted astrocytes exposed to EGF and was therefore equivalent to the adenoviral system (Figures S4J and S5A).

We analyzed whole transcriptomes of 11,635 single cells derived from two independent cultures on two separate 10x Chromium runs. As these did not show strong batch effects or differences in tdTomato recombination frequencies, they were pooled for further analysis (Figures S5B–S5D); t-distributed stochastic neighbor embedding (t-SNE) analysis of the combined datasets revealed three distinct cell populations that were further divided into 9 clusters (Figure 5B). After removing low-quality cells (cluster 8, Figures 5C and S5E), cells likely contaminating CD52^+^ microglia (cluster 9, Figures 5C and S5E), PCP4^+^ OPCs (cluster 7, Figures 5C, S5E, and S5F) and unrecombined cells, based on cell-type marker expression or absence of tdTomato reads, respectively, 7,898 cells remained. These encompassed clusters 1–6, on which the rest of the analysis was focused. Clusters 1–4 expressed astrocyte markers to varying degrees, including *Apoe* and *Gfap* (Figures 5C and S5E), likely reflecting the heterogeneity of cortical astrocytes *in vivo*.^40^^,^^41^ Cluster 4 displayed a signature related to cytoskeleton remodeling, including *Actb* upregulation (Figures 5C and S5E). Interestingly, this cluster still retained expression of the p53 target *Cdkn1a*, suggesting that this signature may be a p53-independent response, possibly linked to mitogenic stimulation (Figure 5C). Consistently, exposure to EGF and FGF induced a dramatic change in the actin cytoskeleton of both *p53*^*+/+*^ and *p53*^*−/−*^ cultures previously maintained in BMP4 (Figure S5G). Clusters 5 and 6 had signatures of ribosomal biogenesis and stemness (including *Ascl1* and *Olig2* expression), with cluster 6 also displaying a strong increase in proliferation signatures (Figures 5E and S5E). These two clusters therefore likely contained dedifferentiating astrocytes and astrocyte-derived NSC-like cells. Importantly, both expressed low levels of *Cdkn1a* (Figure 5C), which was accompanied by the strongest downregulation of p53 pathway signatures in cluster 6 (Figure 5E), in line with our *in vivo* findings supporting a role for p53 as a gatekeeper of astrocytic fate.

We then asked whether an ordered dedifferentiation path could be found in our data using SPRING^42^ (Figures 5D and S5J), pseudotime (monocle3, Figure S5H) and velocity (Figures S5I and S5J). All approaches converged toward a model where astrocytes exist in four interconnected states with no clear directionality, likely due to the aforementioned heterogeneity of the starting cultures (clusters 1–4). From these states, dedifferentiating cells downregulated astrocyte markers while concomitantly increasing expression of ribosomal genes and stemness markers (cluster 5), prior to progressing to a highly proliferative NSC-like state reminiscent of normal active NSC or transit-amplifying progenitors (cluster 6) (Figure 5E). These observations suggest that p53-dependent astrocyte dedifferentiation closely mirrors the progression of normal quiescent SVZ NSCs to an active state, which includes an intermediate state of primed quiescence, in which cells increase protein synthesis in preparation for neurogenesis.^43^^,^^44^ To confirm this timeline and that ribosomal gene signatures reflect changes in protein synthesis, we performed O-propargyl-puromycin (OP-puro) incorporation experiments (Figures 5F and 5G). High Olig2 expression was used as a selective marker of clusters 5 and 6, and an 5-ethynyl-2'-deoxyuridine (EdU) pulse prior to analysis enabled correlation to proliferation rate (Figure 5H). We detected two types of Olig2^high^ colonies in the cultures, which consisted of small nonastrocytic tdTomato^+^ cells: smaller “early” colonies of 3–15 cells, which were largely EdU^−^ and larger, more compact “late” colonies of >15 cells that incorporated EdU (Figures 5F–5H). Importantly, both types of colonies displayed elevated OP-puro, regardless of their proliferative status. These results are consistent with the pseudotime analysis, placing increased protein synthesis upstream of cell proliferation during dedifferentiation. The ability to dedifferentiate to NSC-like cells through these mechanisms was not unique to postnatal astrocytes, which retain stemness potential.^45^ Rather, it was also conserved in adult cells, as judged by scRNA-seq analysis of adult cortical-astrocyte preparations *in vitro* and, crucially, from tdTomato^+^ cells acutely FACS-sorted from injured and aged brains of *p53*^*Gfap-wt/wt*^ and *p53*^*Gfap-icKO*^ mice (Figure S5K). Indeed, integration of postnatal and adult scRNA-seq datasets using the Harmony batch-correction algorithm^46^ identified dedifferentiated cells that clustered with postnatal astrocyte-derived NSC-like cells in both *in vitro* and *in vivo* adult populations (Figure S5K). *In vitro*, NSC-like cells (cluster A2) formed via downregulation of astrocyte markers and increasing ribosomal biogenesis prior to cell-cycle re-entry (Figures S5L and S5M), and *in vivo* were observed selectively in *p53*^*Gfap-icKO*^ (Figures S5K*iv* and S5K*v*) at a frequency consistent with our immunohistochemistry analyses (see Figures 2B and 2F). Thus, p53 loss reactivates a neurogenic response in cortical astrocytes that mimics the transition from quiescent to activated SVZ NSCs.

### p53 loss drives dedifferentiation through derepression of mTOR-mediated translation of stemness transcription factors

**Figure 6:**
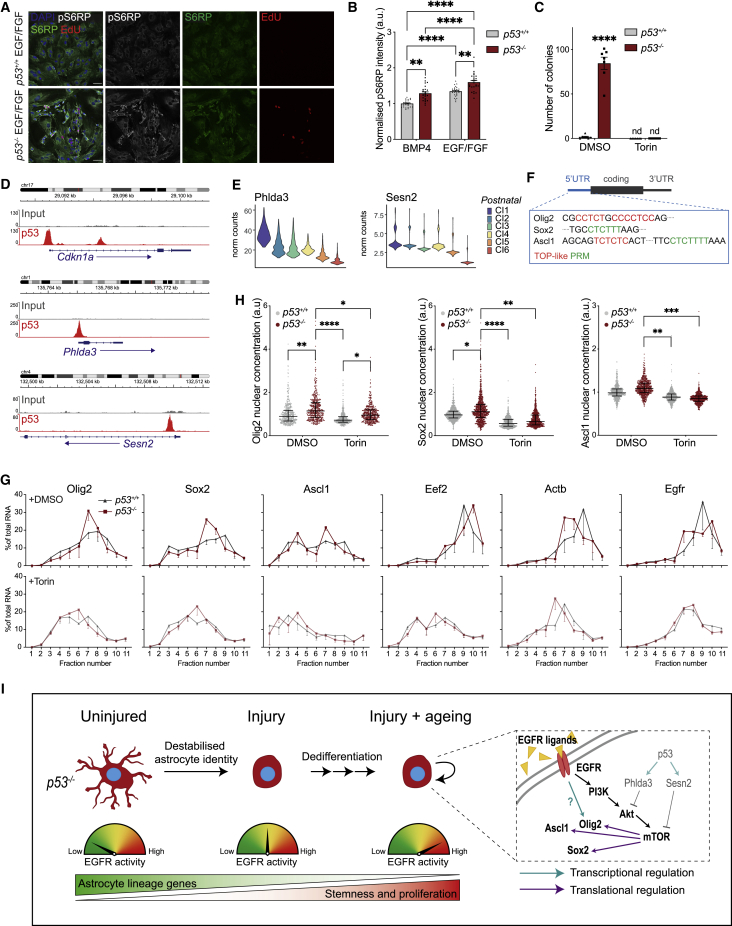
Increased mTOR activity downstream of p53 loss and EGFR activation leads to increased translation of stemness transcription factors (A) Representative images of phospho-S6 ribosomal protein (pS6RP) and S6RP staining in astrocytes cultured in EGF/FGF. Colonies of dedifferentiated cells have higher pS6RP staining. Scale bars, 50 μm. (B) Quantification of pS6RP intensity normalized to total S6RP shown in (A). Pixel intensity of cellular cytoplasmic mask per whole field of view was quantified for n = 3 independent experiments. Each point represents a single field of view. (Mean ± SEM; a.u., arbitrary units; ^∗∗^p < 0.01, ^∗∗∗∗^p < 0.0001; two-way ANOVA with Tukey’s multiple-comparisons test.) (C) Quantification of number of dedifferentiated colonies visualized by crystal violet staining for *p53*^*+/+*^ or *p53*^*−/−*^ astrocytes cultured in EGF/FGF media supplemented with mTOR inhibitor, Torin, or vehicle control (DMSO). Torin treatment abolishes astrocyte dedifferentiation. (Mean ± SEM; nd, not detected; ^∗∗∗∗^p < 0.0001; two-way ANOVA with Tukey’s multiple-comparisons test. (D) ChIP sequencing of wild-type primary astrocytes shows p53 binding to canonical target *Cdkn1a* (positive control) and negative regulators of the mTOR pathway *Phlda3* and *Sesn2*. (E) Violin plots of indicated genes in the ordered clusters from postnatal *in vitro* scRNA-seq data. Expression of *Phlda3* and *Sesn2* decreases sequentially with p53 pathway inactivation (as in Figure 5E). (F) Terminal oligopyrimidine (TOP)-like motif or pyrimidine-rich motif (PRM) identified in 5′ UTRs of indicated genes. (G) Distribution of mRNAs of indicated genes across the gradient fractions of the polysome profiles of *p53*^*+/+*^ or *p53*^*−/−*^ astrocytes cultured in EGF/FGF media in the absence (+DMSO) or presence of Torin (+Torin). (Mean ± SEM; n = 3 independent experiments). (H) Quantification of Olig2, Sox2, and Ascl1 nuclear protein staining intensity. Each point represents an individual cell. Line represents median with interquartile range. (n = 3 independent experiments; ^∗^p < 0.05, ^∗∗^p < 0.01, ^∗∗∗^p < 0.001, ^∗∗∗∗^p < 0.0001; two-way ANOVA with Tukey’s multiple comparisons test on average intensity per condition per replicate.) (I) Schematic of proposed mechanisms of astrocyte dedifferentiation. In uninjured brains where EGFR activity is low, derepression of mTOR upon p53 loss has no effect. Upon injury, the progressive increase in levels of EGFR ligands first destabilizes the identity of p53 null astrocytes in young adults and, in later life, causes their full dedifferentiation. This occurs through synergistic activation of mTOR signaling, which is switched on by EGFR and derepressed by p53 loss and drives translation of neurodevelopmental transcription factors to restore stemness programs in astrocytes. Teal and purple arrows indicate transcriptional and translational regulation, respectively. See also Figure S6.

Mammalian target of rapamycin (mTOR) is a central regulator of protein synthesis, and SVZ NSCs display elevated levels of mTOR-dependent translation.^47^ We therefore asked whether the detected increase in translation upon p53 loss was mTOR-dependent. Immunofluorescence analysis of *p53*^*+/+*^ and *p53*^*−/−*^ astrocyte cultures revealed a stark increase in phospho-S6 ribosomal protein (pS6RP), which was specific to *p53*^*−/−*^ NSC-like colonies and correlated with increased OP-puro incorporation (Figures 6A, 6B, and S6A). A similar increase in pS6RP was seen *in vivo* in AD-tdTomato^+^-NSCL cells, but not in tdTomato^+^ astrocytes (Figure S6B). Consistent with this, clusters 5 and 6 in postnatal, and corresponding clusters A3 and A2 in adult astrocytes, displayed elevated expression of mTOR and Myc signatures in the scRNA-seq datasets (Figure S6C). Interestingly, the increase in mTOR signaling in *p53*^*−/−*^ was greater in EGF, likely due to more robust activation of the pathway^48^ (Figure 6B). Furthermore, acute mTOR inhibition by Torin completely blocked dedifferentiation of *p53*^*−/−*^ astrocytes (Figures 6C and S6D). p53 is known to suppress mTOR signaling through direct transcriptional upregulation of Phlda3,^49^ Sestrin1, and Sestrin2.^50^ To determine whether any of these genes are direct p53 targets in astrocytes, we performed ChIP sequencing analysis in wild-type cells and correlated it to our scRNA-seq data. p53 bound the promoter regions of both *Phlda3* and *Sesn2* (Figure 6D), with both transcripts becoming strongly downregulated upon p53 loss in postnatal and adult astrocytes (Figures 6E and S6E). This is consistent with p53 loss derepressing mTOR signaling downstream of EGF to increase protein synthesis at the onset of dedifferentiation.

In adult neurogenesis, translational downregulation of Sox2 transcript by mTOR is necessary for stemness exit and neuronal differentiation.^47^ Selectivity is provided by a pyrimidine-rich motif (PRM), which is present in the 5′ UTR of the *Sox2* transcript.^47^ In our system, Sox2 protein, but not mRNA, levels increased significantly upon dedifferentiation (Figures 5E, S4M, and S4N). In addition, Ascl1 and Olig2 protein levels were much greater in late colonies than early ones, while mRNA levels were similar (Figures 5E, 5F, 5H, and S4M). We therefore hypothesized that mTOR-dependent translation of neurodevelopmental transcripts might underlie dedifferentiation by resetting astrocytes to a stem-like state. To test this hypothesis, we first examined the 5′ UTRs of *Ascl1* and *Olig2* mRNAs and found that both contained terminal oligopyrimidine (TOP)-like sequences,^51^ with *Ascl1* also containing a PRM region^47^ (Figure 6F). Second, we performed polysome fractionation experiments to measure ribosomal loading of *Sox2*, *Ascl1*, and *Olig2* transcripts in *p53*^*+/+*^ and *p53*^*−/−*^ astrocytes before and after Torin treatment (Figures 6G and S6F). This revealed a slightly higher proportion of *Olig2*, *Sox2*, and *Ascl1* transcripts in the polysomal fractions (fraction number >6) of *p53*^*−/−*^ relative to *p53*^*+/+*^ astrocytes, consistent with p53 loss promoting their translation. Furthermore, Torin treatment strongly repressed translation of all three transcripts, as judged by a pronounced shift toward the light and nontranslating polysome fractions, which was similar to that of the well-described mTOR-regulated transcript *Eef2*.^51^ In contrast, only a modest shift was observed in transcripts devoid of PRM and TOP motifs (*Actb* and *Egfr*), which was likely due to global decrease in protein translation upon mTOR blockade, rather than a transcript-selective effect.^51^ Finally, Torin treatment resulted in a parallel reduction in the levels of all three proteins in both wild-type and p53-deleted cells (Figure 6H). We conclude that p53 loss drives astrocyte dedifferentiation through downregulation of mTOR inhibitor genes, which leads to derepression of mTOR signaling downstream of EGFR. In turn, increased mTOR activity induces translation of stemness transcription factors to reinstate an NSC-like state (Figure 6I).

## Discussion

Cortical astrocytes are notoriously refractory to dedifferentiative cues *in vivo*, but the underlying mechanisms have remained unclear.^1^^,^^5^^,^^6^^,^^52^ Here we identified p53 as a key mediator. This is consistent with emerging functions of p53 in controlling cellular plasticity, for example, in cancer initiation, reprogramming, and epithelial-to-mesenchymal transition.^53^^,^^54^^,^^55^^,^^56^^,^^57^ However, while most of those functions have been attributed to cell-intrinsic p53 effects, our data reveal an unexpected dependence on microenvironmental cues. We found that p53 loss remained silent in healthy astrocytes and only following an injury and accompanying increase in neuroinflammation did astrocytes dedifferentiate. Thus, in analogy to cancer initiation, inflammatory cues then act as a “second hit” that enables the dedifferentiation of cells that have been made competent to respond to these cues by p53 loss.

Our findings are in line with increasing evidence that healthy tissues bear a variety of driver mutations, which are tolerated within a normal tissue microenvironment.^58^^,^^59^^,^^60^^,^^61^^,^^62^ They further suggest that it is a shift toward an inflammatory tissue microenvironment that enables mutated cells to express their malignant potential, with dedifferentiation to NSC-like cells and increased fate plasticity being key responses.

Notably, we identified EGFR as main receiver of dedifferentiative inflammatory signals. EGFR has long been known to play a fundamental role in controlling NSC decisions and suppressing differentiation.^35^^,^^48^^,^^63^ It is also a commonly amplified gene in glioblastoma.^64^ Our findings indicate that EGFR signaling acts as a rheostat in the control of astrocyte fate, whereby high signaling is required for dedifferentiation. Such levels cannot readily be achieved in wild-type cells, which converted to AD-tdTomato^+^ cells but failed to dedifferentiate even upon EGF infusion or injury-induced neuroinflammation. However, our data suggest that p53 loss lowers the threshold at which astrocytes respond to EGFR signaling, enabling their full conversion to the AD-tdTomato^+^-NSCL state within the injury microenvironment (Figure 6I). Thus, cell-extrinsic EGFR activation and cell-intrinsic p53 loss together create the “perfect storm” for subverting the astrocyte lineage barrier. Interestingly, although EGFR amplification and p53 alterations are both frequent mutations in glioblastoma, they rarely co-occur in patients.^16^^,^^64^ While more studies will be required to mechanistically unravel this mutual exclusivity, our findings may provide a partial explanation as they predict that p53 inactivation would be sufficient to hyperactivate wild-type EGFR, thus eliminating any selective pressure for EGFR mutations.

At the molecular level, we found the synergy between EGFR signaling and p53 loss to coalesce upon mTOR, which our *in vitro* data suggest reset stemness by inducing translation of neurodevelopmental transcription factors. It was recently shown that the differentiation of active NSCs to neuroblasts requires mTOR-dependent translational repression of stemness transcription factors, including Sox2.^47^ Thus, p53-dependent dedifferentiation of astrocytes may represent a reversal of normal developmental programs. Further conservation with normal neurogenesis was observed at the transcriptional level, where dedifferentiation programs closely mirrored the activation of quiescent NSCs to a proliferative state.^43^ This is consistent with recent findings and further underscores the notion that parenchymal astrocytes may represent dormant NSCs.^1^^,^^3^

Our findings have implications for brain cancer etiology. The “cell of origin” in glioma remains a highly controversial and debated topic. As astrocytes rarely divide in the healthy brain, they have been considered unlikely candidates.^65^^,^^66^ Our data challenge this view, as they suggest that mutations acquired during periods of active astrocyte proliferation, such as development or reactive astrogliosis, may lay silent in the healthy brain only to be activated by injury. In this model, dedifferentiated p53-deficient astrocyte-derived NSC-like cells that re-enter the cell cycle might then acquire additional mutations and initiate tumorigenesis. Indeed, seminal studies examining the role of p53 in SVZ neurogenesis demonstrated that, while p53 loss alone is not tumor-initiating, it does sensitize NSCs to mutagens and enables spontaneous acquisition of additional mutations, which ultimately lead to glioma formation.^19^^,^^67^^,^^68^^,^^69^^,^^70^ Importantly, the earliest detectable glioma cells in those models expressed the same molecular markers (Ascl1/Olig2) that we detected in AD-tdTomato^+^-NSCLs.^69^^,^^70^ To examine the possibility that astrocyte-derived *p53*^*−/−*^-NSCL cells may become prone to transformation as their *p53*^*−/−*^ NSC counterparts, we used soft-agar assays comparing *p53*^*−/−*^ and *p53*^*+/+*^ astrocytes cultured in BMP4 or EGF over time. We found that dedifferentiated *p53*^*−/−*^ NSCL cells in EGF acquired anchorage independence (a readout of tumorigenicity) after 9 passages, whereas *p53*^*−/−*^ astrocytes in BMP4 and *p53*^*+/+*^ astrocytes in either culture condition, did not (Figures S6G and S6H), providing proof of principle that p53-dependent dedifferentiation increases the probability of astrocyte transformation via a dedifferentiation step. Future studies should examine the *in vivo* tumorigenic potential of AD-tdTomato^+^-NSCL after longer-term proliferation and exposure to injury signals, for example, following repeated trauma or chronic inflammation.^71^

A link between brain injury and human glioma has long been proposed and is supported by some studies.^72^^,^^73^^,^^74^^,^^75^^,^^76^^,^^77^ To probe this more directly, we used electronic health records and propensity-score matching to estimate the impact of head injury on brain cancer in patients. We identified 23,232 patients who had a diagnosis of head injury and 194,942 matched controls using propensity-score matching, matched by year of birth, sex, and socioeconomic deprivation. The known glioma risk factors, i.e., phakomatoses and radiation exposure, were used as positive, whereas diabetes and fatty-liver disease were used as negative controls. As expected, the risk of developing brain cancer was the highest in patients with phakomatoses (hazard ratio [HR] 26.18, CI: 14.56–47.05, p < 0.0001), followed by those exposed to radiation (HR 6.36, CI: 5.15–7.85, p < 0.0001), whereas no association was observed with fatty-liver disease or diabetes (HR 0.83; CI: 0.64–1.07, p = 0.151). Strikingly, patients who experienced a head injury were also approximately 3.8 times more likely to develop a brain cancer later in life, which is indicative of injury being a significant risk factor for brain cancer (HR 3.78, CI: 2.85–5.02, p < 0.0001) (Figure S6I).

It is tempting to speculate that the mechanisms identified here may be relevant to glioblastoma recurrence, which in the majority of patients occurs within 2 cm of the resection cavity.^78^^,^^79^ The exposure of residual tumor cells to injury signals induced by surgery, chemotherapy, and radiotherapy in this region^12^^,^^80^^,^^81^ may convert residual p53-deficient tumor cells to a cancer-initiating, NSC-like state.

Finally, our observations have implications for age-related pathology. They suggest that the inflammation caused by injury in early life, even if relatively mild, has a long-term impact on tissue function and ultimately exacerbates age-dependent neuroinflammation in the periwound area.^82^^,^^83^^,^^84^ It is of note that p53 levels in healthy astrocytes increased upon aging. Future studies should explore whether this reduces astrocyte plasticity or injury responses with age.

In conclusion, our study shows that early-life injury primes mutated cells to dedifferentiation in later life by sensitizing them to age-dependent inflammatory cues and suggests that unraveling the complex interactions between injury and aging may unlock new strategies for cancer prevention.

## STAR★Methods

### Key resources table

**Table:** 

REAGENT or RESOURCE	SOURCE	IDENTIFIER
**Antibodies**		

goat polyclonal anti-Apolipoprotein E (ApoE)	Millipore	Cat#AB947; RRID: AB_2258475
rabbit polyclonal anti-Ascl1	Cosmo Bio	Cat#CAC-SK-T01-003; RRID: AB_10709354
rabbit anti-β-actin (D6A8)	Cell Signalling Technology	Cat#8547; RRID: AB_10950489
rabbit anti-cleaved caspase 3 (Asp175) (5A1E)	Cell Signalling Technology	Cat#9664; RRID: AB_2070042
rat monoclonal anti-CD68 (clone FA-11)	Abcam	Cat#ab53444; RRID: AB_869007
rabbit polyclonal anti-doublecortin (DCX)	Abcam	Cat#ab18723; RRID: AB_732011
rabbit polyclonal anti-GFAP	DAKO	Cat#Z0334; RRID: AB_10013382
mouse monoclonal anti-GFAP (clone GA5)	Millipore	Cat#MAB3402; RRID: AB_94844
rabbit polyclonal anti-Iba1	Wako	Cat#019-19741; RRID: AB_839504
rabbit monoclonal anti-Ki67 [SP6]	Abcam	Cat#ab16667; RRID: AB_302459
rabbit polyclonal anti-NFIA	Atlas antibodies	Cat#HPA006111; RRID: AB_1854422
mouse IgM anti-O4	R&D	Cat#MAB1326; RRID: AB_357617
rabbit polyclonal anti-Olig2	Millipore	Cat#AB9610; RRID: AB_570666
rabbit anti-Olig2 [EPR2673]	Abcam	Cat#ab109186; RRID: AB_10861310
rabbit polyclonal anti-p53	Leica	Cat#p53-CM5; RRID: AB_2895247
rabbit polyclonal anti-PCP4	Atlas antibodies	Cat#HPA005792; RRID: AB_1855086
goat polyclonal anti-PDGFRα	R&D	Cat#AF1062; RRID: AB_2236897
rabbit polyclonal anti-RFP	Antibodies-online	Cat#ABIN129578; RRID: AB_10781500
mouse anti-S6 Ribosomal Protein (54D2)	Cell Signalling Technology	Cat#2317; RRID: AB_2238583
rabbit anti-phospho-S6 ribosomal protein (S240/244)	Cell Signalling Technology	Cat#5364; RRID: AB_10694233
rabbit polyclonal anti-Sox2	Abcam	Cat#ab97959; RRID: AB_2341193
mouse monoclonal anti-Sox2 [9-9-3]	Abcam	Cat#ab79351; RRID: AB_10710406
rabbit anti-Tubulin3 (TUBB3) (Tuj1)	Biolegend	Cat#845501; RRID: AB_2566588
mouse anti-vimentin (clone V9)	Abcam	Cat#ab8069; RRID: AB_306239

**Bacterial and virus strains**		

Ad-CMV-Null	Vector Biolabs	Cat#1300
Ad-CMV-iCre	Vector Biolabs	Cat#1045
Ad-GFAP-Cre	University of Iowa Viral Vector Core	n/a

**Chemicals, peptides, and recombinant proteins**		

EdU	Santa Cruz	Cat#sc-284628
Tamoxifen	Sigma	Cat#T5648
Endoxifen	Tocris	Cat#3705
LPS (*E. coli* O55:B5)	Merck	Cat#L2637
Osimertinib	AKT Labs	Cat#A985134
EGF	Peprotech	Cat# 315-09-1000
Torin1	Generon	Cat# A8312
4OHT	Sigma	Cat#H7904
Trypsin	Sigma	Cat#T4799
Fetal bovine serum (FBS)	Sigma	Cat#F7524
Poly-L-lysine hydrobromide (PLL)	Sigma	Cat#P1274
BMP4	Peprotech	Cat#315-27
FGF	Peprotech	Cat#450-33A
AstroMACS Medium	Miltenyi	Cat#130-117-031
Low melting point agarose	Invitrogen	Cat#16520-100
AMPure XP beads	Beckman Coulter	Cat#A63881

**Critical commercial assays**		

Adult Brain Dissociation Kit	Miltenyi	Cat#130-107-677
Click-iT™ EdU Alexa Fluor™ 647 Imaging kit	Invitrogen	Cat#C10340
Click-iT™ Plus OPP Alexa Fluor™ 488 kit	Invitrogen	Cat#C10456
iScript gDNA clear cDNA synthesis kit	Bio-rad	Cat#1725034
10x Genomics Single Cell 3’ v2 kit	10X Genomics	Cat#PN-120237
Chromium Next GEM Single Cell 3' Kit v3.1	10X Genomics	Cat#1000269
EZ-Magna ChIP kit	Millipore	Cat#MAGNA0002
NEBNext Ultra II DNA library prep kit	NEB	Cat# E7645S

**Deposited data**		

Postnatal scRNA-seq data	This paper	GEO: GSE196408
Adult scRNA-seq data	This paper	GEO: GSE220477
*In vivo* scRNA-seq data	This paper	GEO: GSE220479
ChIP-seq data	This paper	GEO: GSE196863
Zamboni et al scRNA-seq dataset	Zamboni et al.^1^	GEO: GSE139842
Li et al RNA-seq dataset	Li et al.^20^	GEO: GSE147870
Guttenplan et al RNA-seq dataset	Guttenplan et al.^36^	GEO: GSE143598
Gyoneva et al RNA-seq dataset	Gyoneva et al.^37^	GEO: GSE131869

**Experimental models: Cell lines**		

Primary Human astrocytes	Sciencell	Cat#1800

**Experimental models: Organisms/strains**		

Mouse: GFAP-CreER: Tg(GFAP-cre/ERT2)1Fki	Hirrlinger et al.^23^	MGI:4418665
Mouse: Glast-CreERT2: Slc1a3^tm1(cre/ERT2)Mgoe^	Mori et al.^29^	MGI:3830051
Mouse: *p53*^*flox/flox*^: Trp53^tm1Brn^	Marino et al.^24^	MGI:1931011
Mouse: LSL-tdTomato: B6.Cg-Gt(ROSA)26Sor^tm14(CAG-tdTomato)Hze^/J	The Jackson Laboratory; Madisen et al.^25^	Cat#007914; RRID:IMSR_JAX:007914

**Oligonucleotides**		

See Table S1 for primer sequences		

**Recombinant DNA**		

Plasmid: shp53 pLKO.1	Godar et al.^85^	Addgene plasmid # 19119; RRID:Addgene_19119
Plasmid: pLKO.1 - TRC control	Moffat et al.^86^	Addgene plasmid # 10879; RRID:Addgene_10879

**Software and algorithms**		

CellRanger v3.1.0	10X Genomics	https://support.10xgenomics.com/single-cell-gene-expression/software
bcl2fastq v2.20.0	Illumina	https://emea.support.illumina.com/sequencing/sequencing_software/bcl2fastq-conversion-software.html
R v4.0.4	CRAN	https://www.r-project.org/
bayNorm v1.8.0	Tang et al.^87^	https://bioconductor.org/packages/bayNorm/
Seurat v4.0.4	Stuart et al.^88^	https://cloud.r-project.org/web/packages/Seurat/index.html
SPRING	Weinreb et al.^42^	https://kleintools.hms.harvard.edu/tools/spring.html
Nextflow nf-core/chipseq pipeline v1.0dev	Ewels et al.^89^	https://github.com/nf-core/chipseq; https://doi.org/10.5281/zenodo.3240506
Fiji Image J	Schindelin et al.^88^	https://imagej.nih.gov/ij/
Cellprofiler	McQuin et al.^90^	http://cellprofiler.org RRID:SCR_007358

**Other**		

Alzet Brain Infusion kit 3	Alzet	Cat#0008851
Alzet Osmotic minipump (model 1002)	Alzet	Cat#1002

### Resource availability

#### Lead contact

Further information and requests for resources and reagents should be directed to and will be fulfilled by the lead contact, Simona Parrinello (s.parrinello@ucl.ac.uk).

#### Materials availability

This study did not generate new unique reagents.

### Experimental model and subject details

#### Mice

All animal procedures were carried out in accordance with the Animal Scientific Procedures Act, 1986 and approved by the UCL Animal Welfare and Ethical Review Body (AWERB) in accordance with local ethical and care guidelines and the International guidelines of the Home Office (UK). In order to fate map and induce gene deletion in astrocytes, GFAP-CreER (Tg(GFAP-cre/ERT2)1Fki)^23^ or Glast-CreERT2 (Slc1a3^tm1(cre/ERT2)Mgoe^)^29^ were crossed to mice with conditional p53 deletion (*p53*^*flox/flox*^) (Trp53^tm1Brn^)^24^ or *p53*^*+/+*^ (*p53*^*wt/wt*^). A CAG-LoxP-STOP-LoxP tdTomato reporter in the Rosa26 locus (LSL-tdTomato) (B6.Cg-Gt(ROSA)26Sor^tm14(CAG-tdTomato)Hze^/J)^25^ was included in all crosses to trace recombined cells. All animals were in a mixed 129Sv x C57BL/6 background and both males and females were analysed. Approximately an equal number of male and female mice were used per experiment. Mice were group-housed (where possible) in individually ventilated cages and maintained with 12-hour light/dark cycles with water and chow available *ad libitum*.

#### Primary postnatal astrocyte isolation and culture

For isolation of primary cortical astrocytes, litters of mice of indicated genotypes aged postnatal day (P3) were sacrificed by decapitation. For experiments with GFAP-CreER astrocytes, brains were processed separately and combined at first passage after genotyping for GFAP-CreER allele. For other experiments, brains were pooled prior to digestion. Digestion protocol was based on.^91^ Briefly, the cortices were dissected in HEPES-buffered HBSS, meninges removed, and cut into small pieces before digestion in 0.25 % trypsin (Sigma, T4799) in HBSS for 30 min at 37°C followed by centrifugation (5 min, 300xg). Tissue pieces were dissociated by trituration in astrocyte expansion media (DMEM-Glutamax, 10% FBS (Sigma, F7524), 100 μg/ml kanamycin, 2 μg/ml gentamicin) and plated onto poly-L-lysine hydrobromide (PLL, 1:2500, Sigma P1274) coated plates. Cells were cultured at 37 °C, 5 % CO_2_. Debris was removed the following day by washing in PBS and after 6-7 days in culture microglia and macrophages, oligodendrocytes and OPCs and neurons were removed by immunopanning using CD45, O4 and L1 antibodies as described in ^38^. Recovered astrocytes were plated in astrocyte expansion media. Once confluency was reached astrocytes were passaged and plated at a density of 20x10^3^ cells/cm^2^ and cultured until induction of recombination in astrocyte maturation media (50% Neurobasal, 50% DMEM, 1mM sodium pyruvate, 292μg/ml L-glutamine, 1X SATO, 5μg/ml M-acetylcysteine, 100μg/ml kanamycin, 2μg/ml gentamicin,^38^ supplemented with 25ng/ml BMP4 (Peprotech)).

#### Primary adult astrocyte isolation and culture

For isolation of primary cortical astrocytes from adult mice, cortices from two 8–12 week old GFAP-CreERT2;*p53*^*flox/flox*^;LSL-tdTomato mice were pooled and dissociated using the Adult Brain Dissociation Kit (Miltenyi 130-107-677), according to manufacturer’s instructions. Astrocytes were purified from cell suspension after removal of debris and red blood cells with ACSA-2 MicroBeads (Miltenyi 130-097-678) according to manufacturer’s instructions and plated onto two wells of a PLL and laminin coated 12 well plate in AstroMACS Medium (# 130-117-031). The following day, plates were gently washed with media to remove debris. Media was replaced every 3 days thereafter. While in culture, adult astrocytes showed very limited divisions, as previously described.^92^

#### Human astrocyte culture

Primary human astrocytes (Sciencell #1800) were subcultured according to manufacturer’s instructions. Alternatively, astrocytes were prepared by differentiation of primary human fetal neural stem cells (BRC2351, BRC2389 and BRC2404) by incubation in “basal media” (DMEM-F12, N2 (1/200), B27 (1/100), 5mM HEPES, 1x MEM NEAA. 50μM betamercaptoethanol, 0.012% BSA, 6.525mg/ml glucose, 100μg/ml kanamycin, 2μg/ml gentamicin)^93^ supplemented with 10% FBS for 14 days.

### Method details

#### *In vivo* administrations and surgeries

For localised intracortical astrocyte recombination, 2-3-month-old mice underwent stereotaxic injection of 1.807μl of endoxifen (13.3μM Tocris 3705/10) (anteroposterior -1.2, mediolateral 1.7, dorsoventral -1 relative to bregma) under isofluorane anaesthesia. To induce recombination in astrocytes in an injury-free setting, 2-3-month-old mice were injected once daily for 5 days intraperitoneally (i.p.) with 100mg/kg tamoxifen (Sigma, T5648 prepared in 10% ethanol, 90% corn oil). For lipopolysaccharide (LPS) experiments, mice were injected with LPS (*E. coli* O55:B5, Merck L2637, dissolved in PBS) 1mg/kg i.p. 11 days after endoxifen injection and sacrificed 3 days later. To investigate the response of injured cortical astrocytes in the absence of EGFR signalling the EGFR inhibitor osimertinib was used. 2-4-month-old mice were injected i.p. with 15 mg/kg osimertinib (A985134, AKT Labs, in 10% DMSO/90% corn oil) or vehicle control. Injections were started 2 days prior to intracortical endoxifen injection (as above) and on alternate days for the following 2 weeks when animals were sacrificed. For EGF infusion experiments, a brain infusion cannula (Alzet Brain Infusion kit 3 adjusted for cortical targeting using 4 spacers) attached to an overnight pre-equilibrated mini-osmotic pump (Alzet, 1002) filled with EGF (Peprotech 60μg/ml in 0.9% saline;1% BSA) or vehicle control was inserted into the cortex following endoxifen injection. The mini-osmotic pump was inserted into a subcutaneous pocket according to the manufacturer’s instructions.

#### p53 recombination in primary astrocytes

For adenoviral experiments, allele recombination was induced by overnight adenoviral infection with Ad-CMV-Null, Ad-CMV-iCre or Ad-GFAP-Cre) at multiplicity of infection of 30. Following infection, astrocytes were cultured in “basal media” (DMEM-F12, N2 (1/200), B27 (1/100), 5mM HEPES, 1x MEM NEAA. 50μM betamercaptoethanol, 0.012% BSA, 6.525mg/ml glucose, 100μg/ml kanamycin, 2μg/ml gentamicin)^93^ supplemented with 20ng/mL EGF and 10ng/mL FGF-2 (Peprotech) or 25ng/ml BMP4. For 4-hydroxytamoxifen (4-OHT) experiments, after 48h in astrocyte maturation media, media was changed to “basal media” supplemented with EGF/FGF or BMP4 as above with 4OHT (200nM, Sigma H7904) or vehicle control (ethanol). For experiments involving mTOR inhibitor, Torin, cells were incubated with Torin1 (250nM in DMSO, Generon A8312,) or DMSO vehicle control.

For primary adult astrocytes, after 7 days in culture, media was changed to astrocyte maturation media, as described above, for 2 days. Recombination was induced, as described above, by changing media to “basal media” supplemented with EGF and FGF and 4OHT (Sigma H7904, 200nM). Astrocytes were incubated for 5 days before collection for scRNA-seq as described below.

For human astrocytes, p53 downregulation was induced by lentiviral infection with shp53 or vector control. shp53 pLKO.1 puro was a gift from Bob Weinberg (Addgene plasmid # 19119; http://n2t.net/addgene:19119; RRID:Addgene_19119).^85^ pLKO.1 - TRC control was a gift from David Root (Addgene plasmid # 10879; http://n2t.net/addgene:10879; RRID:Addgene_10879).^86^ Lentivirus was produced by co-transfection of above plasmids with VSVG and Delta8.9 into HEK293T cells using polyethylenimine, followed by viral concentration by centrifugation (3 hrs at 50,000xg at 4°C). After lentiviral transduction, astrocytes were incubated for 7 days in “basal media” supplemented with EGF and FGF or BMP4 as above, before subjecting to clonogenic or neurosphere assays or re-differentiation experiments.

#### Clonogenic assays

For clonogenic assays, cells were fixed (4% PFA for 15 minutes) and stained with crystal violet (0.2% in 10% ethanol) 7 days after recombination. Excess crystal violet was washed off and plates were fully dried, before scanning on a standard scanner at 1000 dpi. The number of colonies were scored manually in ImageJ.

#### Neurosphere assay and re-differentiation

After 7 days in basal media containing EGF and FGF, cells were passaged and plated for neurosphere assays. To assess self-renewal, 10,000 cells were plated in 7ml neurosphere media (DMEM-F12, 1x B27 without Vitamin A, 4μg/ml heparin, 10ng/ml FGF, 20ng/ml EGF, 100μg/ml kanamycin, 2μg/ml gentamicin) in uncoated T25 flasks. After 7 days, neurospheres were scored, cells passaged as described in Belenguer et al.,^94^ and replated as described above.

For assessment of multi-lineage differentiation, 50,000 cells were seeded onto PLL and laminin-coated glass coverslips in “basal media” supplemented with EGF and FGF. The following day, media was replaced with basal media supplemented with FGF only. After 48 hours, media was replaced with “basal media” in the absence of additional growth factors and cells were incubated for a further 48 hours before fixation.

#### Astrocyte soft agar experiments

Primary *p53*^*flox/flox*^ astrocytes were recombined with adenovirus (Ad-Null or Ad-GFAP-Cre) and incubated in media supplemented with EGF+FGF or BMP4 as described above. Upon reaching confluency, *p53*^*-/-*^ EGF/FGF cells were passaged with accutase and replated onto laminin-coated dishes. Cells from other conditions were non-dividing, so did not reach confluency, but were periodically passaged to smaller wells. Anchorage-independence (a read out of tumorigenicity) was monitored roughly every 3 passages by soft agar assay. 3333 cells were plated per well in the top layer of 0.33% low melt agarose (Invitrogen) over bottom layer of 0.5% low melt agarose in a 6 well plate. Dedifferentiation basal media supplemented with EGF+FGF was overlayed to prevent drying and half media was changed every 3-4 days. After 12 days, plates were fixed and stained with crystal violet as described above. Soft agar colonies were observed after passage 9 in the *p53*^*-/-*^ EGF/FGF condition, and not in the others.

#### Immunofluorescence and Immunohistochemistry

For immunohistochemical analysis, mice were sacrificed at indicated time points by transcardial perfusion with PBS followed by 4% paraformaldehyde (PFA) under terminal anaesthesia and the brain post-fixed in 4% PFA at 4°C overnight. To assess cell proliferation, EdU (50mg/kg) was administered by i.p. injection 6 hours prior to collection. 50 μm vibratome sections or 35 μm floating cryosections were permeabilised and blocked in 1% Triton X-100, 10% donkey serum, PBS for 1.5hrs then incubated overnight at 4°C with primary antibodies diluted in 0.1% Triton X-100, 10% serum, PBS. The following day, sections were washed in 0.1 % Triton X-100, PBS then incubated with secondary antibodies and DAPI in 0.1% Triton X-100, 10% serum, PBS for 1 hour at RT. Sections were washed and mounted in ProlongGold Antifade mountant.

For *in vitro* immunofluorescence, cells were incubated for 7 days as described, and fixed for 15 min in 4% PFA, permeabilised in 0.5% Triton X-100 for 20 min and blocked in 10% serum in PBS for 30 min before incubation with primary antibody overnight at 4°C in 10% serum, PBS. Coverslips were incubated with secondary antibodies and DAPI in 10% serum, PBS at RT for 1h, before mounting in ProlongGold. For experiments involving assessment of cell proliferation, cells were incubated with 10μM EdU for 2 hours prior to fixation. For experiments involving assessment of protein synthesis, cells were incubated with 20μM O-propargyl-puromycin (OP-Puro) for 30 mins prior to fixation. Click-iT EdU or OP-Puro (Invitrogen C10086 and C10458 respectively) labelling was carried out according to manufacturer’s instructions. For phalloidin staining, cells were stained with Alexa Fluor 647 phalloidin (Invitrogen, A22287) according to manufacturer’s instructions.

Primary antibodies used were goat anti-ApoE (1:1000, Millipore AB947), mouse anti-Ascl1 (1:200, gift from Francois Guillemot), rabbit anti-Ascl1 (1:500, Cosmo Bio CAC-SK-T01-003), rabbit anti-β-actin (1:200, CST 8457), rabbit anti-cleaved caspase 3 (1:500, CST 9664), rat anti-CD68 (1:500, Abcam ab53444), rabbit anti-DCX (1:1000, Abcam ab18723), rabbit anti-GFAP (1:1000, DAKO Z0334), mouse anti-GFAP (1:1000, Millipore MAB3402), rabbit anti-Iba1 (1:2000, Wako 019-19741), rabbit anti-Ki67 (1:500, Abcam ab16667), rabbit anti-NFIA (1:1000, Atlas antibodies HPA006111), mouse IgM anti-O4 (1:500 on live cells 20 min before fixation, R&D MAB1326), rabbit anti-Olig2 (1:500 *in vivo*, 1:1000 *in vitro*, Millipore AB9610), rabbit anti-Olig2 (1:500 human *in vitro*, Abcam ab109186), rabbit anti-p53 (1:500, Leica p53-CM5), rabbit anti-PCP4 (1:500, Atlas antibodies HPA005792), goat anti-PDGFRα (1:500, R&D AF1062), rabbit anti-RFP (1:400, Antibodies-online ABIN129578), mouse anti-S6 ribosomal protein (1:500, CST 2317) rabbit anti-phospho-S6 ribosomal protein (S240/244) (1:500, CST 5364), rabbit anti-Sox2 (1:400, Abcam ab97959), mouse anti-Sox2 (1:400, Abcam ab79351), rabbit anti-Tuj1 (1:500, Biolegend 845501), mouse anti-vimentin (1:500, Abcam ab8069). Alexa Fluor conjugated secondary antibodies were obtained from ThermoFisher.

#### Image analysis and processing

Quantifications were carried out in Fiji ImageJ.^95^ For quantification of tdTomato morphology, a minimum of 200 tdTomato^+^ cells were scored for rounded/astrocytic morphology over at least 3 sections per mouse. In injured mice, the sections chosen all had evidence of injury (dip in tissue or inflammation).

For quantification of distance of cells from wound site used in Figures 1F and 2G, tdTomato^+^ cells were scored as astrocytic or AD-tdTomato, and the perpendicular distance was measured in ImageJ to the top of injection dip or to the injection tract, whichever was shortest. For Figures 1F, >100 cells were measured over n=3 *p53*^*Gfap-icKO*^ animals. For Figure 2G, >200 cells were measured over n=5 *p53*^*Gfap-icKO*^ animals.

For skeleton analysis of microglia in Figure S3C, maximum intensity projection (MIP) of confocal images of Iba1 staining were processed by despeckling, followed by binarization using the Auto Local Threshold function in ImageJ with method=Phansalkar. Images were then processed with Skeletonize followed by Analyze Skeleton. As a measure of microglia arborisation, number of junctions were normalised to the number of microglia in the field of view. For CD68 intensity analysis in Figure S3D, MIPs were despeckled and used to generate a mask of CD68 particles using the Auto Local Threshold function in ImageJ with method=Phansalkar. Analyze Particles function was then redirected to the original MIP to measure the Integrated density (IntDen) of particles >2 pixels in size. Average particle IntDen per field of view was calculated in R before normalisation to the average of uninjured young animals.

For single cell analysis of Ascl1, Sox2, Olig2 or OP-Puro intensity, a minimum of 10 fields of view were used per condition per replicate. Images were split into individual channels using ImageJ. Intensity analysis was performed using CellProfiler,^90^ using a custom pipeline. Briefly, nuclei were detected and used to generate the masks. An image was generated of the masks, which was checked for accuracy and the image/data discarded if segregation was unsuccessful. Nuclei overlapping the image border were discarded. Where applicable, tdTomato intensity was measured, and nuclei filtered for positivity. MGV was measured for the nuclear mask within each cell. Downstream analysis was performed in R: for each replicate, values were normalised to the average of the *p53*^*+/+*^ EGF/FGF condition, before random downsampling data to lowest condition and combining replicates. Plots were generated in Graphpad Prism, whereby each point indicates a single cell.

For analysis of intensity of phospho-S6RP, images were taken of random fields of view using consistent exposure/laser settings. Images were analysed in ImageJ to generate a mask of the cytoplasm by thresholding tdTomato or pS6RP channel and DAPI to binary masks and subtracting the nuclear mask from the tdTomato mask. This mask was used to measure the pixel mean gray value (MGV) of pS6RP which was normalised to MGV of S6RP.

#### scRNA-sequencing of *in vitro* cells

After 5 days of incubation of GFAP-CreERT2;*p53*^*flox/flox*^;LSL-tdTomato primary postnatal or adult astrocytes in media supplemented with EGF and FGF and 4OHT, cells were harvested by trypsinisation. Cells were washed and filtered through a 30μm MACS SmartStrainer (Miltenyi), as described in the 10X Genomics manual. Cells were counted with a haemocytometer using trypan blue for exclusion of dead cells. The volume was adjusted to cell concentration of 700-1200 cells/μl, with >80% viability. For the postnatal astrocytes, two replicates were performed from two independent astrocyte preparations, A19 and A26, processed on different days. Gel Bead-In-EMulsions (GEMs) and library preparation was performed as per the 10x Genomics Single Cell 3’ v2 reagent kit protocol. The two libraries from postnatal astrocytes preps were sequenced on a single lane of Illumina HiSeq 2500 (Paired End 2 x 100bp) and de-multiplexed and analysed using CellRanger v3.1.0 and bcl2fastq v2.20.0.

For the adult *in vitro* astrocytes experiment, library preparation from single cells was performed as per the 10X Genomics Chromium Next GEM Single Cell 3' Kit v3.1 and sequenced on an Illumina Novaseq 6000 Instrument (Paired End, 2x 150bp).

#### Acute purification of tdTomato^+^ cells from aged injured mice and Smartseq3 scRNA-seq

Approximately 1 year after endoxifen intracortical injection, mice were sacrificed by cervical dislocation and the area around the injection site containing tdTomato^+^ cells was dissected under fluorescent guidance. As a negative control for tdTomato gating, the cortex of one uninjured aged mouse was used. Tissue was dissociated using the Adult brain dissociation kit (Miltenyi (130-107-677) using the protocol for <100mg tissue. To obtain sufficient injured material, we pooled mice of the same genotype (2 *p53*^*Gfap-wt/wt*^ and 3 *p53*^*Gfap-icKO*^). Following dissociation and debris removal, cells were sorted on a BD FACSAria III for tdTomato+ and DAPI- for live/dead discrimination. Single cells were sorted into individual wells of 384 well plates containing 3μl Smart-seq3 lysis buffer (for exact components, please refer to Hagemann-Jensen et al.^96^). Even after pooling, brains yielded small number of tdTomato+ cells from FACS (301 *p53*^*Gfap-wt/wt*^ and 382 *p53*^*Gfap-icKO*^), consistent with small region targeted by endoxifen injection. Plates were vortexed, spun down at 350rcf and frozen immediately at -80°C.

Prior to reverse transcription, plates were thawed on ice and incubated at 72°C for 3 min. 1μl of reverse transcription mix (for exact components please refer to Hagemann-Jensen et al.^96^) was added to each well using Echo 525 acoustic liquid handler, plate was mixed and reaction was carried out at 42°C for 90 min followed by 10 cycles of 2min at 50°C and 2min at 42°C. Reaction was terminated by incubation at 85°C for 5 mins. For pre-amplification step 6μl of PCR mix containing 1X KAPA HiFi HotStart ReadyMix, water, 0.5μM PCR forward primer and 0.1μM PCR reverse primer (for exact sequences please refer to Hagemann-Jensen et al.^96^) was added to each well using Echo 525 acoustic liquid handler, plates were vortexed and PCR performed as follows: 3 min at 98°C for initial denaturation, 24 cycles of 20 s at 98°C, 30 s at 65°C and 6 min at 72°C. Final elongation was performed for 5 min at 72°C. cDNA samples were size selected using 0.6x AMPure XP beads (A63881; Beckman Coulter), concentration was measured with Varioscan Lux (ThermoFisher) in a 384-well plate using Qubit Fluorometer solution and quality was checked on the 5200 Fragment Analyzer System (Agilent). Samples displaying satisfactory amplification traces between 300 and 9000 bp were selected for tagmentation (125 *p53*^*Gfap-wt/wt*^ and 145 *p53*^*Gfap-icKO*^ cells).

Sequencing libraries were made using Nextera XT DNA Library Preparation Kit (Illumina) according to manufacturer’s instructions at a 20x reduced scale and using 25pg of cDNA. All reagents were distributed using Echo 525 acoustic liquid handler. After tagmentation, equal volumes of each sample were pooled and the pool was purified with 0.6x AMPure XP beads (Beckman Coulter). Concentration of the final pool was measured on Qubit 2.0 and library size distribution was checked on a high-sensitivity DNA chip (Agilent Bioanalyzer). Pool was loaded on a MiSeq Reagent Micro Kit v2 (300 cycles) at a 12pM final concentration and re-pooled based on mapping rates (123 *p53*^*Gfap-wt/wt*^ and 141 *p53*^*Gfap-icKO*^ cells). Final re-pooled libraries were sequenced, 150-bp paired end, on a high-output flow cell (Illumina 20024907) using an Illumina NextSeq500 instrument.

#### Preprocessing and normalization of scRNA-seq data

##### Cells and genes filtering (postnatal *in vitro* astrocytes)

We generated data for 12040 cells. High-quality cells were selected according to the following two criteria: 1) Number of detected genes ranging between 200 and 5000; 2) Proportion of mitochondria genes below 0.15 and 0.1 for batches A19 and A26 respectively; this led to 11635 high-quality cells (Figure 5B). Cells were further filtered based on tdTomato expression leading to 8699 cells with tdTomato counts above 0. In term of features, 13 mitochondrial genes were filtered out and genes with non-zero count in at least 3 cells across both batches were kept for downstream analysis. As a result, 17046 genes were included in this study. The two batches were combined before further normalisation and imputation.

##### Cells and genes filtering (adult *in vitro* astrocytes)

High-quality cells were selected according to the following criteria: tdTomato total counts > 0, proportion of mitochondrial genes < 0.15, and log_2_ total counts between 14 and 17.48. After filtering, 523 out of 1562 adult astrocytes were kept for further analysis. Genes with non-zero counts in at least 10% of all cells were kept.

##### Cells and genes filtering (adult *in vivo* astrocytes)

High-quality cells were selected according to the following criteria: tdTomato total counts > 0, which resulted in 99 *p53*^*Gfap-wt/wt*^ and 108 *p53*^*Gfap-icKO*^ cells. Genes with zero count in all cells were removed.

##### Normalisation and imputation (postnatal and adult *in vitro* astrocytes)

For normalisation and imputation, the bayNorm package with mean_version = TRUE was applied.^87^ The bayNorm normalized counts were then imported into the Seurat package^88^ for dimension reduction and clustering analysis.

##### Identification of cluster marker genes (postnatal *in vitro* astrocytes)

Clustering was performed in Seurat using the Louvain approach in PCA space on bayNorm normalised counts of the 2000 most variable genes. RaceID^97^ was then applied to narrow down the most variable genes to 1000 genes. Marker genes in each cluster were identified using this set of 1000 genes by comparing cells in each one of the 9 clusters with all other cells using MAST.^98^ Gene lists and signatures were from the GSEA website.^99^^,^^100^

##### Cell clustering (adult *in vitro* and *in vivo* astrocytes)

Clustering of adult astrocytes in Figure S5Kiii was performed in Seurat using the Louvain approach in PCA space on bayNorm normalised counts. Clustering of *in vivo* astrocytes in Figure S5Kiv was performed in Seurat on raw counts using the Louvain approach in PCA space.

##### RNA velocity and pseudo-time analysis (postnatal and adult *in vitro* astrocytes)

Low-dimension coordinates from t-SNE together with cluster labels annotated by Seurat were used as inputs for RNA velocity inference with the Velocyto and scvelo packages.^101^^,^^102^ Monocle3^103^ was used for pseudo-time and SPRING^42^ for graph analyses. Note that in SPRING, the input data were raw counts since SPRING has its own preprocessing framework. Postnatal clusters (Cl1-Cl6) were ordered according to SPRING second dimension (Figure 5D), adult astrocyte clusters (A0-A5) according to diffusion map^104^ (DC1 in Figure S5L).

For pathway analysis in Figures 5E, S5M, and S6C HALLMARK_P53_PATHWAY, GO_CYTOSKELETON, GO_RIBONUCLEOPROTEIN_COMPLEX_BIOGENESIS, GO_CELL_CYCLE, LEIN_ASTROCYTE_MARKERS, HALLMARK_PI3K_AKT_MTOR_SIGNALING, HALLMARK_MYC_TARGETS_V1 and HALLMARK_MYC_TARGETS_V2 were used (named “p53 pathway”, “Actin remodelling”, “Ribosomal biogenesis”, “Cell cycle”, “Astrocyte markers”, “Pi3K Akt mTOR signalling”, “Myc targets v1” and “Myc targets v2” in the title of panels, respectively). HALLMARK and GO genesets were downloaded from MsigDB (msigdbr R package V7.5.1).^99^^,^^100^

##### Harmony integration of postnatal, adult and *in vivo* astrocytes

In Figure S5K, postnatal, adult and *in vivo* astrocytes datasets were integrated using the Harmony batch correction algorithm.^46^ SCT transform was applied to each dataset independently,^105^ then PCA analysis was performed on the combined data. Harmony was applied to the PCA first 20 principal components and a UMAP was obtained based on harmony low dimension space.

#### ChIP-sequencing and analysis

For ChIP-sequencing experiment, approx 2x10^7^ wild-type astrocytes were incubated for 5 days in basal media supplemented with 25ng/ml BMP4. ChIP was performed using the EZ-Magna ChIP kit (Millipore), according to manufacturer’s instructions, with the following modifications: sonication was performed with Bioruptor Pico with 10-20 cycles (30s on, 30s off at 4°C) until chromatin was average of 100 – 400bp. IP was performed overnight at 4°C with p53-CM5 antibody (Rabbit, Leica, 1:50) or 1mg Normal Rabbit IgG (Santa Cruz). Libraries were prepared with NEBNext Ultra II DNA library prep kit per manufacturer’s instructions with 13 cycles of PCR amplification. Libraries were quantified by Qubit and size distribution assessed by Agilent Bioanalyzer High Sensitivity DNA chip. Indexed libraries were pooled and sequenced on Illumina NextSeq 500 platform with NextSeq 500/550 Mid Output Kit v2.5 (150 Cycles) with Paired End 2x75 bp. Sequencing data was analysed using the nf-core/chipseq Nextflow pipeline.^89^ Briefly, QC was performed on Fastq files with FastQC before adapter trimming with TrimGalore. Reads were aligned to the mouse genome GRCm38 and blacklisted regions filtered with BWA. Duplicate reads were removed with Picard, bigwigs generated, and peaks called with MACS2 NarrowPeak setting. Downstream peak analysis was performed in R with the Chippeakanno package^106^ and data was visualised with IGV or IgvR R package.

#### Polysome fractionation

Polysome fractionation was performed as previously described.^107^^,^^108^ Briefly, approx. 2x10^7^ primary *p53*^*flox/flox*^ astrocytes were infected with adenoviral Ad-Null or Ad-iCre virus and incubated for 5 days in basal media supplemented with EGF and FGF, and Torin (250nM) or DMSO control. Cells were lysed in ice cold gradient buffer (15mM Tris-HCl (pH 7.5), 0.3M NaCl, 15mM MgCl_2_, 0.1mg/ml cycloheximide, 1mg/ml heparin) supplemented with 1% Triton X-100 and 500U/ml recombinant RNase inhibitors. Samples were centrifuged after 2 minutes of lysis, and a small aliquot of supernatant taken as “Input” sample as in Figure S6F, the remainder of the supernatant was layered over a 10-50% sucrose gradient in gradient buffer. Samples were ultracentrifuged at 38000rpm on a SW40Ti rotor (Beckman) for 2 hours at 4°C. Eleven 1ml fractions were collected into 3ml of 7.7M guanidine-HCl and RNA precipitated and purified as in Johannes and Sarnow^108^ with additional heparin removal with LiCl. RNA profile was assessed with Agilent Tapestation High Sensitivity RNA ScreenTape. RNA was reverse transcribed to cDNA using Superscript IV (Invitrogen) according to manufacturer’s protocol, before quantitative PCR analysis as described below.

#### RNA isolation and qRT-PCR

For analysis of astrocyte Trp53 expression in Figure S3A, astrocytes were purified from cortices of 2-3 month, or 1 year, old mice using adult brain dissociation kit followed by ACSA-2 purification as described above for Primary adult astrocyte isolation. RNA was extracted from acutely-purified cell suspension using RNeasy Plus Micro kit according to manufacturer’s instructions. RNA was amplified, and cDNA libraries produced using the Smartseq2 method^109^ (protocol steps 1-27 with 17 PCR cycles at step 14). Resulting cDNA libraries were diluted to 1ng/μl before qRT-PCR analysis.

For analysis of tissue from the cortical injection site as in Figure S3I, mice were injected stereotaxically as described above. Seven days later, mice were culled by cervical dislocation, and a small area around the injection site was dissected. As a control, a similar size piece was taken from the contralateral cortex. For analysis of young/aged cortical tissue in Figure S3J, 3 month or 1 year old mice were culled by cervical dislocation and a small piece of cortical tissue from the same region as above was taken. For each experiment phenol/chloroform RNA extraction was performed, followed by reverse transcription of RNA to cDNA using iScript gDNA clear cDNA synthesis kit (BioRad) according to manufacturer’s instructions. cDNA was diluted at least 1:1 in water before qPCR was performed with qPCRBIO SyGreen Blue Mix Lo-ROX (PCR Biosystems). *Gusb* and *Ppia* were used as housekeeping genes for astrocyte and unpurified tissue lysate studies, respectively. The sequences of primers used in this study are listed in Table S1. Housekeeping-normalised Ct values for EGFR ligand genes (*Areg*, *Btc*, *Egf*, *Epgn*, *Ereg*, *Hbegf*, and *Tgfa*^110^) for the test group (“Ipsi” and “aged” on Figures S3I and S3J respectively) were normalised to the mean value from the reference groups (“contra” and “young” on Figures S3I and S3J respectively). All the genes and replicates were then pooled and displayed as boxplots.

#### Re-analysis of published RNA-seq datasets

For pathway analysis in Figures S1A and S1B, HALLMARK_P53_PATHWAY was used. HALLMARK genesets were downloaded from MsigDB (msigdbr R package V7.5.1).^99^^,^^100^ In Figure S1A, bayNorm with setting mean_version=TRUE was applied to single cells from the Zamboni scRNA-seq dataset^1^ and normalized counts were plotted. In Figure S1B, RPKM values provided in Li et al.^20^ were used. For Figures S3K and S3L (Gyoneva et al.^37^ and Guttenplan et al.^36^), read counts for each EGFR ligand gene (*Areg*, *Btc*, *Egf*, *Epgn*, *Ereg*, *Hbegf*, and *Tgfa*^110^) in all conditions were divided by the mean read counts of the reference groups (“Control” and “Young” on Figures S3K and S3L respectively).

#### Estimating the impact of head injury and additional risk factors on brain cancer outcomes using electronic health records

Electronic health records (EHRs) from secondary care with linkage to patient-level Index of Multiple Deprivation (i.e., an area-based indicator of socioeconomic deprivation) were utilised. Information governance approval was obtained from the Medicines and Healthcare products Regulatory Agency (19222). We considered these three diagnostic categories as exposures: (i) head injury, (ii) phakomatoses (i.e., neurocutaneous syndromes) and (iii) radiation exposure. Diagnoses were recorded in ICD-10 (see Table S2 for code list). To estimate the risk of brain cancer in patients with head injury, phakomatoses or in those who were exposed to radiation, we excluded patients who had a history of brain cancer occurring before the diagnosis of the aforementioned conditions. Controls were identified using propensity score matching, matched by year of birth, sex and socioeconomic deprivation. We performed optimal pair matching using the R matchit package. Cases and matched controls were followed from the date of diagnosis of head injury, phakomatoses or radiation exposure (matched dates were used for controls) until the first record of brain tumour, date of death or date of deregistration, whichever occurred first. For each of the three diagnostic categories, Cox proportional hazard regression models were fitted to estimate the hazard ratios and 95% confidence intervals (CIs) for the risk of brain cancer. To address potential biases, we evaluated the association between head injury and two negative outcome controls (diabetes and fatty liver disease) as there are no plausible mechanisms that head injury would affect the risk of diabetes or fatty liver disease. We evaluated the proportional hazards assumption using the Schoenfeld residuals. Analyses were performed using R (3.6.3) with these packages: data.table, matchit, tidyverse and survival.

### Quantification and statistical analysis

Statistical analysis was performed using GraphPad Prism 9 or R. All data are expressed as mean±SEM, unless otherwise stated. Significance is stated as follows: p>0.05 (ns), p<0.05 (^∗^), p<0.01 (^∗∗^), p<0.001 (^∗∗∗^), p<0.001 (^∗∗∗∗^). Significance was calculated as indicated in the figure legends. 1- or 2-tailed Student’s t test, Wilcoxon test were used for statistical comparisons between two groups. Two-way ANOVA with Tukey’s multiple comparisons was used to determine statistical significance of data with two grouping variables. No statistical method was used to predetermine sample size. Sample size was determined based on existing literature and our previous experience.

## Data Availability

• The scRNA-seq and ChIP-seq data have been deposited at GEO and are publicly available as of the date of publication. Accession numbers are listed in the key resources table. • This paper does not report original code. • Any additional information required to reanalyse the data reported in this paper is available from the lead contact upon request.

## Supplemental information

Document S1. Figures S1–S6 and Tables S1 and S2

Document S2. Article plus supplemental information

## References

[1] Zamboni M., Llorens-Bobadilla E., Magnusson J.P., Frisén J. (2020). A widespread neurogenic potential of neocortical astrocytes is induced by injury. Cell Stem Cell.

[2] Magnusson J.P., Göritz C., Tatarishvili J., Dias D.O., Smith E.M.K., Lindvall O., Kokaia Z., Frisén J. (2014). A latent neurogenic program in astrocytes regulated by Notch signaling in the mouse. Science.

[3] Magnusson J.P., Zamboni M., Santopolo G., Mold J.E., Barrientos-Somarribas M., Talavera-Lopez C., Andersson B., Frisén J. (2020). Activation of a neural stem cell transcriptional program in parenchymal astrocytes. eLife.

[4] Nato G., Caramello A., Trova S., Avataneo V., Rolando C., Taylor V., Buffo A., Peretto P., Luzzati F. (2015). Striatal astrocytes produce neuroblasts in an excitotoxic model of Huntington's disease. Development.

[5] Buffo A., Rite I., Tripathi P., Lepier A., Colak D., Horn A.P., Mori T., Götz M. (2008). Origin and progeny of reactive gliosis: A source of multipotent cells in the injured brain. Proc. Natl. Acad. Sci. USA.

[6] Sirko S., Behrendt G., Johansson P.A., Tripathi P., Costa M., Bek S., Heinrich C., Tiedt S., Colak D., Dichgans M. (2013). Reactive glia in the injured brain acquire stem cell properties in response to sonic hedgehog. [corrected]. Cell Stem Cell.

[7] Friedmann-Morvinski D., Bushong E.A., Ke E., Soda Y., Marumoto T., Singer O., Ellisman M.H., Verma I.M. (2012). Dedifferentiation of neurons and astrocytes by oncogenes can induce gliomas in mice. Science.

[8] Neftel C., Laffy J., Filbin M.G., Hara T., Shore M.E., Rahme G.J., Richman A.R., Silverbush D., Shaw M.L., Hebert C.M. (2019). An integrative model of cellular states, plasticity, and genetics for glioblastoma. Cell.

[9] Carén H., Stricker S.H., Bulstrode H., Gagrica S., Johnstone E., Bartlett T.E., Feber A., Wilson G., Teschendorff A.E., Bertone P. (2015). Glioblastoma stem cells respond to differentiation cues but fail to undergo commitment and terminal cell-cycle arrest. Stem Cell Rep..

[10] Brooks L.J., Clements M.P., Burden J.J., Kocher D., Richards L., Devesa S.C., Zakka L., Woodberry M., Ellis M., Jaunmuktane Z. (2021). The white matter is a pro-differentiative niche for glioblastoma. Nat. Commun..

[11] Richards L.M., Whitley O.K.N., MacLeod G., Cavalli F.M.G., Coutinho F.J., Jaramillo J.E., Svergun N., Riverin M., Croucher D.C., Kushida M. (2021). Gradient of Developmental and Injury Response transcriptional states defines functional vulnerabilities underpinning glioblastoma heterogeneity. Nat. Cancer.

[12] Brooks L.J., Simpson Ragdale H., Hill C.S., Clements M., Parrinello S. (2022). Injury programs shape glioblastoma. Trends Neurosci..

[13] Hong H., Takahashi K., Ichisaka T., Aoi T., Kanagawa O., Nakagawa M., Okita K., Yamanaka S. (2009). Suppression of induced pluripotent stem cell generation by the p53–p21 pathway. Nature.

[14] Lin T., Lin Y. (2017). p53 switches off pluripotency on differentiation. Stem Cell Res. Ther..

[15] Kawamura T., Suzuki J., Wang Y.V., Menendez S., Morera L.B., Raya A., Wahl G.M., Izpisúa Belmonte J.C.I. (2009). Linking the p53 tumor suppressor pathway to somatic cell reprogramming. Nature.

[16] Brennan C.W., Verhaak R.G.W., McKenna A., Campos B., Noushmehr H., Salama S.R., Zheng S., Chakravarty D., Sanborn J.Z., Berman S.H. (2013). The somatic genomic landscape of glioblastoma. Cell.

[17] Liu H., Jia D., Li A., Chau J., He D., Ruan X., Liu F., Li J., He L., Li B. (2013). p53 regulates neural stem cell proliferation and differentiation via BMP-Smad1 signaling and Id1. Stem Cells Dev..

[18] Meletis K., Wirta V., Hede S.M., Nistér M., Lundeberg J., Frisén J. (2006). p53 suppresses the self-renewal of adult neural stem cells. Development.

[19] Gil-Perotin S., Marin-Husstege M., Li J., Soriano-Navarro M., Zindy F., Roussel M.F., Garcia-Verdugo J.-M., Casaccia-Bonnefil P. (2006). Loss of p53 induces changes in the behavior of subventricular zone cells: implication for the genesis of glial tumors. J. Neurosci..

[20] Li J., Pan L., Pembroke W.G., Rexach J.E., Godoy M.I., Condro M.C., Alvarado A.G., Harteni M., Chen Y.-W., Stiles L. (2021). Conservation and divergence of vulnerability and responses to stressors between human and mouse astrocytes. Nat. Commun..

[21] Robel S., Berninger B., Götz M. (2011). The stem cell potential of glia: lessons from reactive gliosis. Nat. Rev. Neurosci..

[22] Sirko S., Irmler M., Gascón S., Bek S., Schneider S., Dimou L., Obermann J., De Souza Paiva D., Poirier F., Beckers J. (2015). Astrocyte reactivity after brain injury-: the role of galectins 1 and 3. Glia.

[23] Hirrlinger P.G., Scheller A., Braun C., Hirrlinger J., Kirchhoff F. (2006). Temporal control of gene recombination in astrocytes by transgenic expression of the tamoxifen-inducible DNA recombinase variant CreERT2. Glia.

[24] Marino S., Vooijs M., van Der Gulden H., Jonkers J., Berns A. (2000). Induction of medulloblastomas in P53-null mutant mice by somatic inactivation of Rb in the external granular layer cells of the cerebellum. Genes Dev..

[25] Madisen L., Zwingman T.A., Sunkin S.M., Oh S.W., Zariwala H.A., Gu H., Ng L.L., Palmiter R.D., Hawrylycz M.J., Jones A.R. (2010). A robust and high-throughput Cre reporting and characterization system for the whole mouse brain. Nat. Neurosci..

[26] Benedykcinska A., Ferreira A., Lau J., Broni J., Richard-Loendt A., Henriquez N.V., Brandner S. (2015). Generation of brain tumors by Cre-mediated recombination of neural progenitors in situ with the tamoxifen metabolite endoxifen. Dis. Models Mech..

[27] Faiz M., Sachewsky N., Gascón S., Bang K.W., Morshead C.M., Nagy A. (2015). Adult neural stem cells from the subventricular zone give rise to reactive astrocytes in the cortex after stroke. Cell Stem Cell.

[28] Burda J.E., Sofroniew M.V. (2014). Reactive gliosis and the multicellular response to CNS damage and disease. Neuron.

[29] Mori T., Tanaka K., Buffo A., Wurst W., Kühn R., Götz M. (2006). Inducible gene deletion in astroglia and radial glia-A valuable tool for functional and lineage analysis. Glia.

[30] Norden D.M., Godbout J.P. (2013). Review: Microglia of the aged brain: primed to be activated and resistant to regulation. Neuropathol. Appl. Neurobiol..

[31] Wyss-Coray T. (2016). Aging, neurodegeneration and brain rejuvenation. Nature.

[32] Buttini M., Boddeke H. (1995). Peripheral lipopolysaccharide stimulation induces interleukin-1β messenger RNA in rat brain microglial cells. Neuroscience.

[33] Hoogland I.C.M., Houbolt C., Van Westerloo D.J., Van Gool W.A., Van De Beek D. (2015). Systemic inflammation and microglial activation: systematic review of animal experiments. J. Neuroinflammation.

[34] Batista C.R.A., Gomes G.F., Candelario-Jalil E., Fiebich B.L., De Oliveira A.C.P. (2019). Lipopolysaccharide-induced neuroinflammation as a bridge to understand neurodegeneration. Int. J. Mol. Sci..

[35] Dulken B.W., Leeman D.S., Boutet S.C., Hebestreit K., Brunet A. (2017). Single-cell transcriptomic analysis defines heterogeneity and transcriptional dynamics in the adult neural stem cell lineage. Cell Rep..

[36] Guttenplan K.A., Weigel M.K., Adler D.I., Couthouis J., Liddelow S.A., Gitler A.D., Barres B.A. (2020). Knockout of reactive astrocyte activating factors slows disease progression in an ALS mouse model. Nat. Commun..

[37] Gyoneva S., Hosur R., Gosselin D., Zhang B., Ouyang Z., Cotleur A.C., Peterson M., Allaire N., Challa R., Cullen P. (2019). Cx3cr1-deficient microglia exhibit a premature aging transcriptome. Life Sci. Alliance.

[38] Foo L.C. (2013). Purification of rat and mouse astrocytes by immunopanning. Cold Spring Harbor Protoc..

[39] Scholze A.R., Foo L.C., Mulinyawe S., Barres B.A. (2014). BMP signaling in astrocytes downregulates EGFR to modulate survival and maturation. PLoS One.

[40] Bayraktar O.A., Bartels T., Holmqvist S., Kleshchevnikov V., Martirosyan A., Polioudakis D., Ben Haim L., Young A.M.H., Batiuk M.Y., Prakash K. (2020). Astrocyte layers in the mammalian cerebral cortex revealed by a single-cell in situ transcriptomic map. Nat. Neurosci..

[41] Batiuk M.Y., Martirosyan A., Wahis J., De Vin F., Marneffe C., Kusserow C., Koeppen J., Viana J.F., Oliveira J.F., Voet T. (2020). Identification of region-specific astrocyte subtypes at single cell resolution. Nat. Commun..

[42] Weinreb C., Wolock S., Klein A.M. (2018). Spring: a kinetic interface for visualizing high dimensional single-cell expression data. Bioinformatics.

[43] Llorens-Bobadilla E., Zhao S., Baser A., Saiz-Castro G., Zwadlo K., Martin-Villalba A. (2015). Single-cell transcriptomics reveals a population of dormant neural stem cells that become activated upon brain injury. Cell Stem Cell.

[44] Shin J., Berg A., Daniel Z.Y., Shin Y., Joseph S.J., Bonaguidi A., Michael E.G., Nauen W., David C.M., Kimberly M.G.-L., Song H. (2015). Single-Cell RNA-Seq with Waterfall Reveals Molecular Cascades underlying Adult Neurogenesis. Cell Stem Cell.

[45] Laywell E.D., Rakic P., Kukekov V.G., Holland E.C., Steindler D.A. (2000). Identification of a multipotent astrocytic stem cell in the immature and adult mouse brain. Proc. Natl. Acad. Sci. USA.

[46] Korsunsky I., Millard N., Fan J., Slowikowski K., Zhang F., Wei K., Baglaenko Y., Brenner M., Loh P.R., Raychaudhuri S. (2019). Fast, sensitive and accurate integration of single-cell data with Harmony. Nat. Methods.

[47] Baser A., Skabkin M., Kleber S., Dang Y., Gülcüler Balta G.S., Kalamakis G., Göpferich M., Ibañez D.C., Schefzik R., Lopez A.S. (2019). Onset of differentiation is post-transcriptionally controlled in adult neural stem cells. Nature.

[48] Romano R., Bucci C. (2020). Role of EGFR in the nervous system. Cells.

[49] Kawase T., Ohki R., Shibata T., Tsutsumi S., Kamimura N., Inazawa J., Ohta T., Ichikawa H., Aburatani H., Tashiro F., Taya Y. (2009). PH domain-only protein PHLDA3 is a p53-regulated repressor of Akt. Cell.

[50] Budanov A.V., Karin M. (2008). p53 target genes Sestrin1 and Sestrin2 connect genotoxic stress and mTOR signaling. Cell.

[51] Thoreen C.C., Chantranupong L., Keys H.R., Wang T., Gray N.S., Sabatini D.M. (2012). A unifying model for mTORC1-mediated regulation of mRNA translation. Nature.

[52] Magnusson J.P., Frisén J. (2016). Stars from the darkest night: unlocking the neurogenic potential of astrocytes in different brain regions. Development.

[53] Amit M., Takahashi H., Dragomir M.P., Lindemann A., Gleber-Netto F.O., Pickering C.R., Anfossi S., Osman A.A., Cai Y., Wang R. (2020). Loss of p53 drives neuron reprogramming in head and neck cancer. Nature.

[54] Boutelle A.M., Attardi L.D. (2021). p53 and tumor suppression: it takes a network. Trends Cell Biol..

[55] Kim N.H., Kim H.S., Li X.-Y., Lee I., Choi H.-S., Kang S.E., Cha S.Y., Ryu J.K., Yoon D., Fearon E.R. (2011). A p53/miRNA-34 axis regulates Snail1-dependent cancer cell epithelial–mesenchymal transition. J. Cell Biol..

[56] Choi Y.J., Lin C.-P., Ho J.J., He X., Okada N., Bu P., Zhong Y., Kim S.Y., Bennett M.J., Chen C. (2011). miR-34 miRNAs provide a barrier for somatic cell reprogramming. Nat. Cell Biol..

[57] Marjanovic N.D., Hofree M., Chan J.E., Canner D., Wu K., Trakala M., Hartmann G.G., Smith O.C., Kim J.Y., Evans K.V. (2020). Emergence of a high-plasticity cell state during lung cancer evolution. Cancer Cell.

[58] Martincorena I., Campbell P.J. (2015). Somatic mutation in cancer and normal cells. Science.

[59] Martincorena I., Fowler J.C., Wabik A., Lawson A.R.J., Abascal F., Hall M.W.J., Cagan A., Murai K., Mahbubani K., Stratton M.R. (2018). Somatic mutant clones colonize the human esophagus with age. Science.

[60] Yizhak K., Aguet F., Kim J., Hess J.M., Kübler K., Grimsby J., Frazer R., Zhang H., Haradhvala N.J., Rosebrock D. (2019). RNA sequence analysis reveals macroscopic somatic clonal expansion across normal tissues. Science.

[61] Moore L., Leongamornlert D., Coorens T.H.H., Sanders M.A., Ellis P., Dentro S.C., Dawson K.J., Butler T., Rahbari R., Mitchell T.J. (2020). The mutational landscape of normal human endometrial epithelium. Nature.

[62] Lee-Six H., Olafsson S., Ellis P., Osborne R.J., Sanders M.A., Moore L., Georgakopoulos N., Torrente F., Noorani A., Goddard M. (2019). The landscape of somatic mutation in normal colorectal epithelial cells. Nature.

[63] Codega P., Silva-Vargas V., Paul A., Maldonado-Soto A.R., Deleo A.M., Pastrana E., Doetsch F. (2014). Prospective identification and purification of quiescent adult neural stem cells from their in vivo niche. Neuron.

[64] Verhaak R.G.W., Hoadley K.A., Purdom E., Wang V., Qi Y., Wilkerson M.D., Miller C.R., Ding L., Golub T., Mesirov J.P. (2010). Integrated genomic analysis identifies clinically relevant subtypes of glioblastoma characterized by abnormalities in PDGFRA, IDH1, EGFR, and NF1. Cancer Cell.

[65] Alcantara Llaguno S., Sun D., Pedraza A.M., Vera E., Wang Z., Burns D.K., Parada L.F. (2019). Cell-of-origin susceptibility to glioblastoma formation declines with neural lineage restriction. Nat. Neurosci..

[66] Azzarelli R., Simons B.D., Philpott A. (2018). The developmental origin of brain tumors: a cellular and molecular framework. Development.

[67] Leonard J.R., D'Sa C., Klocke B.J., Roth K.A. (2001). Neural precursor cell apoptosis and glial tumorigenesis following transplacental ethyl-nitrosourea exposure. Oncogene.

[68] Akgül S., Li Y., Zheng S., Kool M., Treisman D.M., Li C., Wang Y., Gröbner S., Ikenoue T., Shen Y. (2018). Opposing tumor-promoting and -Suppressive functions of Rictor/mTORC2 signaling in adult glioma and pediatric SHH medulloblastoma. Cell Rep..

[69] Li Y., Li B., Li W., Wang Y., Akgül S., Treisman D.M., Heist K.A., Pierce B.R., Hoff B., Ho C.Y. (2020). Murine models of IDH-wild-type glioblastoma exhibit spatial segregation of tumor initiation and manifestation during evolution. Nat. Commun..

[70] Wang Y., Yang J., Zheng H., Tomasek G.J., Zhang P., McKeever P.E., Lee E.Y., Zhu Y. (2009). Expression of mutant p53 proteins implicates a lineage relationship between neural stem cells and malignant astrocytic glioma in a murine model. Cancer Cell.

[71] Boscolo Sesillo F., Fox D., Sacco A. (2019). Muscle stem cells give rise to rhabdomyosarcomas in a severe mouse model of Duchenne muscular dystrophy. Cell Rep..

[72] Tyagi V., Theobald J., Barger J., Bustoros M., Bayin N.S., Modrek A.S., Kader M., Anderer E.G., Donahue B., Fatterpekar G., Placantonakis D.G. (2016). Traumatic brain injury and subsequent glioblastoma development: review of the literature and case reports. Surg. Neurol. Int..

[73] Anselmi E., Vallisa D., Bertè R., Vanzo C., Cavanna L. (2006). Post-traumatic glioma: report of two cases. Tumori.

[74] Zhou B., Liu W. (2010). Post-traumatic glioma: report of one case and review of the literature. Int. J. Med. Sci..

[75] Coskun S., Coskun A., Gursan N., Aydin M.D. (2011). Post-traumatic glioblastoma multiforme: a case report. Eurasian J. Med..

[76] Juškys R., Chomanskis Ž. (2020). Glioblastoma following traumatic brain injury: case report and literature review. Cureus.

[77] Stensjøen A.L., Solheim O., Kvistad K.A., Håberg A.K., Salvesen Ø., Berntsen E.M. (2015). Growth dynamics of untreated glioblastomas in vivo. Neuro. Oncol.

[78] Burger P.C., Dubois P.J., Schold S.C., Smith K.R., Odom G.L., Crafts D.C., Giangaspero F. (1983). Computerized tomographic and pathologic studies of the untreated, quiescent, and recurrent glioblastoma multiforme. J. Neurosurg..

[79] De Bonis P., Anile C., Pompucci A., Fiorentino A., Balducci M., Chiesa S., Lauriola L., Maira G., Mangiola A. (2013). The influence of surgery on recurrence pattern of glioblastoma. Clin. Neurol. Neurosurg..

[80] Okolie O., Bago J.R., Schmid R.S., Irvin D.M., Bash R.E., Miller C.R., Hingtgen S.D. (2016). Reactive astrocytes potentiate tumor aggressiveness in a murine glioma resection and recurrence model. Neuro. Oncol.

[81] Turnquist C., Harris B.T., Harris C.C. (2020). Radiation-induced brain injury: current concepts and therapeutic strategies targeting neuroinflammation. Neurooncol. Adv..

[82] Niraula A., Sheridan J.F., Godbout J.P. (2017). Microglia priming with aging and stress. Neuropsychopharmacology.

[83] Lourbopoulos A., Ertürk A., Hellal F. (2015). Microglia in action: how aging and injury can change the brain's guardians. Front. Cell. Neurosci..

[84] Gan S., Shi W., Wang S., Sun Y., Yin B., Bai G., Jia X., Sun C., Niu X., Wang Z. (2021). Accelerated brain aging in mild traumatic brain injury: longitudinal pattern recognition with white matter integrity. J. Neurotrauma.

[85] Godar S., Ince T.A., Bell G.W., Feldser D., Donaher J.L., Bergh J., Liu A., Miu K., Watnick R.S., Reinhardt F. (2008). Growth-inhibitory and tumor- suppressive functions of p53 depend on its repression of CD44 expression. Cell.

[86] Moffat J., Grueneberg D.A., Yang X., Kim S.Y., Kloepfer A.M., Hinkle G., Piqani B., Eisenhaure T.M., Luo B., Grenier J.K. (2006). A lentiviral RNAi library for human and mouse genes applied to an arrayed viral high-content screen. Cell.

[87] Tang W., Bertaux F., Thomas P., Stefanelli C., Saint M., Marguerat S., Shahrezaei V. (2020). bayNorm: bayesian gene expression recovery, imputation and normalization for single-cell RNA-sequencing data. Bioinformatics.

[88] Stuart T., Butler A., Hoffman P., Hafemeister C., Papalexi E., Mauck W.M., Hao Y., Stoeckius M., Smibert P., Satija R. (2019). Comprehensive integration of single-cell data. Cell.

[89] Ewels P.A., Peltzer A., Fillinger S., Patel H., Alneberg J., Wilm A., Garcia M.U., Di Tommaso P., Nahnsen S. (2020). The nf-core framework for community-curated bioinformatics pipelines. Nat. Biotechnol..

[90] McQuin C., Goodman A., Chernyshev V., Kamentsky L., Cimini B.A., Karhohs K.W., Doan M., Ding L., Rafelski S.M., Thirstrup D. (2018). CellProfiler 3.0: next-generation image processing for biology. PLoS Biol..

[91] Schildge S., Bohrer C., Beck K., Schachtrup C. (2013). Isolation and culture of mouse cortical astrocytes. J. Vis. Exp..

[92] Sun X., Hu X., Wang D., Yuan Y., Qin S., Tan Z., Gu Y., Huang X., He C., Su Z. (2017). Establishment and characterization of primary astrocyte culture from adult mouse brain. Brain Res. Bull..

[93] Pollard S.M., Yoshikawa K., Clarke I.D., Danovi D., Stricker S., Russell R., Bayani J., Head R., Lee M., Bernstein M. (2009). Glioma stem cell lines expanded in adherent culture have tumor-specific phenotypes and are suitable for chemical and genetic screens. Cell Stem Cell.

[94] Belenguer G., Domingo-Muelas A., Ferrón S.R., Morante-Redolat J.M., Fariñas I. (2016). Isolation, culture and analysis of adult subependymal neural stem cells. Differentiation.

[95] Schindelin J., Arganda-Carreras I., Frise E., Kaynig V., Longair M., Pietzsch T., Preibisch S., Rueden C., Saalfeld S., Schmid B. (2012). Fiji: an open-source platform for biological-image analysis. Nat. Methods.

[96] Hagemann-Jensen M., Ziegenhain C., Chen P., Ramsköld D., Hendriks G.J., Larsson A.J.M., Faridani O.R., Sandberg R. (2020). Single-cell RNA counting at allele and isoform resolution using Smart-seq3. Nat. Biotechnol..

[97] Grün D. (2020). Revealing dynamics of gene expression variability in cell state space. Nat. Methods.

[98] Finak G., McDavid A., Yajima M., Deng J., Gersuk V., Shalek A.K., Slichter C.K., Miller H.W., McElrath M.J., Prlic M. (2015). MAST: a flexible statistical framework for assessing transcriptional changes and characterizing heterogeneity in single-cell RNA sequencing data. Genome Biol..

[99] Subramanian A., Tamayo P., Mootha V.K., Mukherjee S., Ebert B.L., Gillette M.A., Paulovich A., Pomeroy S.L., Golub T.R., Lander E.S. (2005). Gene set enrichment analysis: a knowledge-based approach for interpreting genome-wide expression profiles. Proc. Natl. Acad. Sci. USA.

[100] Mootha V.K., Lindgren C.M., Eriksson K.F., Subramanian A., Sihag S., Lehar J., Puigserver P., Carlsson E., Ridderstråle M., Laurila E. (2003). PGC-1alpha-responsive genes involved in oxidative phosphorylation are coordinately downregulated in human diabetes. Nat. Genet..

[101] Bergen V., Lange M., Peidli S., Wolf F.A., Theis F.J. (2020). Generalizing RNA velocity to transient cell states through dynamical modeling. Nat. Biotechnol..

[102] La Manno G., Soldatov R., Zeisel A., Braun E., Hochgerner H., Petukhov V., Lidschreiber K., Kastriti M.E., Lönnerberg P., Furlan A. (2018). RNA velocity of single cells. Nature.

[103] Cao J., Spielmann M., Qiu X., Huang X., Ibrahim D.M., Hill A.J., Zhang F., Mundlos S., Christiansen L., Steemers F.J. (2019). The single-cell transcriptional landscape of mammalian organogenesis. Nature.

[104] Angerer P., Haghverdi L., Büttner M., Theis F.J., Marr C., Buettner F. (2016). destiny: diffusion maps for large-scale single-cell data in R. Bioinformatics.

[105] Hafemeister C., Satija R. (2019). Normalization and variance stabilization of single-cell RNA-seq data using regularized negative binomial regression. Genome Biol..

[106] Zhu L.J., Gazin C., Lawson N.D., Pagès H., Lin S.M., Lapointe D.S., Green M.R. (2010). ChIPpeakAnno: a Bioconductor package to annotate ChIP-seq and ChIP-chip data. BMC Bioinformatics.

[107] Andreassi C., Luisier R., Crerar H., Darsinou M., Blokzijl-Franke S., Lenn T., Luscombe N.M., Cuda G., Gaspari M., Saiardi A., Riccio A. (2021). Cytoplasmic cleavage of IMPA1 3′ UTR is necessary for maintaining axon integrity. Cell Rep..

[108] Johannes G., Sarnow P. (1998). Cap-independent polysomal association of natural mRNAs encoding c-myc, BiP, and eIF4G conferred by internal ribosome entry sites. RNA.

[109] Picelli S., Faridani O.R., Björklund A.K., Winberg G., Sagasser S., Sandberg R. (2014). Full-length RNA-seq from single cells using Smart-seq2. Nat. Protoc..

[110] Singh B., Carpenter G., Coffey R.J. (2016). EGF receptor ligands: recent advances. F1000Res.

