# Supplementary material for: Injury primes mutation-bearing astrocytes for dedifferentiation in later life
Source: Curr Biol. 2023 Mar 27;33(6):1082–1098.e8. doi: 10.1016/j.cub.2023.02.013 (PMC10615847; doi:10.1016/j.cub.2023.02.013)
Supplement: Document S1. Figures S1–S6 and Tables S1 and S2 [file mmc1.pdf]

Current Biology, Volume *33*

Supplemental Information

Injury primes mutation-bearing astrocytes

for dedifferentiation in later life

Holly Simpson Ragdale, Melanie Clements, Wenhao Tang, Elitza Deltcheva, Catia Andreassi, Alvina G. Lai, Wai Hoong Chang, Maria Pandrea, Ivan Andrew, Laurence Game, Imran Uddin, Michael Ellis, Tariq Enver, Antonella Riccio, Samuel Marguerat, and Simona Parrinello

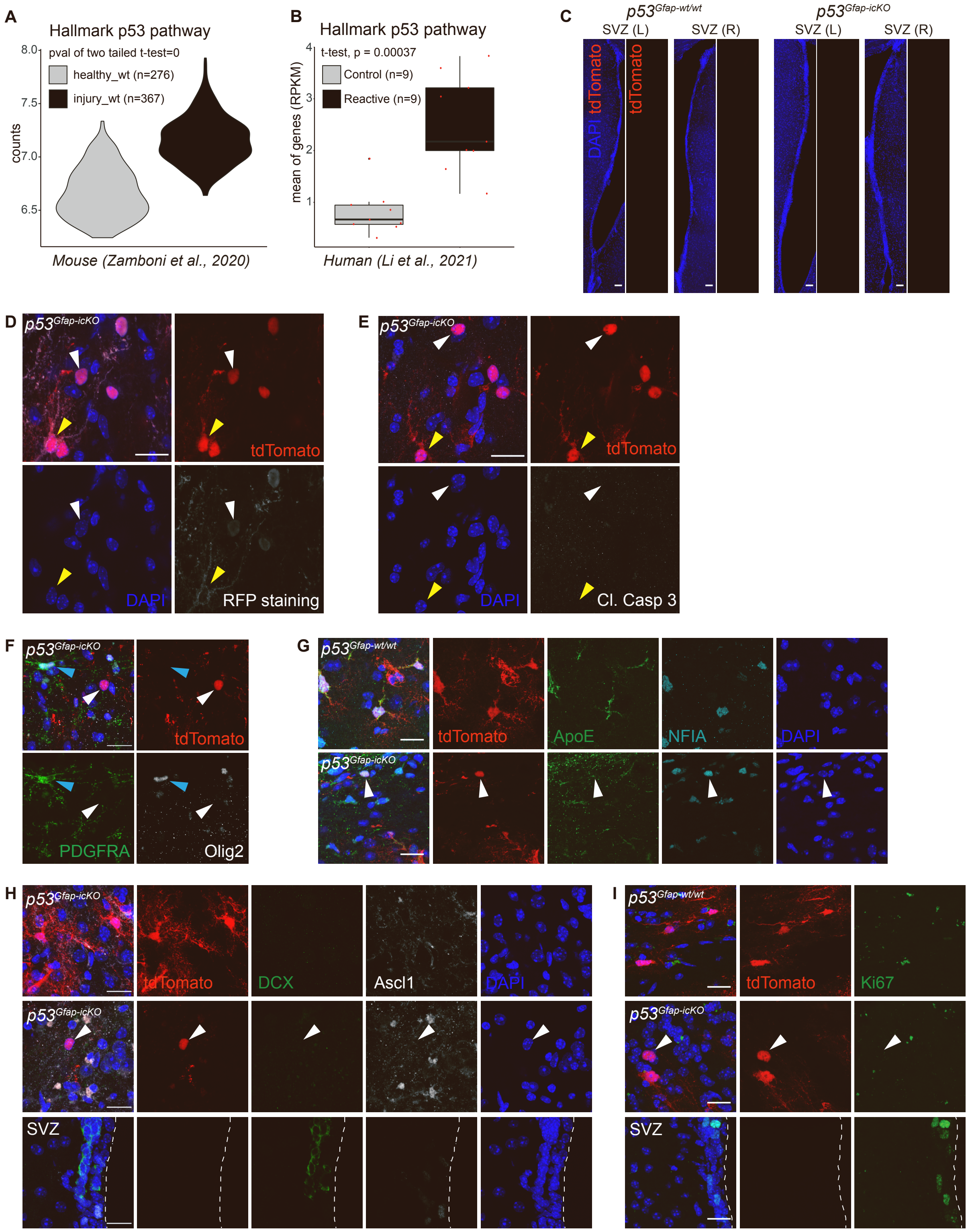

Figure S1. Characterisation of astrocyte-derived tdTomato⁺ cell state, related to Figure 1. A, Analysis of the scRNA-seq dataset from Zamboni et al.[S1] showing a significant increase in p53 pathway gene signatures after injury in mouse cortical astrocytes *in vivo*. **B,** Analysis of RNA-seq dataset from Li et al[S2] shows a significant increase in p53 pathway signatures upon activation in an *in vitro* human astrocyte model of neuroinflammation (TNFα treatment). **C,** Representative images of the subventricular zone (SVZ) from brains presented in Figure 1B, demonstrating that

recombination is localised to endoxifen injection site and not observed in the SVZ. Scale bar=50μm. **D-I,** Representative immunofluorescence images of $p53^{Gfap\text{-}wt/wt}$ or $p53^{Gfap\text{-}icKO}$ brains 6 weeks after endoxifen intracortical injection stained for the indicated marker proteins. Arrowheads indicate astrocytes (yellow) and astrocyte-derived tdTomato$^+$ (AD-tdTomato$^+$, white) cells. Scale bar=20μm. **D,** Red fluorescent protein (RFP) staining indicates process loss in AD-tdTomato$^+$ cells is not due to tdTomato fluorophore bleaching. **E,** tdTomato$^+$ astrocytes and AD-tdTomato$^+$ cells are not positive for cleaved caspase-3. **F,** AD-tdTomato$^+$ cells are Olig2$^-$/PDGFRα^- and therefore not OPCs. Blue arrowhead indicates a representative Olig2$^+$/PDGFRα^+ OPC. **G,** Astrocytes and AD-tdTomato$^+$ cells are NFIA$^+$ but lose ApoE expression. **H,** tdTomato$^+$ astrocytes and AD-tdTomato$^+$ cells are Ascl1 and doublecortin (DCX) negative. SVZ was included as positive control for staining. **I,** tdTomato$^+$ astrocytes and AD-tdTomato$^+$ cells are Ki67 negative. SVZ was included as positive control for staining.

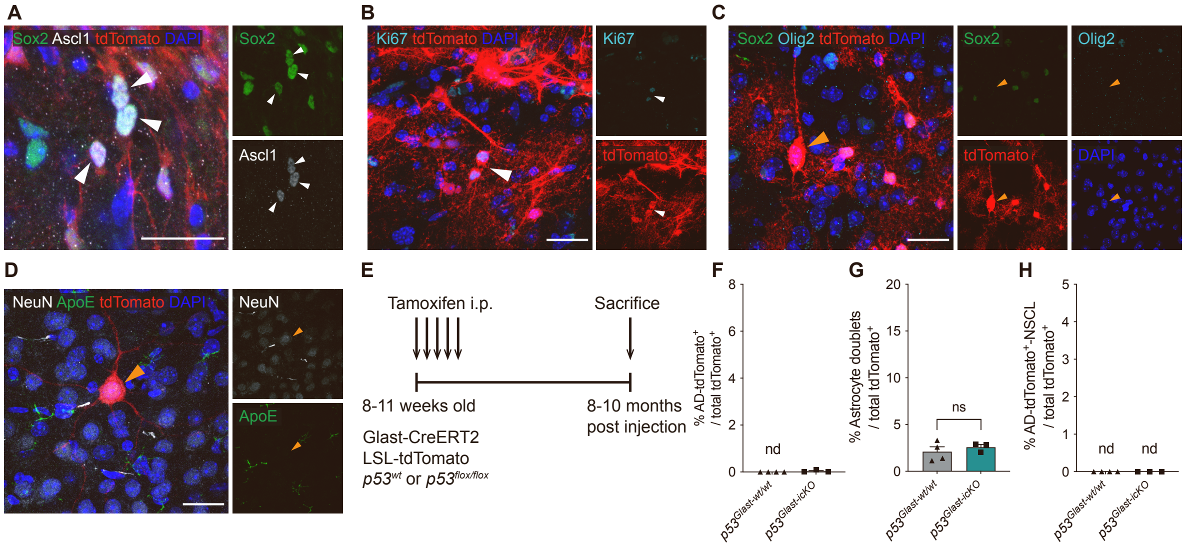

Figure S2. The ageing microenvironment drives dedifferentiation of p53-deficient cortical astrocytes, related to Figure 2. A, Representative image of Ascl1^{+} AD-tdTomato^{+} neural stem-like (AD-tdTomato^{+}-NSCL) cells (white arrowhead) in *p53$^{Gfap\text{-}icKO}$* aged cortex 10 months post injection. Scale bar=25μm. **B,** Representative image of Ki67^{+} cell pair (white arrowhead) in *p53$^{Gfap\text{-}icKO}$* aged cortex. Scale bar=25μm. **C,** Representative image of Sox2^{-}/Olig2^{-} tdTomato^{+} astrocyte-derived cell of neuronal morphology (orange arrowhead) in *p53$^{Gfap\text{-}icKO}$* aged cortex. Scale bar=25μm. **D,** Representative image of a tdTomato^{+} astrocyte-derived cell of neuronal morphology that acquired NeuN expression (orange arrowhead) in *p53$^{Gfap\text{-}icKO}$* aged cortex. Scale bar=25μm. **E,** Schematic of experimental outline for (F-H). 8-11 week old *p53$^{Glast\text{-}wt/wt}$* or *p53$^{Glast\text{-}icKO}$* mice were injected with tamoxifen i.p. for 5 days. 8-10 months after injections, mice were sacrificed and tdTomato^{+} fate-mapped astrocytes assessed. **F-H,** Quantification of indicated tdTomato^{+} cell populations in the absence of injury. Mean+SEM, aged *p53$^{Glast\text{-}wt/wt}$* n=4, aged *p53$^{Glast\text{-}icKO}$* n=3, ns not significant. **F** Quantification of AD-tdTomato^{+} cells, shown as percentage of total tdTomato^{+} cells. Unpaired two-tailed t-test. **G** Quantification of tdTomato^{+} astrocyte doublets, shown as percentage of total tdTomato^{+} cells. Unpaired two-tailed t-test. Note, scale of graph has been matched for comparison to Figure 2D. **H** Quantification of Sox2^{+}/Olig2^{+} AD-tdTomato^{+}-NSCL cells, shown as percentage of total tdTomato^{+} cells.

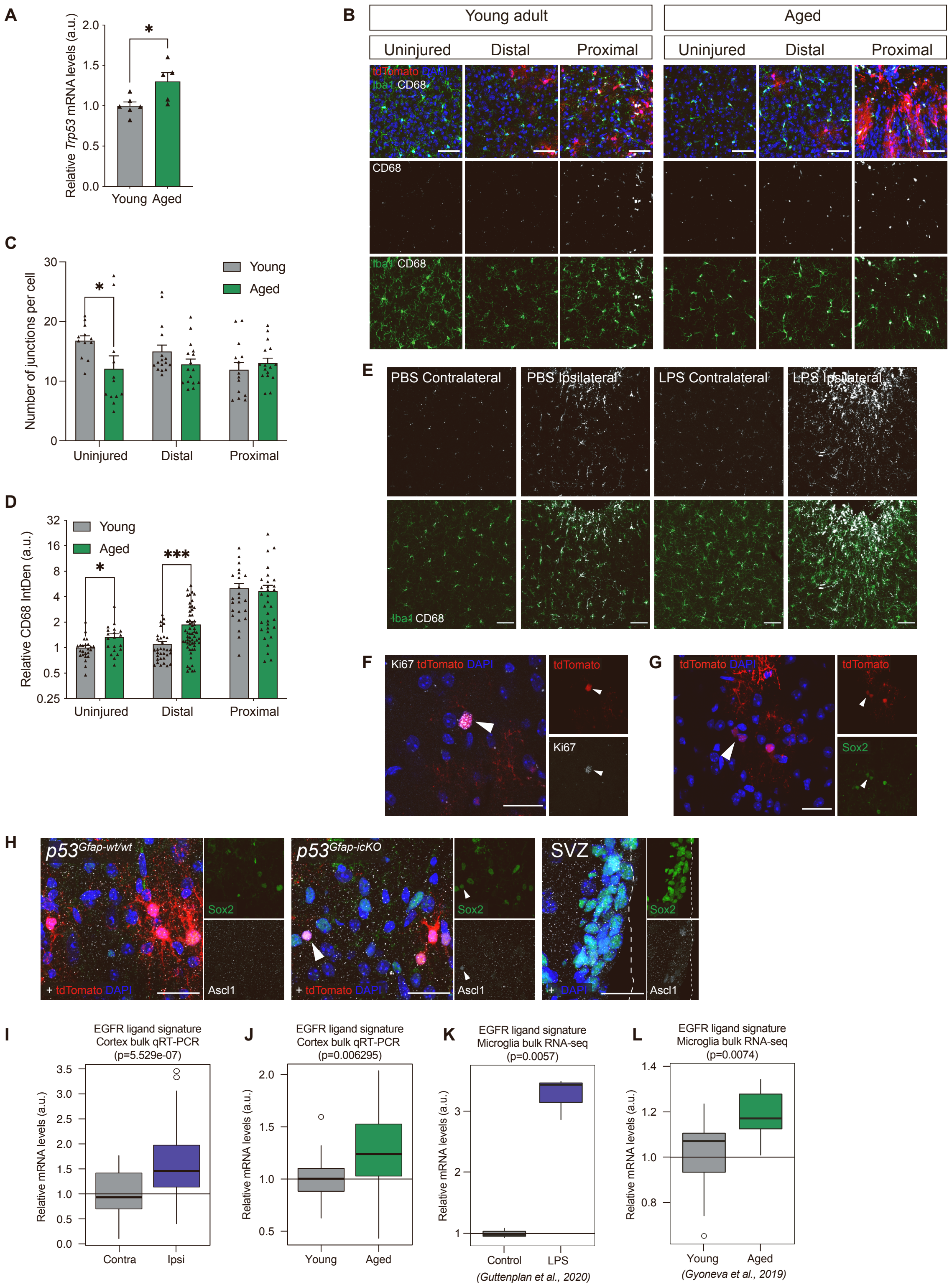

Figure S3. Increased neuroinflammation underlies age-dependent dedifferentiation of p53-deficient astrocytes following early life injury, related to Figure 3. A, qRT-PCR analysis of *Trp53* mRNA levels in astrocytes acutely purified from young (2-3 month old) or aged (1 year old) mouse cortex. Mean±SEM, n=6 young, n=5 aged, *p<0.05, unpaired two-tailed t test. **B,** Representative images of microglia in intact cortices, or in the region distal or proximal to the injection site of

young adults or aged mice. Tissue was immunostained for Iba1 and CD68 to analyse microglia morphology and activation, respectively. Scale bar=50μm. **C,** Skeleton analysis Iba1 staining of microglia in experiments from B. n=3 wildtype animals per condition. Each point represents one field of view. Mean±SEM, a.u. arbitrary units, *p<0.05, Multiple unpaired t tests with Holm-Šídák's multiple comparisons test. **D,** Quantification of relative CD68 intensity IntDen (mean intensity multiplied by particle area) in experiments from B. n=3 wildtype animals per condition. Each point represents one field of view. Mean±SEM, a.u. arbitrary units, ns not significant, *p<0.05, ****p<0.0001, Multiple unpaired t tests with Holm-Šídák's multiple comparisons test. **E,** Representative images of microglial activation in LPS or PBS (vehicle control) injected mice 3 days after i.p. injection as described in Figure 3A, confirming efficacy of LPS treatment. Scale bar=50μm. **F,** Representative image of a $Ki67^{+}$ AD-$tdTomato^{+}$ cell (white arrowhead) in $p53^{Gfap\text{-}icKO}$ treated with LPS. Scale bar=25μm. **G,** Representative image of an AD-$tdTomato^{+}$-NSCL cell doublet (white arrowhead) in $p53^{Gfap\text{-}icKO}$ treated with LPS. Scale bar=25μm. **H,** Representative image of an $Ascl1^{+}$ AD-$tdTomato^{+}$-NSCL cell (white arrowhead) in $p53^{Gfap\text{-}icKO}$ treated with LPS. SVZ was included as positive control for Ascl1 staining. Scale bar=25μm. **I,** qRT-PCR analysis of EGFR ligand signature expression in tissue dissected from injury site (ipsilateral) or contralateral area 1 week after intracortical injection. n=5 animals. Wilcoxon one-sided paired test. **J,** qRT-PCR analysis of EGFR ligand signature expression in cortical tissue dissected from young (2-3 months old) or aged (12 months old) mice. n=3 animals. Wilcoxon one-sided unpaired test. **K,** Analysis of EGFR ligand signature expression from RNA-seq of primary microglia treated with LPS[S3]. Two-sided unpaired t-test. **L,** Analysis of EGFR ligand signature expression from RNA-seq of microglia isolated from wildtype young/aged mice[S4]. Two-sided unpaired t-test.

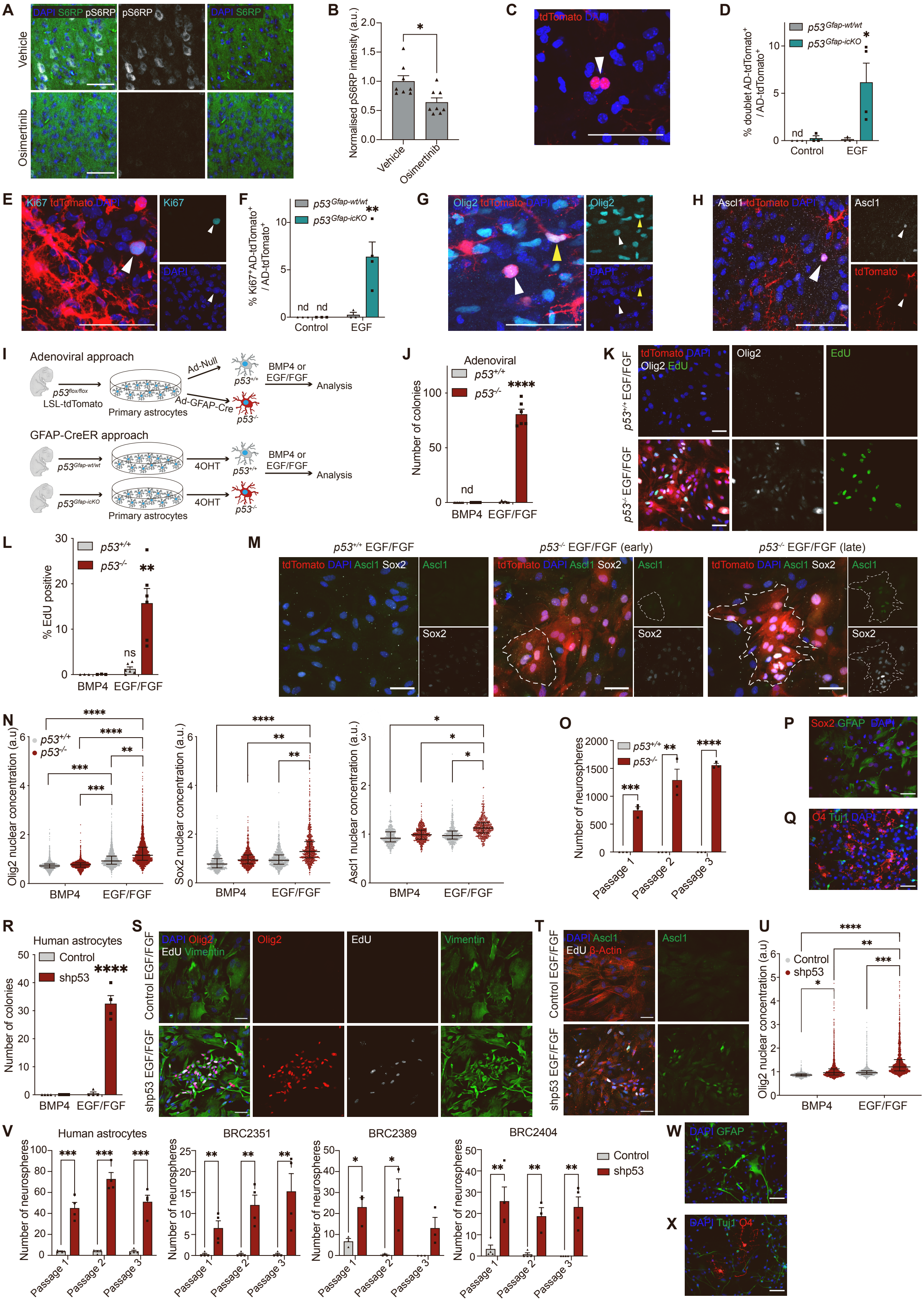
A
Vehicle
Osimertinib
DAPI S6RP pSS6RP
pS6RP
DAPI S6RP
B
Normalised pS6RP intensity (a.u.)
Vehicle
Osimertinib
C
tdTomato DAPI
D
p53Gfap-wt/wt
p53Gfap-icKO
% doublet AD-tdTomato+ / AD-tdTomato+
nd
Control
EGF
E
Ki67 tdTomato DAPI
Ki67
DAPI
F
% Ki67+ AD-tdTomato+ / AD-tdTomato+
G
Olig2 tdTomato DAPI
Olig2
DAPI
H
Ascl1 tdTomato DAPI
Ascl1
tdTomato
I
Adenoviral approach
p53flox/flox
LSL-tdTomato
Primary astrocytes
Ad-Null
Ad-GFAP-Cre
BMP4 or
EGF/FGF
Analysis
GFAP-CreER approach
p53Gfap-wt/wt
p53Gfap-icKO
4OHT
J
Adenoviral
p53+/+
p53-/-
Number of colonies
BMP4
EGF/FGF
K
p53+/+ EGF/FGF
p53-/- EGF/FGF
tdTomato DAPI
Olig2 EdU
Olig2
EdU
L
% EdU positive
ns
M
p53+/+ EGF/FGF
p53-/- EGF/FGF (early)
p53-/- EGF/FGF (late)
tdTomato DAPI Ascl1 Sox2
Ascl1
Sox2
N
Olig2 nuclear concentration (a.u)
Sox2 nuclear concentration (a.u.)
Ascl1 nuclear concentration (a.u.)
O
Number of neurospheres
Passage 1
Passage 2
Passage 3
P
Sox2 GFAP DAPI
Q
O4 Tuj1 DAPI
R
Human astrocytes
Control
shp53
S
Control EGF/FGF
shp53 EGF/FGF
DAPI Olig2
EdU Vimentin
Olig2
EdU
Vimentin
T
DAPI Ascl1
EdU β-Actin
Ascl1
U
Olig2 nuclear concentration (a.u)
V
Human astrocytes
BRC2351
BRC2389
BRC2404
W
DAPI GFAP
X
DAPI Tuj1 O4

Figure S4. Dedifferentiation of p53-deficient astrocytes is EGFR-dependent, related to Figure 4. A, Validation of osimertinib efficacy in suppressing EGFR signalling in experiment shown in Figure 4A-C by immunostaining for phospho-S6 ribosomal protein (pS6RP) and total S6RP. **B,** Quantification of pS6RP staining intensity in the cytoplasmic area of injection site shown in A, normalised to total S6RP. n=3 mice per condition. Mean±SEM, *p<0.05, unpaired two-tailed t-test. **C,** Representative image of an AD-tdTomato^{+}-NSCL cell doublet (white arrowhead) in *p53$^{Gfap\text{-}icKO}$* EGF-infused cortex. **D,** Quantification of AD-tdTomato^{+} cells occurring in doublets, shown as the percentage of total AD-tdTomato^{+} cells in EGF infusion experiment. n=3 per condition except *p53$^{Gfap\text{-}icKO}$* EGF n=4. Mean+SEM, nd not detected, *p<0.05, Two-way ANOVA with Tukey's multiple comparisons test. **E,** Representative image of a Ki67^{+} AD-tdTomato^{+}-NSCL cell (white arrowhead) in *p53$^{Gfap\text{-}icKO}$* EGF-infused cortex. **F,** Quantification of Ki67^{+} AD-tdTomato^{+} cells as percentage of total AD-tdTomato^{+} cells. n=3 per condition except *p53$^{Gfap\text{-}icKO}$* EGF n=4. Mean+SEM, nd not detected, **p<0.01, Two-way ANOVA with Tukey's multiple comparisons test. **G,** Representative image of Olig2^{+} astrocytic (yellow arrowhead) and Olig2^{+} AD-tdTomato^{+}-NSCL (white arrowhead) in *p53$^{Gfap\text{-}icKO}$* EGF cortex. **H**, Representative image of a Ascl1^{+} AD-tdTomato^{+}-NSCL cell (white arrowhead) in *p53$^{Gfap\text{-}icKO}$* EGF-infused cortex. **I,** Schematic of experimental outline. Primary cortical astrocytes were isolated from postnatal day 3 (P3) *p53$^{flox/flox}$*; LSL-tdTomato mice or GFAP-CreERT2; *p53$^{flox/flox}$* or *p53$^{wt/wt}$;* LSL-tdTomato mice. p53 recombination and tdTomato labelling was induced via adenoviral GFAP-Cre (or Null adenovirus for control; Figure S4J-Q, Figure S5F,G) or 4OHT treatment (Figure 5 and Figure S5 A-E, H-J), respectively. Cells were incubated in media supplemented with BMP4 or EGF/FGF for 7d before immunofluorescence or clonal analysis. **J,** Quantification of number of colonies visualised by crystal violet staining in indicated culture conditions following Adenovirus-induced recombination. Mean±SEM n=7, nd not detected, ****p<0.0001, Two-way ANOVA with Tukey's multiple comparisons test. **K,** Representative immunofluorescence images of EdU incorporation assays of *p53$^{+/+}$* and *p53$^{-/-}$* astrocytes cultured in EGF/FGF, indicating that colonies of tdTomato^{+} cells forming in *p53$^{-/-}$* cultures are proliferating (EdU^{+}) and Olig2^{+}. **L,** Quantification of percentage of EdU^{+} cells in each condition shown in C. Mean±SEM, n=6 independent experiments. ns not significant, **p<0.01, Two-way ANOVA with Tukey's multiple comparisons test. **M,** Representative immunofluorescence images of *p53$^{+/+}$* and *p53$^{-/-}$* astrocytes in EGF/FGF and stained for Ascl1 and Sox2, indicating that colonies of tdTomato^{+} cells forming in *p53$^{-/-}$* cultures are positive for both neurodevelopmental transcription factors. **N,** Quantification of Olig2, Sox2 and Ascl1 nuclear protein staining intensity, as indicated. Each point represents an individual cell. Line represents median with interquartile range, n=3 independent experiments. *p<0.05, **p<0.01, ***p<0.001, ****p<0.0001, Two-way ANOVA with Tukey's multiple comparisons test on average intensity per condition per replicate. **O,** Quantification of number of neurospheres formed from *p53$^{+/+}$* and *p53$^{-/-}$* astrocytes cultured in EGF/FGF for 7 days and replated in suspension. *p53$^{-/-}$* dedifferentiated astrocytes selectively form neurospheres which can be serially passaged for at least 3 passages. *p53$^{+/+}$*

astrocytes do not form neurospheres. Mean±SEM, **p<0.01, ***p<0.001, ****p<0.0001, Multiple t-tests with Two stage linear step-up procedure of Benjamin, Krieger and Yekutieli. **P-Q,** Representative immunofluorescence images of dedifferentiated *p53*$^{-/-}$ astrocytes induced to re-differentiate by mitogen removal and stained for astrocyte markers (P, GFAP^{+}, Sox2^{low}), or oligodendrocytes (Q, O4^{+}) and neuroblasts (Q, Tuj1^{+}). **R,** Quantification of number of colonies visualised by crystal violet staining in indicated culture conditions following lentiviral mediated p53 downregulation (shp53) in primary human astrocytes. Mean±SEM n=4, ****p<0.0001, Two-way ANOVA with Tukey's multiple comparisons test. **S-T,** Representative immunofluorescence images of Olig2 (**S**), Ascl1 (**T**) immunostaining and EdU incorporation of wildtype of shp53 human astrocytes in EGF/FGF, indicating that colonies of dedifferentiated cells forming in shp53 cultures are positive for both neurodevelopmental transcription factors and proliferative. Vimentin (S) and β-actin (T) were used to label cell morphology. **U,** Quantification of Olig2 nuclear protein staining intensity. Each point represents an individual cell. Line represents median with interquartile range, n=3 independent experiments. *p<0.05, **p<0.01, ***p<0.001, ****p<0.0001, Two-way ANOVA with Tukey's multiple comparisons test on average intensity per condition per replicate. **V,** Quantification of number of neurospheres formed from control or shp53 human astrocytes (primary or fetal neural stem cell-derived (BRC2351, BRC2389, BRC2404), as indicated) cultured in EGF/FGF for 7 days and replated in suspension. shp53 dedifferentiated astrocytes selectively form neurospheres which can be serially passaged for at least 3 passages. p53 wildtype control astrocytes do not form neurospheres. Mean±SEM n=4 per condition except BRC2389 n=3. *p<0.05, **p<0.01, ***p<0.001, Multiple t-tests with Two stage linear step-up procedure of Benjamin, Krieger and Yekutieli. **W-X,** Representative immunofluorescence images of shp53-dedifferentiated human astrocytes induced to re-differentiate by mitogen removal and stained for astrocyte markers (W, GFAP^{+}), or oligodendrocytes (X, O4^{+}) and neuroblasts (X, Tuj1^{+}) markers. All images, scale bar=50μm.

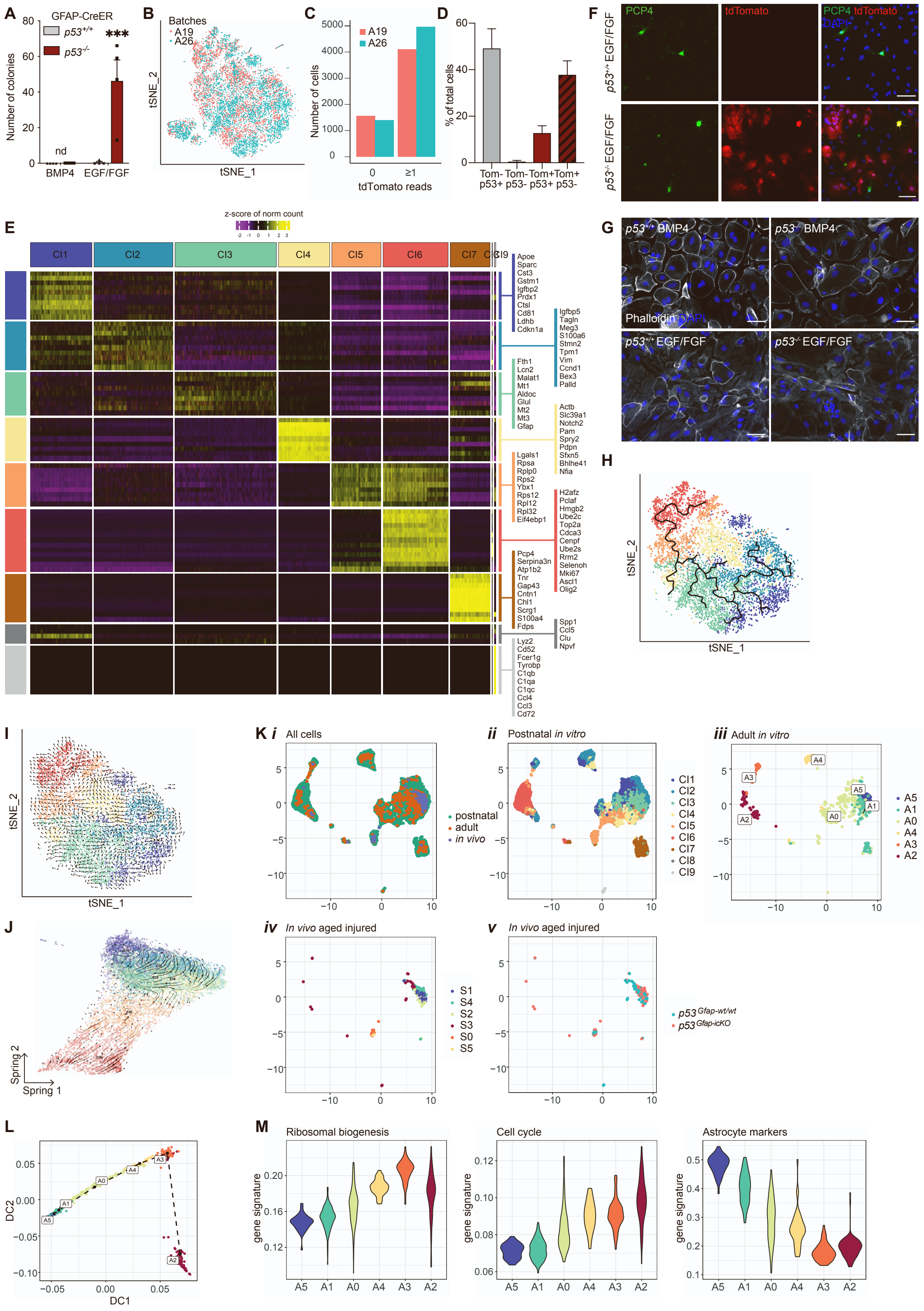

A
GFAP-CreER
p53+/+
p53-/-
***
nd
Number of colonies
BMP4
EGF/FGF
B
Batches
A19
A26
tSNE_2
tSNE_1
C
A19
A26
Number of cells
0
≥1
tdTomato reads
D
% of total cells
Tom- p53+
Tom- p53-
Tom+ p53+
Tom+ p53-
F
PCP4
tdTomato
DAPI
p53+/+ EGF/FGF
p53-/- EGF/FGF
E
z-score of norm count
Cl1
Cl2
Cl3
Cl4
Cl5
Cl6
Cl7
Cl8
Cl9
Apoe
Sparc
Cst3
Gstm1
Igfbp2
Prdx1
Ctsl
Cd81
Ldhb
Cdkn1a
Igfbp5
Tagln
Meg3
S100a6
Stmn2
Tpm1
Vim
Ccnd1
Bex3
Palld
Fth1
Lcn2
Malat1
Mt1
Aldoc
Glul
Mt2
Mt3
Gfap
Actb
Slc39a1
Notch2
Pam
Spry2
Pdpn
Sfxn5
Bhlhe41
Nfia
Lgals1
Rpsa
Rplp0
Rps2
Ybx1
Rps12
Rpl12
Rpl32
Eif4ebp1
H2afz
Pclaf
Hmgb2
Ube2c
Top2a
Cdca3
Cenpf
Ube2s
Rrm2
Selenoh
Mki67
Ascl1
Olig2
Pcp4
Serpina3n
Atp1b2
Tnr
Gap43
Cntn1
Chl1
Scrg1
S100a4
Fdps
Spp1
Ccl5
Clu
Npvf
Lyz2
Cd52
Fcer1g
Tyrobp
C1qb
C1qa
C1qc
Ccl4
Ccl3
Cd72
G
p53+/+ BMP4
p53-/- BMP4
Phalloidin DAPI
p53+/+ EGF/FGF
p53-/- EGF/FGF
H
tSNE_2
tSNE_1
I
tSNE_2
tSNE_1
J
Spring 2
Spring 1
K i All cells
postnatal
adult
in vivo
ii Postnatal in vitro
iii Adult in vitro
A5
A1
A0
A4
A3
A2
iv In vivo aged injured
S1
S4
S2
S3
S0
S5
v In vivo aged injured
p53Gfap-wt/wt
p53Gfap-icKO
L
DC2
DC1
M
Ribosomal biogenesis
Cell cycle
Astrocyte markers
gene signature

Figure S5. scRNA-seq of dedifferentiating *p53*$^{-/-}$ astrocytes *in vitro*, related to Figure 5. A, Quantification of number of colonies visualised by crystal violet staining in indicated culture conditions following 4OHT treatment. Mean±SEM, n=4 independent experiments. nd not detected, ***p<0.001, Two-way ANOVA with Tukey's multiple comparisons test. **B,** t-SNE as in Figure 5B, coloured by replicate (A19 and A26). **C,** Number of cells per replicate with tdTomato reads of 0 or ≥1, (see filtering steps in methods). **D,** Quantification of immunostaining for p53 at 5d post EGF/FGF + 4OHT treatment. Some tdTomato^{+} astrocytes retain p53 expression. Mean±SEM, n=2 independent replicates. **E,** Heatmap of z-score normalized gene expression for the 9 scRNA-seq clusters. **F**, Representative image of PCP4 immunostaining in *p53*$^{+/+}$ and *p53*$^{-/-}$ astrocyte cultures in EGF/FGF. PCP4^{+} cells are present in all conditions, indicating that these cells represent a contaminating cell population within the preparations. Scale bar=50μm. **G**, Representative image of phalloidin staining in *p53*$^{+/+}$ and *p53*$^{-/-}$ astrocyte cultures in BMP4 or EGF/FGF. Note that actin remodelling occurs in both genotypes upon mitogen exposure. Scale bar=50μm. **H,** Monocle pseudotime trajectory of tdTomato+ cells in tSNE. **I,** velocyto[S5] RNA velocity analysis overlayed onto t-SNE. **J,** scvelo pseudotime analysis of tdTomato+ cells in SPRING[S6]. **K,** Harmony analysis of integrated scRNAseq datasets (*i*) from: postnatal *in vitro* astrocytes (*ii*), adult *in vitro* astrocytes (*iii*) and *in vivo* tdTomato^{+} cells acutely FACS-purified from the cortices of aged and injured *p53*$^{Gfap\text{-}wt/wt}$ *and p53*$^{Gfap\text{-}icKO}$ mice (*iv*-*v*). Colouring is based on dataset (*i*), clusters (*ii-iv*) and genotypes (*v*). Note that dedifferentiated cells were only found in *p53*$^{Gfap\text{-}icKO}$, but not in *p53*$^{Gfap\text{-}wt/wt}$ (i*v, v*) brains. **L,** Diffusion map of adult *in vitro* astrocyte data coloured by clusters[S7]. **M,** Violin plots of pathway analysis in ordered clusters of adult *in vitro* astrocyte.

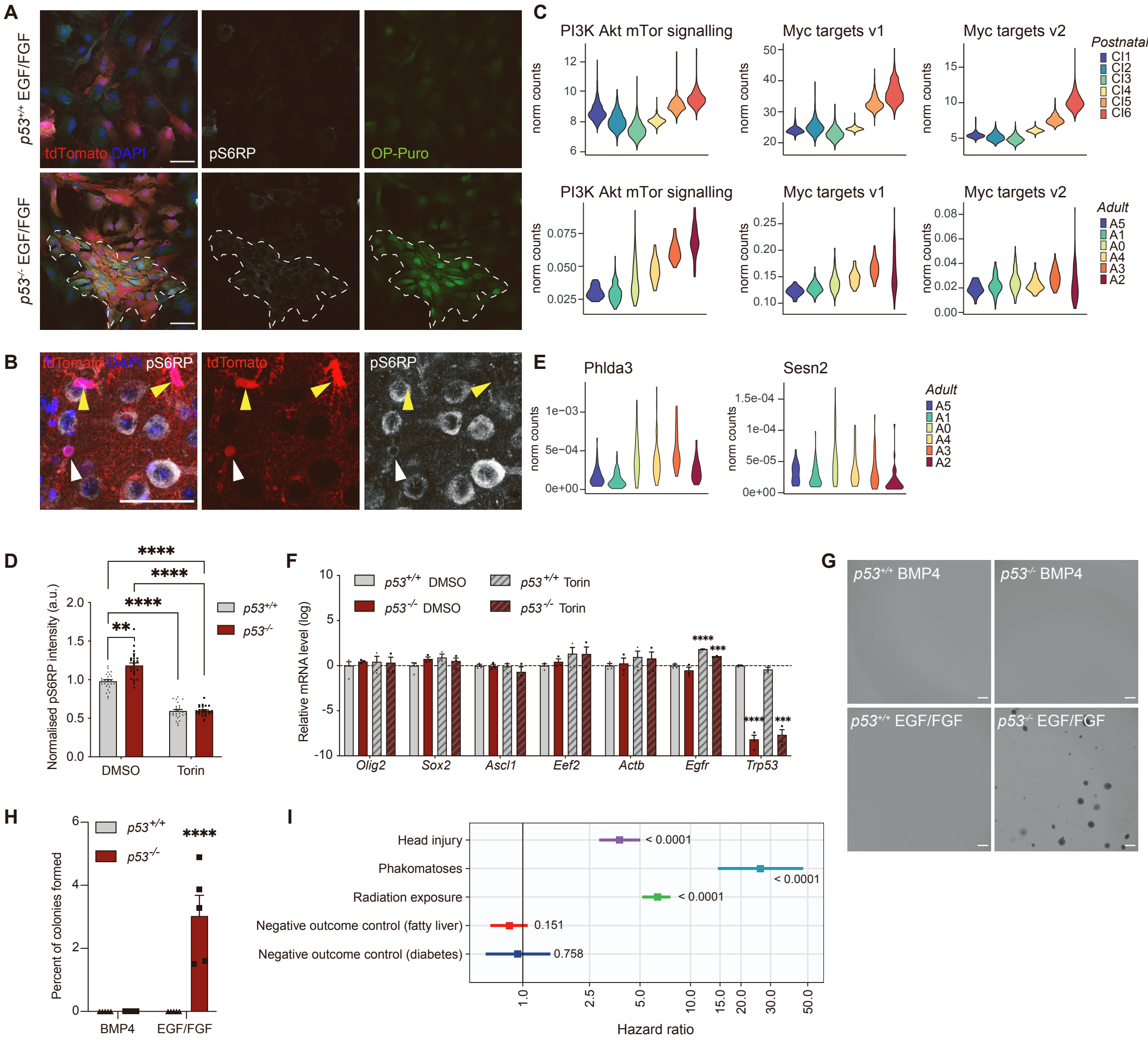

Figure S6. mTOR activity is increased upon p53 loss, related to Figure 6. A, Representative image of *p53*$^{+/+}$ and *p53*$^{-/-}$ astrocyte cultures in EGF/FGF media stained for OP-puro and pS6RP. mTOR activity correlates with increased protein synthesis, as assessed by OP-Puro incorporation. Scale bar=50μm. **B,** Representative image of pS6RP immunostaining in *p53*$^{Gfap\text{-}icKO}$ aged injured mouse. pS6RP is higher in AD-tdTomato^{+}-NSCL cells (white arrowheads) compared to astrocytic tdTomato^{+} cells (yellow arrowhead). Note that the highest expression of pS6RP is observed in tdTomato^{-} neurons. Scale bar=50μm. **C,** Violin plots of indicated genes in ordered clusters of postnatal (top) and adult (bottom) *in vitro* scRNAseq datasets. **D,** Quantification of pS6RP intensity in *p53*$^{+/+}$ or *p53*$^{-/-}$ astrocytes cultured in EGF/FGF in the presence of Torin or DMSO vehicle control, normalised to total S6RP. Torin treatment significantly reduced pS6RP intensity. Pixel intensity of cytoplasmic area of whole field of view was quantified for n=3 independent experiments. Each point indicates a field of view. Mean±SEM, **p<0.01, ****p<0.0001, Two-way ANOVA with Tukey's multiple comparisons test. **E,** Violin plots of indicated genes in the ordered clusters from adult *in vitro* scRNA-seq data. **F,** qRT-PCR of total mRNA of input of polysome profiling experiment in Figure 6G. Changes in polysome profile are not due to changes in mRNA levels of genes. ***p<0.001, ****p<0.0001, Two-way ANOVA with

Tukey's multiple comparisons test. **G-H,** Soft agar assay of in vitro astrocytes. $p53^{-/-}$ astrocytes cultured in EGF/FGF conditions showed anchorage-independence after serial passaging, while other conditions did not. Scale bar=1mm. Mean±SEM, n=5, ****$p<0.0001$, Two-way ANOVA with Tukey's multiple comparisons test. **I,** Matched cohort analysis to estimate the risk of developing glioma in people with a history of brain injury. Phakomatoses and exposure to radiation were used as positive controls as they are both known risk factors for brain cancer. Diabetes and fatty liver disease were used as negative outcome controls, showing no increased risk associated with brain injury. Hazard ratio and 95% confidence interval are shown.

Gene	Forward primer (5'-3')	Reverse primer (5'-3')	Reference (if applicable)
Actb	TCGTTGCCGGTCCACACCCG	CTCCTCAGGGGCCACACGCAG	S8
Areg	GCCATCATCCTCGCAGCTA	ATGATTCAACTTTTACCCTGCATTG	This study
Ascl1	ATGCAGCTACTGTCCAAACG	AACAGTAAGGGGTGGGTGTG	S9
Btc	AAACCCACTTCTCTCGGTGC	AAACAGGTCCACTCGCTCAC	This study
Eef2	GAGAATCCGTCGCCATCCGCCA	CGGGCTTGATGCGTTCAGCGA	S8
Egf	TCGAGAGAAGCGAGAGAAGC	TGTTCCATCTGGGTCAATCCG	This study
Egfr	TCTGCCACCTATGCCACGCCA	CCACTGCCATTGAACGTACCCAG	This study
Epgn	CATTTAACAACACCGAAGCTGACT	GCTCACATCGTTGTCCCGT	This study
Ereg	GACGCTGCTTTGTCTAGGTTCC	CACACGGGGATCGTCTTCC	This study
Gusb	AACAACACACTGACCCCTCA	ACCACAGATCGATGCAGTCC	This study
Hbegf	GTCGTCCGTCTGTCTTCTTGT	CTAGCCACGCCCAACTTCAC	This study
Olig2	CGAGCACCTCAAATCTAATTCA	GGACGATGGGCGACTAGA	S9
Ppia	GCGTCTCCTTCGAGCTGTT	AAAGTCACCACCCTGGCA	S10
Sox2	TCCAAAAACTAATCACAACAATCG	GAAGTGCAATTGGGATGAAAA	This study
Tgfa	CATTATCACCTGTGTGCTGATCC	CCTTTTCTTGGTTGGGCTGTCATA	This study
Trp53	GGACGGGACAGCTTTGAGGT	GTGGGCAGCGCTCTCTTTG	This study

Table S1. List of primer sequences used in this study, related to STAR methods.

ICD-10	Terms	Diagnostic category
S06	Intracranial injury	Head injury
S060	Concussion	Head injury
S061	Traumatic cerebral oedema	Head injury
S062	Diffuse brain injury	Head injury
S063	Focal brain injury	Head injury
S064	Epidural haemorrhage	Head injury
S065	Traumatic subdural haemorrhage	Head injury
S066	Traumatic subarachnoid haemorrhage	Head injury
S067	Intracranial injury with prolonged coma	Head injury
S068	Other intracranial injuries	Head injury
S069	Intracranial injury, unspecified	Head injury
Q850	Neurofibromatosis (nonmalignant)	Phakomatoses
Q851	Tuberous sclerosis	Phakomatoses
Q858	Other phakomatoses, not elsewhere classified	Phakomatoses
Q859	Phakomatosis, unspecified	Phakomatoses
W88	Exposure to ionizing radiation	Radiation
W91	Exposure to unspecified type of radiation	Radiation
X39	Exposure to other and unspecified forces of nature	Radiation
Y365	War operations involving nuclear weapons	Radiation
Z58	Problems related to physical environment	Radiation
Z584	Exposure to radiation	Radiation

Z923	Personal history of irradiation	Radiation
E10	Insulin-dependent diabetes mellitus	Diabetes
E11	Non-insulin-dependent diabetes mellitus	Diabetes
E12	Malnutrition-related diabetes mellitus	Diabetes
E13	Other specified diabetes mellitus	Diabetes
E14	Unspecified diabetes mellitus	Diabetes
G590	Diabetic mononeuropathy	Diabetes
G632	Diabetic polyneuropathy	Diabetes
H280	Diabetic cataract	Diabetes
H360	Diabetic retinopathy	Diabetes
M142	Diabetic arthropathy	Diabetes
N083	Glomerular disorders in diabetes mellitus	Diabetes
O240	Diabetes mellitus in pregnancy: Pre-existing diabetes mellitus, insulin-dependent	Diabetes
O241	Diabetes mellitus in pregnancy: Pre-existing diabetes mellitus, non-insulin-dependent	Diabetes
O242	Diabetes mellitus in pregnancy: Pre-existing malnutrition-related diabetes mellitus	Diabetes
O243	Diabetes mellitus in pregnancy: Pre-existing diabetes mellitus, unspecified	Diabetes
K700	Alcoholic fatty liver	Fatty liver
K758	Other specified inflammatory liver diseases	Fatty liver
K760	Fatty (change of) liver, not elsewhere classified	Fatty liver
C71	Malignant neoplasm of brain	Brain cancer
C710	Cerebrum, except lobes and ventricles	Brain cancer
C711	Frontal lobe	Brain cancer
C712	Temporal lobe	Brain cancer
C713	Parietal lobe	Brain cancer
C714	Occipital lobe	Brain cancer
C715	Cerebral ventricle	Brain cancer
C716	Cerebellum	Brain cancer
C717	Brain stem	Brain cancer
C718	Overlapping lesion of brain	Brain cancer
C719	Brain, unspecified	Brain cancer
C72	Malignant neoplasm of spinal cord, cranial nerves and other parts of central nervous system	Brain cancer
C728	Overlapping lesion of brain and other parts of central nervous system	Brain cancer
C729	Central nervous system, unspecified	Brain cancer

Table S2. Code list for conditions included in the matched cohort analysis, related to STAR methods.

Supplementary References

S1. Zamboni, M., Llorens-Bobadilla, E., Magnusson, J. P., and Frisen, J. (2020). A Widespread Neurogenic Potential of Neocortical Astrocytes Is Induced by Injury. Cell Stem Cell *27*, 605-617. 10.1016/j.stem.2020.07.006.

S2. Li, J., Pan, L., Pembroke, W. G., Rexach, J. E., Godoy, M. I., Condro, M. C., Alvarado, A. G., Harteni, M., Chen, Y.-W., Stiles, L., et al. (2021). Conservation and divergence of vulnerability and responses to stressors between human and mouse astrocytes. Nature Communications *12*, 3958. 10.1038/s41467-021-24232-3.

S3. Guttenplan, K. A., Weigel, M. K., Adler, D. I., Couthouis, J., Liddelow, S. A., Gitler, A. D., and Barres, B. A. (2020). Knockout of reactive astrocyte activating factors slows disease progression in an ALS mouse model. Nature Communications *11*. 10.1038/s41467-020-17514-9.

S4. Gyoneva, S., Hosur, R., Gosselin, D., Zhang, B., Ouyang, Z., Cotleur, A. C., Peterson, M., Allaire, N., Challa, R., Cullen, P., et al. (2019). Cx3cr1-deficient microglia exhibit a premature aging transcriptome. Life Science Alliance *2*, e201900453. 10.26508/lsa.201900453.

S5. Bergen, V., Lange, M., Peidli, S., Wolf, F. A., and Theis, F. J. (2020). Generalizing RNA velocity to transient cell states through dynamical modeling. Nature Biotechnology *38*, 1408-1414. 10.1038/s41587-020-0591-3.

S6. La Manno, G., Soldatov, R., Zeisel, A., Braun, E., Hochgerner, H., Petukhov, V., Lidschreiber, K., Kastriti, M. E., Lönnerberg, P., Furlan, A., et al. (2018). RNA velocity of single cells. Nature *560*, 494-498. 10.1038/s41586-018-0414-6.

S7. Angerer, P., Haghverdi, L., Büttner, M., Theis, F. J., Marr, C., and Buettner, F. (2016). destiny: diffusion maps for large-scale single-cell data in R. Bioinformatics *32*, 1241-1243.

S8. Thoreen, C. C., Chantranupong, L., Keys, H. R., Wang, T., Gray, N. S., and Sabatini, D. M. (2012). A unifying model for mTORC1-mediated regulation of mRNA translation. Nature *485*, 109-113. 10.1038/nature11083.

S9. Ottone, C., Krusche, B., Whitby, A., Clements, M., Quadrato, G., Pitulescu, M. E., Adams, R. H., and Parrinello, S. (2014). Direct cell–cell contact with the vascular niche maintains quiescent neural stem cells. Nature Cell Biology *16*, 1045-1056. 10.1038/ncb3045.

S10. Timaru-Kast, R., Herbig, E. L., Luh, C., Engelhard, K., and Thal, S. C. (2015). Influence of Age on Cerebral Housekeeping Gene Expression for Normalization of Quantitative Polymerase Chain Reaction after Acute Brain Injury in Mice. Journal of Neurotrauma *32*, 1777-1788. 10.1089/neu.2014.3784.
