# Supplementary material for: Injury primes mutation-bearing astrocytes for dedifferentiation in later life
Source: Curr Biol. 2023 Mar 27;33(6):1082–1098.e8. doi: 10.1016/j.cub.2023.02.013 (PMC10615847; doi:10.1016/j.cub.2023.02.013)
Supplement: Document S2. Article plus supplemental information [file mmc2.pdf]

Article

Current Biology

Injury primes mutation-bearing astrocytes for dedifferentiation in later life

Graphical abstract

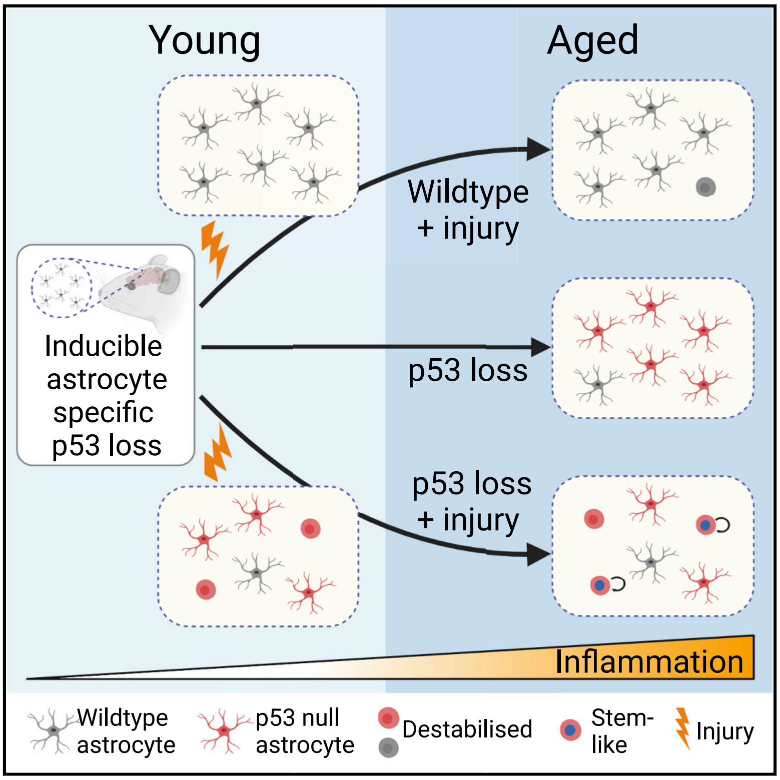

Authors

Holly Simpson Ragdale,
Melanie Clements, Wenhao Tang, ...,
Antonella Riccio, Samuel Marguerat,
Simona Parrinello

Correspondence

s.marguerat@ucl.ac.uk (S.M.),
s.parrinello@ucl.ac.uk (S.P.)

In brief

Simpson Ragdale et al. show that p53 inactivation, a common event in glioma, remains silent in healthy cortical astrocytes but leads to dedifferentiation to a neural stem-cell-like state upon injury. This results from p53 loss sensitizing astrocytes to neuroinflammation, which is induced by early-life injury and potentiated by aging.

Highlights

- The tumor suppressor p53 restricts injury-induced plasticity of cortical astrocytes
- p53 loss destabilizes astrocyte identity in the context of injury in early life
- Increased neuroinflammation at the injury site drives dedifferentiation upon aging
- EGFR activation by injury signals mediates dedifferentiation downstream of p53 loss

Simpson Ragdale et al., 2023, Current Biology *33*, 1082–1098
March 27, 2023 © 2023 The Authors. Published by Elsevier Inc.
https://doi.org/10.1016/j.cub.2023.02.013

CellPress
OPEN ACCESS

Current Biology

Article

Injury primes mutation-bearing astrocytes for dedifferentiation in later life

Holly Simpson Ragdale,[1] Melanie Clements,[1,11] Wenhao Tang,[2,3,10,11] Elitza Deltcheva,[4] Catia Andreassi,[5] Alvina G. Lai,[6] Wai Hoong Chang,[6] Maria Pandrea,[1] Ivan Andrew,[2,3] Laurence Game,[2,3] Imran Uddin,[7,8] Michael Ellis,[1] Tariq Enver,[4] Antonella Riccio,[5] Samuel Marguerat,[2,3,9,*] and Simona Parrinello[1,12,13,*]
[1]Samantha Dickson Brain Cancer Unit, UCL Cancer Institute, University College London, London WC1E 6DD, UK
[2]MRC London Institute of Medical Sciences, Du Cane Road, London W12 0NN, UK
[3]Institute of Clinical Sciences, Faculty of Medicine, Imperial College London, Du Cane Road, London W12 0NN, UK
[4]UCL Cancer Institute, University College London, London WC1E 6DD, UK
[5]UCL Laboratory for Molecular Cell Biology, University College London, London WC1E 6BT, UK
[6]Institute of Health Informatics, University College London, London NW1 2DA, UK
[7]CRUK City of London Centre Single Cell Genomics Facility, UCL Cancer Institute, University College London, London WC1E 6DD, UK
[8]Genomics Translational Technology Platform, UCL Cancer Institute, University College London, London WC1E 6DD, UK
[9]Present address: UCL Cancer Institute, University College London, London WC1E 6DD, UK
[10]Present address: Department of Mathematics, Faculty of Natural Sciences, Imperial College, London SW7 2AZ, UK
[11]These authors contributed equally
[12]Twitter: @Parrinello_Lab
[13]Lead contact
*Correspondence: s.marguerat@ucl.ac.uk (S.M.), s.parrinello@ucl.ac.uk (S.P.)
https://doi.org/10.1016/j.cub.2023.02.013

SUMMARY

Despite their latent neurogenic potential, most normal parenchymal astrocytes fail to dedifferentiate to neural stem cells in response to injury. In contrast, aberrant lineage plasticity is a hallmark of gliomas, and this suggests that tumor suppressors may constrain astrocyte dedifferentiation. Here, we show that p53, one of the most commonly inactivated tumor suppressors in glioma, is a gatekeeper of astrocyte fate. In the context of stab-wound injury, p53 loss destabilized the identity of astrocytes, priming them to dedifferentiate in later life. This resulted from persistent and age-exacerbated neuroinflammation at the injury site and EGFR activation in periwound astrocytes. Mechanistically, dedifferentiation was driven by the synergistic upregulation of mTOR signaling downstream of p53 loss and EGFR, which reinstates stemness programs via increased translation of neurodevelopmental transcription factors. Thus, our findings suggest that first-hit mutations remove the barriers to injury-induced dedifferentiation by sensitizing somatic cells to inflammatory signals, with implications for tumorigenesis.

INTRODUCTION

Parenchymal astrocytes retain a latent neurogenic program, which can be reactivated by brain injury.[1–5] Striatal astrocytes were shown to convert to cells with neural stem cell properties (NSC-like cells) and generate new neurons in response to excitotoxic damage or stroke, in a Notch-dependent manner.[2–4] Consistently, the deletion of *Rpbj-k* elicited astrocyte neurogenesis following cortical stab-wound injury.[1] However, in the wild-type brain, the majority of astrocyte subtypes remain within their lineage upon injury. This includes cortical astrocytes, which upregulate stemness markers, but fail to dedifferentiate *in vivo*, despite doing so when dissociated and cultured *in vitro*.[5,6]

In contrast, the astrocyte lineage barrier is fully subverted in tumorigenesis. Inactivation of the tumor suppressor protein p53 and overexpression of oncogenic H-RasV12 dedifferentiated cortical astrocytes to tumor-initiating glioma stem cells.[7] Furthermore, within established gliomas, differentiation of astrocyte-like cells is partial and unstable.[8,9] However, whether glioma-relevant genes play a role in controlling astrocyte plasticity in the normal brain remains unclear.

It is also poorly understood how the disruption of these genes may intersect with the dedifferentiation-promoting injury microenvironment. This is of relevance to glioma, as injury programs have emerged as major players in disease progression and recurrence, which develops within the inflammatory post-treatment microenvironment.[10–12] In this context, p53 is a particularly interesting candidate as it inhibits somatic cell reprogramming[13–15] and is one of the most commonly inactivated tumor suppressors in glioma.[16] In the adult subventricular zone (SVZ) neurogenic niche, p53 modulates neurogenesis by suppressing the proliferation of transit-amplifying progenitors and their differentiation to neuroblasts.[17–19] Furthermore, our analysis of the Zamboni et al. dataset[1] comparing wild-type astrocytes from healthy or stab-wound-injured cortices revealed a significant enrichment of p53 signatures in injured astrocytes (Figure S1A). A parallel enrichment was also observed in reactive human astrocytes in the Li et al. dataset[20] (Figure S1B). Together, this

 © 2023 The Authors. Published by Elsevier Inc.
This is an open access article under the CC BY license (http://creativecommons.org/licenses/by/4.0/).

CellPress
OPEN ACCESS

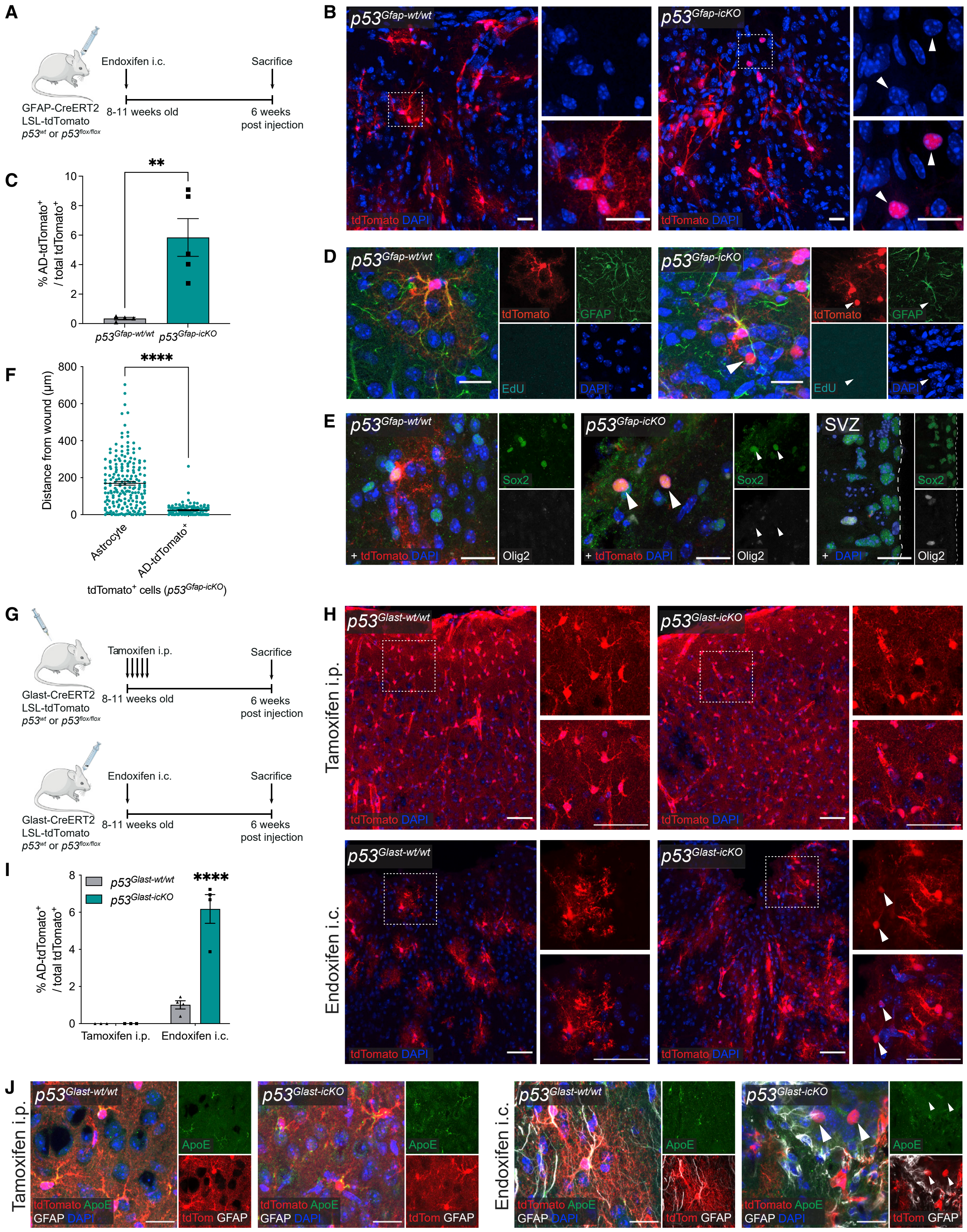

(legend on next page)

evidence suggests that p53 may function as a barrier to injury-induced astrocyte dedifferentiation.

In this study, we explored the role of p53 in the response of cortical astrocytes to stab-wound injury in young adulthood and the long-term impact of its deletion on astrocyte fate.

RESULTS

Acute p53 loss synergizes with injury signals to downregulate astrocyte lineage identity *in vivo*

To assess the impact of *p53* loss on adult cortical astrocytes in the context of injury, we generated a conditional and inducible mouse model driven by glial fibrillary acidic protein (GFAP), which is lowly or not expressed in most healthy cortical astrocytes, but upregulated upon injury during reactive astrogliosis.[21–23] GFAP-CreERT2 mice were crossed to a conditional *p53* knockout line ($p53^{flox/flox}$) alongside a tdTomato fluorescent reporter line (Rosa26:CAG-LoxP-STOP-LoxP-tdTomato or LSL-tdTomato) to generate $p53^{Gfap\text{-}icKO}$ and recombination was induced in adult animals by direct stereotactic injection of endoxifen, an active metabolite of tamoxifen, into the cortex[23–26] (Figure 1A). This enabled concomitant *p53* deletion and fate mapping of recombined cells and their progeny. It also ensured selective recombination of peri-injury cortical astrocytes in the absence of confounding recombination of SVZ progenitor cells (Figure S1C), which could migrate to the cortex and differentiate to astroglia *in situ*.[27] The stab-wound injury caused by the needle itself was used as an injury model, as reported.[10] $p53^{wt/wt}$ mice crossed to GFAP-CreERT2 and LSL-tdTomato ($p53^{Gfap\text{-}wt/wt}$) lines served as controls. 6 weeks after injection, a time at which astrocyte proliferation subsides and the injury response reaches a chronic remodeling phase,[28] animals were sacrificed and tdTomato$^+$ astrocytes assessed by immunostaining (Figure 1A). The majority of tdTomato$^+$ cells retained the characteristic branched-astrocyte morphology in both $p53^{Gfap\text{-}wt/wt}$ and $p53^{Gfap\text{-}icKO}$ mice (Figure 1B). However, in $p53^{Gfap\text{-}icKO}$ animals a significant proportion of recombined cells retracted their processes and became rounded (astrocyte-derived tdTomato$^+$ cells or AD-tdTomato$^+$) (Figures 1B and 1C). Importantly, immunostaining for RFP and cleaved caspase-3, respectively, confirmed that this phenotype was not due to loss of tdTomato fluorescence in the astrocyte processes (Figure S1D), nor was it due to cells dying (Figure S1E), but reflected a true shape change. AD-tdTomato$^+$ cells were also not ectopically recombined oligodendrocyte progenitor cells (OPCs), as they did not co-express Olig2 and the OPC marker PDGFR-alpha (Figure S1F).

To determine whether this morphological change was accompanied by a fate change, we examined the expression of differentiation and stemness markers. AD-tdTomato$^+$ cells had lost the mature astrocyte markers GFAP (Figure 1D) and ApoE (Figure S1G), but retained lineage markers Sox2 (Figure 1E) and nuclear factor I A (NFIA) (Figure S1G). Furthermore, AD-tdTomato$^+$ cells remained negative for the stemness marker Olig2 and for Ascl1, a key marker of early astrocyte neurogenesis[2,3] (Figures 1E and S1H), did not re-enter the cell cycle (Figures 1D and S1I) or acquire expression of the neuroblast marker doublecortin (DCX) (Figure S1H). Together, these results suggest that p53 loss destabilizes astrocyte identity following injury, but is insufficient to induce dedifferentiation or initiate neurogenesis at this early time point.

AD-tdTomato$^+$ cells were found almost exclusively in the immediate vicinity of the wound (<100 μm), suggesting a dependency on injury signals, the levels of which are highest within this area[28] (Figure 1F). To test this directly and rule out a general role in the maintenance of astrocyte fate, we examined effects of acute p53 loss on adult astrocytes in the intact cortex. To this end, we used a Glast-CreERT2 driver, which enables more efficient and widespread targeting of cortical astrocytes than the GFAP-CreERT2 line following systemic tamoxifen administration.[23,29] Glast-CreERT2 mice and LSL-tdTomato were crossed to $p53^{flox/flox}$ or $p53^{wt/wt}$ animals to generate $p53^{Glast\text{-}icKO}$ and $p53^{Glast\text{-}wt/wt}$, respectively, and recombination was induced by intraperitoneal (i.p.) tamoxifen injections in adult animals (Figure 1G, top). In parallel, $p53^{Glast\text{-}icKO}$ and $p53^{Glast\text{-}wt/wt}$ were subjected to stereotactic intracortical endoxifen injections as described for the GFAP-CreERT2 line, to enable side-by-side

Figure 1. p53 loss and injury synergize to downregulate astrocyte lineage identity *in vivo*

(A) Schematic of experimental outline. Endoxifen was injected intracranially (i.c.) into the cortex of 8- to 11-week-old *GFAP-CreERT2; LSL-tdTomato;* $p53^{wt/wt}$ ($p53^{Gfap\text{-}wt/wt}$) or *GFAP-CreERT2; LSL-tdTomato;* $p53^{flox/flox}$ ($p53^{Gfap\text{-}icKO}$) mice. 6 weeks postinjection, mice were sacrificed, and fate-mapped astrocytes (tdTomato$^+$) were assessed by immunostaining.

(B) Representative images of tdTomato$^+$ lineage-traced cells in indicated genotypes. Astrocyte-derived rounded cells without processes (AD-tdTomato$^+$) are indicated by white arrowheads. Scale bars, 20 μm.

(C) Quantification of AD-tdTomato$^+$ cells as a percentage of total tdTomato$^+$ cells. (Mean ± SEM; $p53^{Gfap\text{-}wt/wt}$, n = 4; $p53^{Gfap\text{-}icKO}$, n = 5. **p < 0.01, unpaired two-tailed t test.)

(D and E) Representative images of GFAP and EdU staining (D) and Olig2 and Sox2 staining (E). AD-tdTomato$^+$ cells are negative for GFAP, EdU, and Olig2 but are Sox2$^+$. In (E), subventricular zone (SVZ) is included as positive staining control. Scale bars, 20 μm.

(F) Quantification of the perpendicular distance of cells from the injury site in n = 4 $p53^{Gfap\text{-}icKO}$ animals. (Mean ± SEM; ****p < 0.0001, unpaired two-tailed t test.)

(G) Schematic of experimental outline. Tamoxifen was injected intraperitoneally (i.p.) or endoxifen was injected i.c. into the cortex of 8- to 11-week-old *Glast-CreERT2; LSL-tdTomato;* $p53^{wt/wt}$ ($p53^{Glast\text{-}wt/wt}$) or *Glast-CreERT2; LSL-tdTomato;* $p53^{flox/flox}$ ($p53^{Glast\text{-}icKO}$) mice. 6 weeks postinjection, mice were sacrificed, and fate-mapped astrocytes (tdTomato$^+$) were assessed by immunostaining.

(H) Representative images of tdTomato$^+$ lineage-traced cells in indicated conditions. White dashed box indicates location of higher magnification image (right). Representative AD-tdTomato$^+$ cells are indicated by white arrowheads. Scale bars, 50 μm.

(I) Quantification of AD-tdTomato$^+$ cells as a percentage of the total tdTomato$^+$ cells. (Mean ± SEM; tamoxifen i.p., n = 3, and endoxifen i.c., n = 4 for each genotype. ****p < 0.0001 $p53^{Glast\text{-}icKO}$ endoxifen i.c. compared with each other condition. Comparisons of all other conditions were not significant [p > 0.05], two-way ANOVA with Tukey's multiple-comparisons test.)

(J) Representative images of GFAP and ApoE staining in indicated conditions. Representative AD-tdTomato$^+$ cells are indicated by white arrowheads. Scale bars, 20 μm.

See also Figure S1.

CellPress
OPEN ACCESS

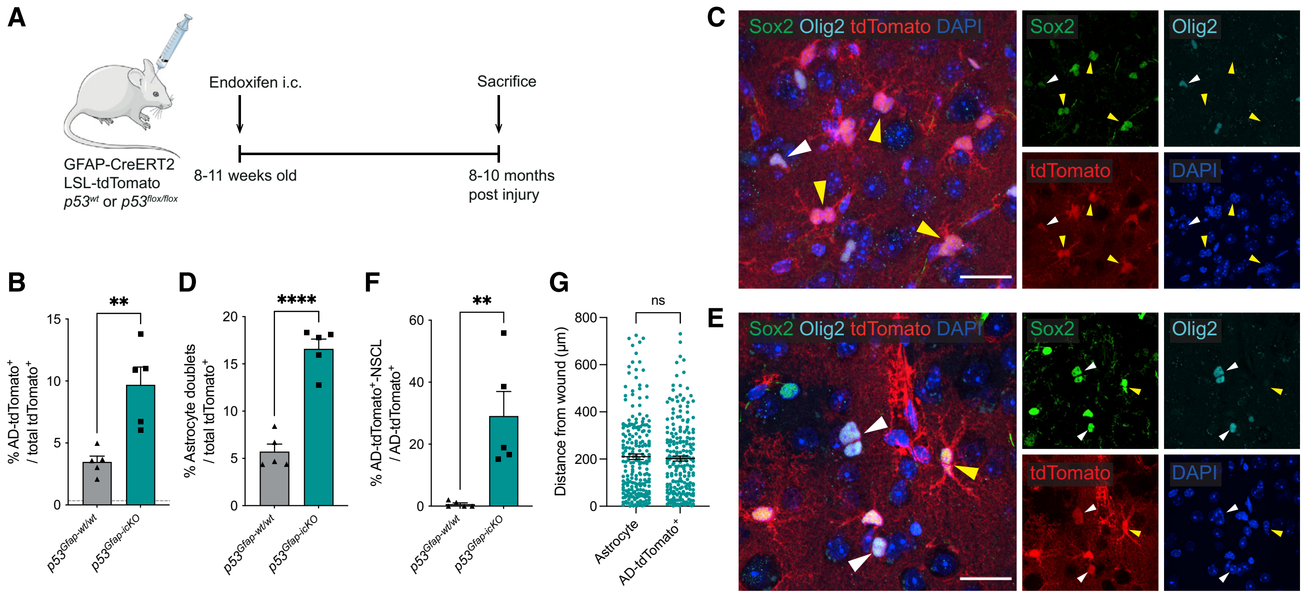

Figure 2. The aging microenvironment drives dedifferentiation of injury-primed p53-deficient cortical astrocytes *in vivo*

(A) Schematic of experimental outline. Endoxifen was injected intracranially into the cortex of 8- to 11-week-old *GFAP-CreERT2; LSL-tdTomato; p53*$^{wt/wt}$ (*p53*$^{Gfap\text{-}wt/wt}$) or *GFAP-CreERT2; LSL-tdTomato; p53*$^{flox/flox}$ (*p53*$^{Gfap\text{-}icKO}$). 8–10 months postinjection, mice were sacrificed, and fate-mapped astrocytes (tdTomato^{+}) were assessed by immunostaining.

(B) Quantification of AD-tdTomato^{+} cells as a percentage of total tdTomato^{+} cells. (Mean + SEM; n = 5 per condition, **$p < 0.01$, unpaired two-tailed t test.) Dashed line indicates basal levels of AD-tdTomato^{+} cells in young injured *p53*$^{Gfap\text{-}wt/wt}$ mice from Figure 1C.

(C) Representative image of astrocytic tdTomato^{+} cell doublets (yellow arrowheads) and an Olig2^{+}/Sox2^{+} AD-tdTomato^{+}-NSCL cell (white arrowhead) in *p53*$^{Gfap\text{-}icKO}$. Scale bars, 25 µm.

(D) Quantification of tdTomato^{+} astrocyte doublets as a percentage of total tdTomato^{+} cells. (Mean + SEM; n = 5 per condition, ****$p < 0.0001$, unpaired two-tailed t test.)

(E) Representative image of Olig2^{+}/Sox2^{+} AD-tdTomato^{+}-NSCL cells (white arrowheads) in *p53*$^{Gfap\text{-}icKO}$, including a cell doublet indicative of recent division. Scale bars, 25 µm.

(F) Quantification of AD-tdTomato^{+}-NSCL cells as a percentage of AD-tdTomato^{+} cells. (Mean + SEM; n = 5 per condition; **$p < 0.01$, unpaired two-tailed t test.)

(G) Quantification of the perpendicular distance of cells from the injury site in n = 5 aged injured *p53*$^{Gfap\text{-}icKO}$ animals. (Mean ± SEM; ns, not significant; Mann-Whitney test.) See also Figure S2.

comparison of p53 loss in healthy and injured brains (Figure 1G, bottom). As in *p53*$^{Gfap\text{-}icKO}$ mice, we observed significant numbers of rounded AD-tdTomato^{+} that downregulated mature astrocyte markers in endoxifen-injected *p53*$^{Glast\text{-}icKO}$ but not *p53*$^{Glast\text{-}wt/wt}$ animals (Figures 1H–1J). This indicates that the destabilization of astrocyte fate is a general response of p53-deficient astrocytes to injury. In contrast, in uninjured, tamoxifen-injected mice of both genotypes, astrocyte morphology and identity remained unaltered (Figure 1H–1J). Thus, p53 restricts astrocyte plasticity selectively following injury and its loss synergizes with injury signals to destabilize astrocyte fate.

The aging cortical microenvironment induces injury-primed p53 null astrocytes to dedifferentiate *in vivo*

We reasoned that although at 6 weeks postinjury p53-deficient astrocytes underwent a modest change in identity, their fate may be further affected over a longer time period. We therefore examined the cortices of *p53*$^{Gfap\text{-}wt/wt}$ and *p53*$^{Gfap\text{-}icKO}$ mice at 8–10 months following endoxifen intracortical injections, corresponding to mice of >1 year of age (Figure 2A). Interestingly, we found an increase in the proportion of AD-tdTomato^{+} cells in both genotypes, suggesting that the aging tissue microenvironment is sufficient to drive partial loss of astrocyte identity following early-life injury, even without *p53* deletion (Figure 2B). However, *p53*$^{Gfap\text{-}icKO}$ injured cortices displayed an increase in astrocyte doublets, which is indicative of recent astrocyte proliferation (Figures 2C and 2D) and, more strikingly, uniquely contained a subset of AD-tdTomato^{+} cells that co-expressed the stemness and transit-amplifying progenitor markers Sox2, Olig2, and Ascl1, indicating that they may have acquired stem-like characteristics (Figures 2C, 2E, 2F, and S2A). These cells were also lowly proliferative, in that they occasionally appeared as doublets (Figure 2E) or were positive for the proliferation marker Ki67 (Figure S2B). Remarkably, we also detected rare Sox2^{-}, Olig2^{-}, and NeuN^{+} tdTomato^{+} cells with a neuronal morphology selectively in the aged *p53*$^{Gfap\text{-}icKO}$-injured brains, suggesting that some p53-deficient astrocytes may acquire neurogenic potential (Figures S2C and S2D). These data indicate that upon aging, p53-deficient AD-tdTomato^{+} progress to a dedifferentiated neural stem-cell-like state (AD-tdTomato^{+}-NSCL cells).

Importantly, these phenotypes were not a general consequence of aging, as we did not detect AD-tdTomato^{+} cells, astrocyte doublets or AD-tdTomato^{+}-NSCL cells in aged-matched uninjured *p53*$^{Glast\text{-}wt/wt}$ or *p53*$^{Glast\text{-}icKO}$ animals 8–10 months following systemic tamoxifen administration

(Figures S2E–S2H). Thus, injury in early life primes p53-deficient astrocytes to dedifferentiate upon aging.

Exacerbated neuroinflammation at the wound site underlies age-dependent dedifferentiation of p53-deficient astrocytes following early-life injury

We next sought to understand what properties of the aging brain microenvironment may render it permissive to full dedifferentiation of AD-tdTomato$^+$ cells. Increased neuroinflammation is a hallmark of aging.[30,31] Furthermore, the positioning of AD-tdTomato$^+$ cells near the needle tract at 6 weeks postinjury is suggestive of a role for inflammatory signals (Figure 1F) and qPCR analysis of young and old cortical astrocytes revealed a small but significant increase in p53 levels, which is indicative of shared inflammation-related mechanisms between injury and aging responses (Figure S3A). We therefore hypothesized that later-life dedifferentiation may be driven by age-dependent neuroinflammation.

To test this, we first examined resident microglia, which play a major role in age-dependent neuroinflammation.[30] Iba-1 and CD68 immunofluorescence analysis was carried out in intact or injured cortices at 6 weeks and 10 months after injury to examine microglia morphology and activation, respectively (Figures S3B–S3D). As expected, this revealed that microglia acquired morphological changes associated with activation and priming, such as deramification and shortening of their cellular processes, in the peri-injury regions (both proximal and distal to the needle tract) of both young and old brains, as well as in intact old brains (Figures S3B and S3C). However, CD68 levels differed between groups, with microglia in the proximal wound displaying the highest CD68 expression regardless of age, and a selective increase in expression in the distal wound region of old animals (Figure S3D). These observations suggest that the inflammatory response to a stab-wound injury in early life exacerbates age-dependent neuroinflammation, resulting in more extensive and pronounced inflammation in the periwound region than either injury in the young brain or aging alone. To understand how these changes might contribute to later-life dedifferentiation, we examined the distribution of AD-tdTomato$^+$ cells at the wound site in aged animals. Unlike in young mice, where destabilization of astrocyte fate only occurred adjacent to the needle tract (Figure 1F), at 8–10 months postinjection we found AD-tdTomato$^+$ cells in both proximal and distal wound regions (Figure 2G). Together, these findings are consistent with microglia-mediated inflammation promoting dedifferentiation of p53-deficient astrocytes.

To test this hypothesis, we next performed gain-of-function studies. Young *p53*$^{Gfap\text{-}wt/wt}$ and *p53*$^{Gfap\text{-}icKO}$ mice were subjected to stab-wound injury alongside intraperitoneal (i.p.) administration of lipopolysaccharide (LPS), which increases neuroinflammation through activation of microglia.[32–34] LPS was administered at day 11 postinjury and animals were sacrificed 3 days later to achieve maximal amplification of the endogenous inflammatory response (which peaks between 1 and 2 weeks postinjury) while avoiding systemic LPS-induced toxicity[28] (Figure 3A). As expected, this treatment regime resulted in microglia activation throughout the brain,[33,34] which was further increased at the injury site and greater than matched PBS-treated controls (Figure S3E). We found that the combination of stab-wound injury, p53 loss, and microglia-mediated inflammation was sufficient to recapitulate the phenotype of injured and aged *p53*$^{Gfap\text{-}icKO}$ brains, leading to the formation of AD-tdTomato$^+$ cells, a subset of which were lowly proliferative AD-tdTomato$^+$-NSCL cells (Figures 3B–3E and S3F–S3H). As in aged brains, LPS also induced AD-tdTomato$^+$ cells in wild-type brains, confirming that partial loss of astrocyte identity in the absence of mutations depends on inflammation (Figure 3C). Together, these findings suggest that early-life injury synergizes with age-related inflammation to convert the cortical microenvironment to a permissive milieu for the dedifferentiation of p53-deficient astrocytes.

Dedifferentiation of p53-deficient astrocytes is EGFR dependent

We next sought to identify the signaling pathways that respond to inflammatory signals to drive dedifferentiation of p53 deficient astrocytes. Epidermal growth factor receptor (EGFR) signaling underpins the activation of quiescent NSCs[35] and increases neurogenesis of *Rbpj-k*-deficient striatal astrocytes.[3] Furthermore, EGF levels increase in the peri-injury brain microenvironment.[21] We therefore examined the role of EGFR through a series of complementary experiments. First, we compared expression levels of EGFR ligands in injured and contralateral cortices from young brains, as well as in uninjured young and old cortices (Figures S3I and S3J) and found a significant increase relative to controls in both. A similar increase was also observed in published datasets of microglia activation following LPS treatment[36] or upon aging,[37] consistent with EGFR signaling potentially responding to neuroinflammation (Figures S3K and S3L). Second, we carried out gain- and loss-of-function experiments. Young adult *p53*$^{Gfap\text{-}wt/wt}$ and *p53*$^{Gfap\text{-}icKO}$ mice were injected intracranially (i.c.) with endoxifen and treated with osimertinib, a selective EGFR inhibitor, or vehicle control for 2 weeks (Figure 4A), which effectively dampened EGFR signaling in the periwound region (Figures S4A and S4B). We found that osimertinib reduced the percentage of AD-tdTomato$^+$ cells in young adult *p53*$^{Gfap\text{-}icKO}$ mice to *p53*$^{Gfap\text{-}wt/wt}$ control levels, indicating that increased EGFR signaling is necessary for the destabilization of astrocyte identity following p53 loss (Figures 4B and 4C). To test if EGFR activation is also sufficient, we infused recombinant EGF, or saline control, to the injury site of young adult mice through osmotic minipumps implanted at the time of intracortical endoxifen administration (Figure 4D). Strikingly, EGF infusion phenocopied p53 effects observed in both young and aged brains: in *p53*$^{Gfap\text{-}wt/wt}$ mice, it induced the formation of AD-tdTomato$^+$ cells to an extent similar to p53 deletion in young injured brains (Figures 4E and 4F); in *p53*$^{Gfap\text{-}icKO}$ mice it increased the overall proportion of AD-tdTomato$^+$ cells and, as in the aged and LPS-treated brain, selectively led to their progression to AD-tdTomato$^+$-NSCL cells, as judged by the presence of doublets, Ki67$^+$ and Sox2$^+$/Olig2$^+$/Ascl1$^+$ AD-tdTomato$^+$ cells (Figures 4E–4G and S4C–S4H). Together, these experiments indicate that p53 phenotypes are EGFR dependent.

scRNA-seq reveals temporal progression of p53-induced astrocyte dedifferentiation

To explore the mechanisms by which p53 loss and EGFR signaling synergize to drive dedifferentiation, we turned to an

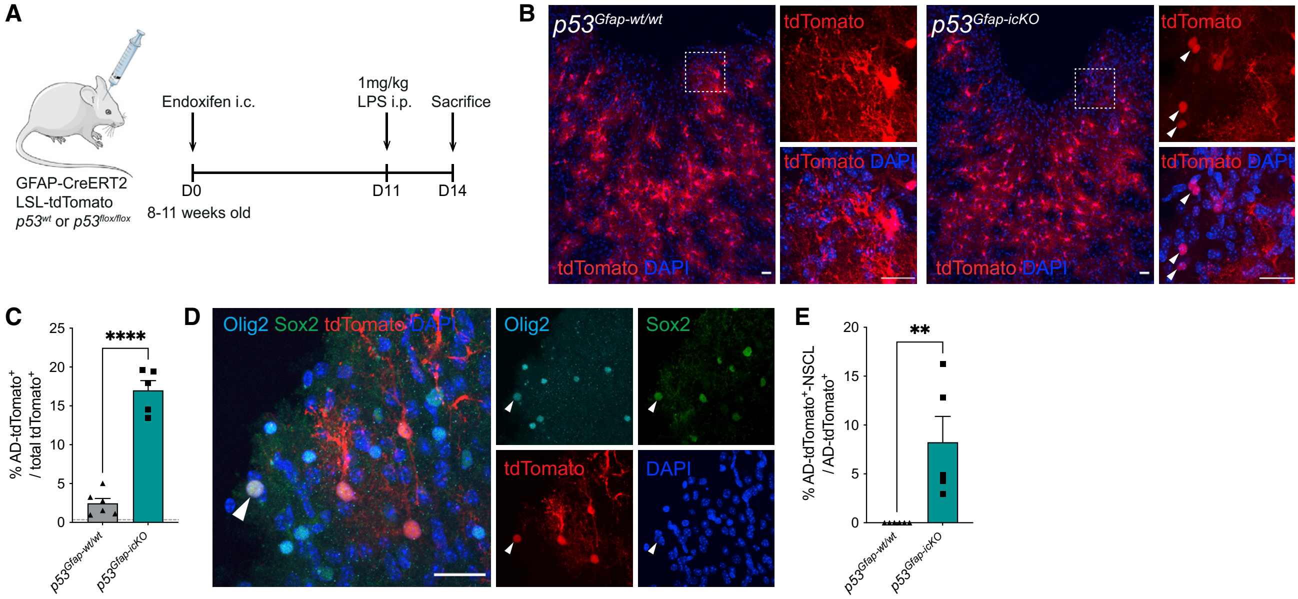

Figure 3. Increased neuroinflammation underlies age-dependent dedifferentiation of p53-deficient astrocytes following early-life injury

(A) Schematic of experimental outline. Endoxifen was injected intracranially into the cortex of 8- to 11-week-old *GFAP-CreERT2; LSL-tdTomato; p53*$^{wt/wt}$ (*p53*$^{Gfap\text{-}wt/wt}$) or *GFAP-CreERT2; LSL-tdTomato; p53*$^{flox/flox}$ (*p53*$^{Gfap\text{-}icKO}$) mice. 11 days later, lipopolysaccharide (LPS, 1 mg/kg) was injected intraperitoneally (i.p.) to induce peripheral inflammation and microglial activation. 3 days later, mice were sacrificed, and fate-mapped astrocytes (tdTomato$^+$) were assessed by immunostaining.

(B) Representative images of tdTomato$^+$ lineage-traced cells in indicated genotypes after endoxifen and LPS injection. Astrocyte-derived rounded cells without processes (AD-tdTomato$^+$) are indicated by white arrowheads. Scale bars, 25 μm.

(C) Quantification of AD-tdTomato$^+$ cells as a percentage of total tdTomato$^+$ cells. (Mean + SEM; LPS *p53*$^{Gfap\text{-}wt/wt}$, n = 6; LPS *p53*$^{Gfap\text{-}icKO}$, n = 5; ****$p < 0.0001$, unpaired two-tailed t test.) Dashed line indicates basal levels of AD-tdTomato$^+$ cells in young injured *p53*$^{Gfap\text{-}wt/wt}$ mice from Figure 1C.

(D) Representative image of AD-tdTomato$^+$-NSCL cells (white arrowheads) in LPS injected *p53*$^{Gfap\text{-}icKO}$. Scale bars, 25 μm.

(E) Quantification of AD-tdTomato$^+$-NSCL cells as a percentage of AD-tdTomato$^+$ cells. (Mean + SEM; LPS *p53*$^{Gfap\text{-}wt/wt}$, n = 6; LPS *p53*$^{Gfap\text{-}icKO}$, n = 5; **$p < 0.01$, unpaired two-tailed t test.) See also Figure S3.

established and more tractable *in vitro* model. Primary astrocytes were purified from the cortices of postnatal day 3 *p53*$^{flox/flox}$; LSL-tdTomato mice,[38] recombined *in vitro* using GFAP-Cre or empty vector adenoviruses to generate *p53*$^{-/-}$ and *p53*$^{+/+}$ astrocytes, respectively, and cultured for 7 days in EGF-containing NSC media to mimic the injured microenvironment. Parallel cultures were maintained in bone morphogenetic protein 4 (BMP4) to mimic the prodifferentiative signals of the intact cortex[39] (Figure S4I, top). Clonogenic assays were then used to assess dedifferentiation. Similar to our observations *in vivo*, p53 deletion did not dedifferentiate astrocytes in BMP4, nor did EGF treatment of p53 wild-type cells (Figure S4J). However, p53 deletion combined with EGF-induced dedifferentiation to cultured NSC-like cells, as indicated by the appearance of discreet colonies of small tdTomato$^+$ cells, which acquired Ascl1 and Olig2 protein expression, strongly increased Sox2 protein levels, and were highly proliferative (Figures S4J–S4N). Furthermore, replating of EGF-treated *p53*$^{-/-}$, but not *p53*$^{+/+}$, cultures in suspension formed neurospheres capable of self-renewal and multilineage differentiation (Figures S4O–S4Q). To determine whether these findings are relevant to human astrocytes, we repeated these experiments in primary human astrocytes (and human NSC-derived astrocytes) using lentiviral shRNA constructs to acutely downregulate p53 (Figures S4R–S4X). We found all mouse phenotypes to be reproduced in human cells, with p53-knockdown (but not control-vector transduced) astrocytes dedifferentiating to NSC-like cells in the presence of EGF and retaining their astrocytic phenotype in BMP4. Thus, our *in vitro* models recapitulate *in vivo* phenotypes to a large extent and support the notion that p53 deletion synergizes with EGFR signaling to dedifferentiate cortical astrocytes to NSC-like cells.

Having validated our *in vitro* system, we next sought to define the cellular mechanisms that underlie p53-dependent astrocyte dedifferentiation using scRNA-seq. To enable analysis of cells at various stages of dedifferentiation, we prepared primary postnatal cortical astrocytes from *p53*$^{Gfap\text{-}icKO}$ mice and induced recombination *in vitro* using 4-hydroxytamoxifen (4OHT) for 5 days in the presence of EGF (Figures 5A and S4I, bottom), which leads to heterogeneous recombination times. As expected, this model also resulted in astrocyte dedifferentiation to NSC-like cells selectively in p53 deleted astrocytes exposed to EGF and was therefore equivalent to the adenoviral system (Figures S4J and S5A).

We analyzed whole transcriptomes of 11,635 single cells derived from two independent cultures on two separate 10x Chromium runs. As these did not show strong batch effects or differences in tdTomato recombination frequencies, they were pooled for further analysis (Figures S5B–S5D); t-distributed stochastic neighbor embedding (t-SNE) analysis of the combined datasets revealed three distinct cell populations that were further

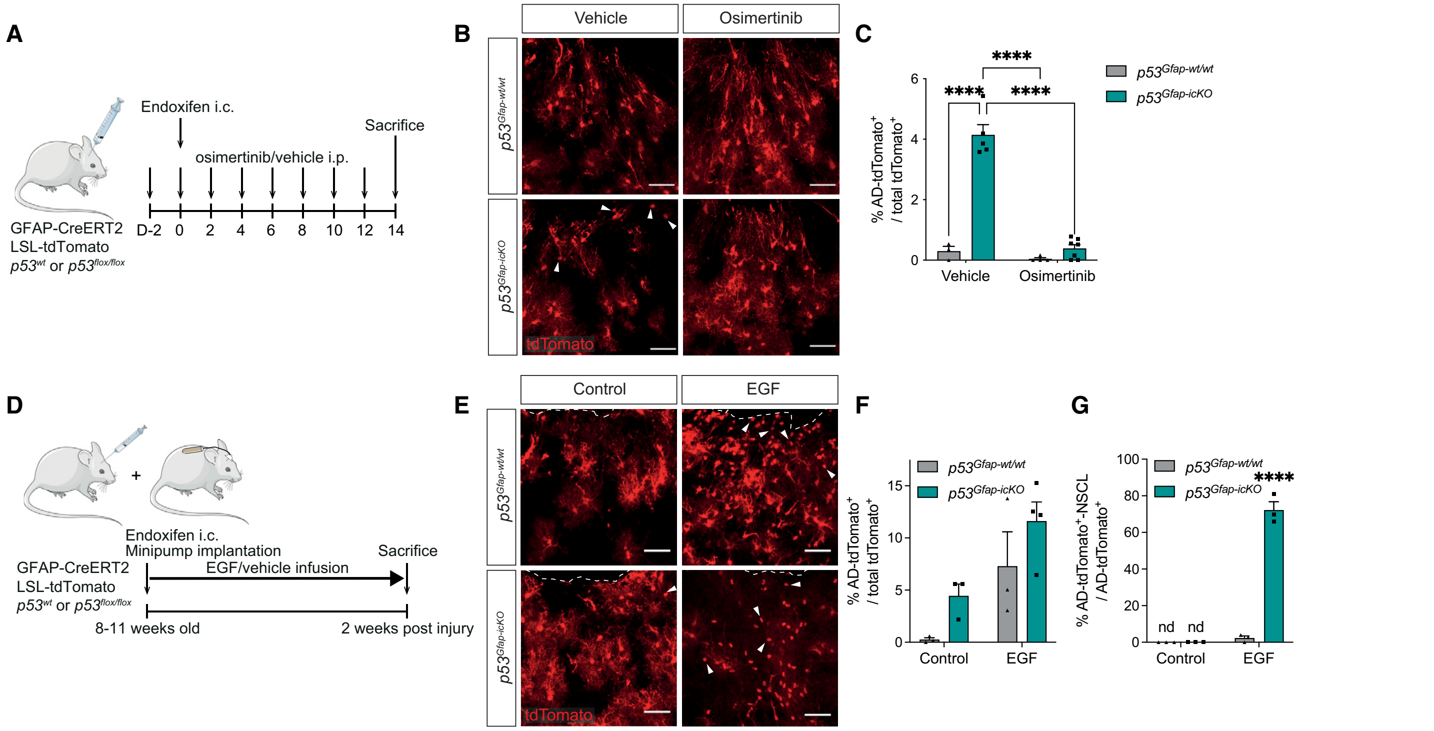

Figure 4. Dedifferentiation of p53-deficient astrocytes is EGFR dependent

(A) Schematic of experimental outline. *GFAP-CreERT2; LSL-tdTomato; p53*$^{wt/wt}$ (*p53*$^{Gfap\text{-}wt/wt}$) or *GFAP-CreERT2; LSL-tdTomato; p53*$^{flox/flox}$ (*p53*$^{Gfap\text{-}icKO}$) mice were injected intracranially (i.c.) with endoxifen into the cortex to induce astrocyte-specific *p53* recombination and tdTomato labeling. Mice were also i.p. injected with osimertinib or vehicle control 2 days before i.c. injection, and every other day thereafter. Mice were sacrificed 2 weeks post i.c. injection and fate-mapped (tdTomato$^+$) astrocytes were assessed.

(B) Representative images of tdTomato$^+$ fate-mapped cells in osimertinib experiment. White arrowheads indicate AD-tdTomato$^+$ cells. Scale bars, 50 μm.

(C) Quantification of percentage of AD-tdTomato$^+$ cells as a percentage of the total tdTomato$^+$ cells. *p53*$^{Gfap\text{-}wt/wt}$ vehicle, n = 3; *p53*$^{Gfap\text{-}icKO}$ vehicle, n = 5; *p53*$^{Gfap\text{-}wt/wt}$ osimertinib, n = 4; *p53*$^{Gfap\text{-}icKO}$ osimertinib, n = 7.

(D) Schematic of experimental outline. 8- to 11-week-old *GFAP-CreERT2; LSL-tdTomato; p53*$^{wt/wt}$ (*p53*$^{Gfap\text{-}wt/wt}$) or *GFAP-CreERT2; LSL-tdTomato; p53*$^{flox/flox}$ (*p53*$^{Gfap\text{-}icKO}$) mice were stereotactically injected i.c. with endoxifen into the cortex to induce astrocyte-specific *p53* recombination and tdTomato labeling. Infusion cannula attached to an osmotic minipump continuously infused EGF or vehicle control into injection site. After 2 weeks of infusion, mice were sacrificed, and fate-mapped (tdTomato$^+$) astrocytes were assessed by immunostaining.

(E) Representative image of tdTomato$^+$ cell morphology. White arrowheads indicate representative AD-tdTomato$^+$ cells. Scale bars, 50 μm.

(F) Quantification of AD-tdTomato$^+$ cells as a percentage of the total tdTomato$^+$ cells. (n = 3 per condition except *p53*$^{Gfap\text{-}icKO}$ EGF, n = 4.)

(G) Quantification of AD-tdTomato$^+$-NSCL cells as a percentage of AD-tdTomato$^+$ cells. (n = 3 per condition). (For all graphs, mean + SEM; nd, not detected; ns, not significant; ****p < 0.0001, two-way ANOVA with Tukey's multiple-comparisons test.)

See also Figures S3 and S4.

divided into 9 clusters (Figure 5B). After removing low-quality cells (cluster 8, Figures 5C and S5E), cells likely contaminating CD52$^+$ microglia (cluster 9, Figures 5C and S5E), PCP4$^+$ OPCs (cluster 7, Figures 5C, S5E, and S5F) and unrecombined cells, based on cell-type marker expression or absence of tdTomato reads, respectively, 7,898 cells remained. These encompassed clusters 1–6, on which the rest of the analysis was focused. Clusters 1–4 expressed astrocyte markers to varying degrees, including *Apoe* and *Gfap* (Figures 5C and S5E), likely reflecting the heterogeneity of cortical astrocytes *in vivo*.[40,41] Cluster 4 displayed a signature related to cytoskeleton remodeling, including *Actb* upregulation (Figures 5C and S5E). Interestingly, this cluster still retained expression of the p53 target *Cdkn1a*, suggesting that this signature may be a p53-independent response, possibly linked to mitogenic stimulation (Figure 5C). Consistently, exposure to EGF and FGF induced a dramatic change in the actin cytoskeleton of both *p53*$^{+/+}$ and *p53*$^{-/-}$ cultures previously maintained in BMP4 (Figure S5G). Clusters 5 and 6 had signatures of ribosomal biogenesis and stemness (including *Ascl1* and *Olig2* expression), with cluster 6 also displaying a strong increase in proliferation signatures (Figures 5E and S5E). These two clusters therefore likely contained dedifferentiating astrocytes and astrocyte-derived NSC-like cells. Importantly, both expressed low levels of *Cdkn1a* (Figure 5C), which was accompanied by the strongest downregulation of p53 pathway signatures in cluster 6 (Figure 5E), in line with our *in vivo* findings supporting a role for p53 as a gatekeeper of astrocytic fate.

We then asked whether an ordered dedifferentiation path could be found in our data using SPRING[42] (Figures 5D and S5J), pseudotime (monocle3, Figure S5H) and velocity (Figures S5I and S5J). All approaches converged toward a

CellPress
OPEN ACCESS

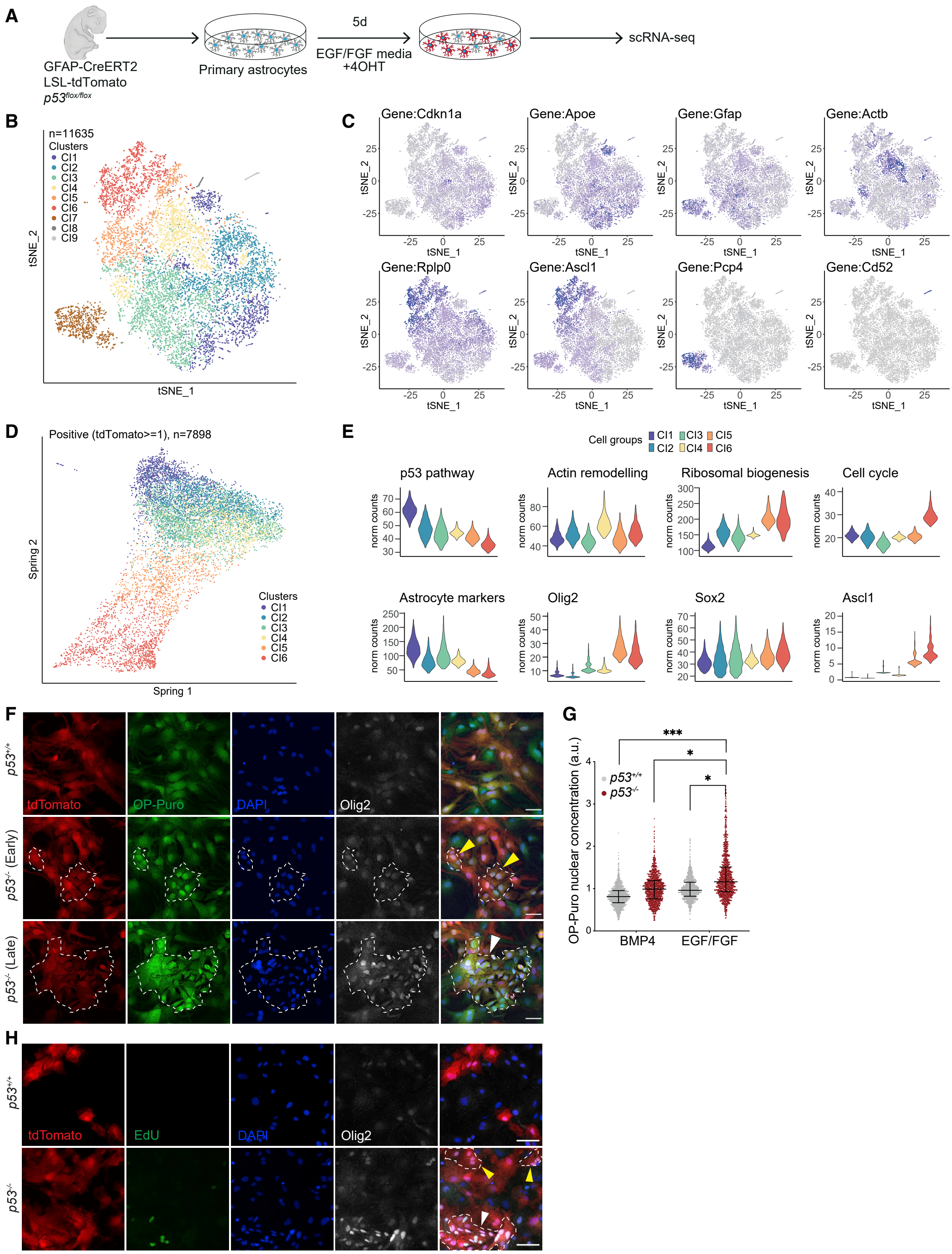

(legend on next page)

model where astrocytes exist in four interconnected states with no clear directionality, likely due to the aforementioned heterogeneity of the starting cultures (clusters 1–4). From these states, dedifferentiating cells downregulated astrocyte markers while concomitantly increasing expression of ribosomal genes and stemness markers (cluster 5), prior to progressing to a highly proliferative NSC-like state reminiscent of normal active NSC or transit-amplifying progenitors (cluster 6) (Figure 5E). These observations suggest that p53-dependent astrocyte dedifferentiation closely mirrors the progression of normal quiescent SVZ NSCs to an active state, which includes an intermediate state of primed quiescence, in which cells increase protein synthesis in preparation for neurogenesis.[43,44] To confirm this timeline and that ribosomal gene signatures reflect changes in protein synthesis, we performed O-propargyl-puromycin (OP-puro) incorporation experiments (Figures 5F and 5G). High Olig2 expression was used as a selective marker of clusters 5 and 6, and an 5-ethynyl-2'-deoxyuridine (EdU) pulse prior to analysis enabled correlation to proliferation rate (Figure 5H). We detected two types of Olig2^{high} colonies in the cultures, which consisted of small nonastrocytic tdTomato^{+} cells: smaller "early" colonies of 3–15 cells, which were largely EdU^{-} and larger, more compact "late" colonies of >15 cells that incorporated EdU (Figures 5F–5H). Importantly, both types of colonies displayed elevated OP-puro, regardless of their proliferative status. These results are consistent with the pseudotime analysis, placing increased protein synthesis upstream of cell proliferation during dedifferentiation. The ability to dedifferentiate to NSC-like cells through these mechanisms was not unique to postnatal astrocytes, which retain stemness potential.[45] Rather, it was also conserved in adult cells, as judged by scRNA-seq analysis of adult cortical-astrocyte preparations *in vitro* and, crucially, from tdTomato^{+} cells acutely FACS-sorted from injured and aged brains of *p53*$^{Gfap\text{-}wt/wt}$ and *p53*$^{Gfap\text{-}icKO}$ mice (Figure S5K). Indeed, integration of postnatal and adult scRNA-seq datasets using the Harmony batch-correction algorithm[46] identified dedifferentiated cells that clustered with postnatal astrocyte-derived NSC-like cells in both *in vitro* and *in vivo* adult populations (Figure S5K). *In vitro*, NSC-like cells (cluster A2) formed via downregulation of astrocyte markers and increasing ribosomal biogenesis prior to cell-cycle re-entry (Figures S5L and S5M), and *in vivo* were observed selectively in *p53*$^{Gfap\text{-}icKO}$ (Figures S5K*iv* and S5K*v*) at a frequency consistent with our immunohistochemistry analyses (see Figures 2B and 2F). Thus, p53 loss reactivates a neurogenic response in cortical astrocytes that mimics the transition from quiescent to activated SVZ NSCs.

p53 loss drives dedifferentiation through derepression of mTOR-mediated translation of stemness transcription factors

Mammalian target of rapamycin (mTOR) is a central regulator of protein synthesis, and SVZ NSCs display elevated levels of mTOR-dependent translation.[47] We therefore asked whether the detected increase in translation upon p53 loss was mTOR-dependent. Immunofluorescence analysis of *p53*$^{+/+}$ and *p53*$^{-/-}$ astrocyte cultures revealed a stark increase in phospho-S6 ribosomal protein (pS6RP), which was specific to *p53*$^{-/-}$ NSC-like colonies and correlated with increased OP-puro incorporation (Figures 6A, 6B, and S6A). A similar increase in pS6RP was seen *in vivo* in AD-tdTomato^{+}-NSCL cells, but not in tdTomato^{+} astrocytes (Figure S6B). Consistent with this, clusters 5 and 6 in postnatal, and corresponding clusters A3 and A2 in adult astrocytes, displayed elevated expression of mTOR and Myc signatures in the scRNA-seq datasets (Figure S6C). Interestingly, the increase in mTOR signaling in *p53*$^{-/-}$ was greater in EGF, likely due to more robust activation of the pathway[48] (Figure 6B). Furthermore, acute mTOR inhibition by Torin completely blocked dedifferentiation of *p53*$^{-/-}$ astrocytes (Figures 6C and S6D). p53 is known to suppress mTOR signaling through direct transcriptional upregulation of Phlda3,[49] Sestrin1, and Sestrin2.[50] To determine whether any of these genes are direct p53 targets in astrocytes, we performed ChIP sequencing analysis in wild-type cells and correlated it to our scRNA-seq data. p53 bound the promoter regions of both *Phlda3* and *Sesn2* (Figure 6D), with both transcripts becoming strongly downregulated upon p53 loss in postnatal and adult astrocytes (Figures 6E and S6E). This is consistent with p53 loss derepressing mTOR signaling downstream of EGF to increase protein synthesis at the onset of dedifferentiation.

In adult neurogenesis, translational downregulation of Sox2 transcript by mTOR is necessary for stemness exit and neuronal differentiation.[47] Selectivity is provided by a pyrimidine-rich motif (PRM), which is present in the 5′ UTR of the

Figure 5. scRNA-seq reveals temporal progression of astrocyte dedifferentiation *in vitro*

(A) Schematic of experimental outline. Primary cortical astrocytes were isolated from postnatal P3 *GFAP-CreERT2; LSL-tdTomato; p53*$^{flox/flox}$ (*p53*$^{Gfap\text{-}icKO}$) pups. Following immunopanning, astrocytes were incubated with 4-hydroxytamoxifen (4OHT) in EGF/FGF media for 5 days to induce *p53* and tdTomato allele recombination and astrocyte dedifferentiation, before collection for 10x Genomics scRNA-seq.

(B) t-SNE plot of all cells.

(C) Expression of key upregulated gene per cluster shown on t-SNE plots as in (B).

(D) SPRING analysis of cells dynamic trajectories, after filtering to remove contaminant clusters (Cl7–9) and tdTomato negative cells (tdTomato reads = 0).

(E) Violin plots of pathway analysis (top) or indicated genes (bottom) in ordered clusters.

(F) Representative images of OP-puro labeling and Olig2 staining in EGF/FGF media. Shown is the increased protein synthesis (OP-puro labeling) and Olig2 expression in both early (yellow arrowhead, smaller cell clusters) and late (white arrowhead, larger cell clusters) dedifferentiated colonies in *p53*$^{-/-}$ cultures, defined by change in cell size and morphology relative to astrocytes. Colonies are marked by dashed lines. Scale bars, 50 μm.

(G) Quantification of OP-puro nuclear protein staining intensity from (F). Each point represents an individual cell. Line represents median with interquartile range. (n = 3 independent experiments; *$p < 0.05$, ***$p < 0.001$; two-way ANOVA with Tukey's multiple-comparisons test on average intensity per condition per replicate.)

(H) Representative images of Olig2 staining and EdU incorporation in *p53*$^{+/+}$ and *p53*$^{-/-}$ cultures in EGF/FGF media. Late Olig2^{+} clusters (white arrowheads) are highly proliferative and correspond to Cl6 cells. Scale bars, 50 μm.

See also Figures S4 and S5.

CellPress
OPEN ACCESS

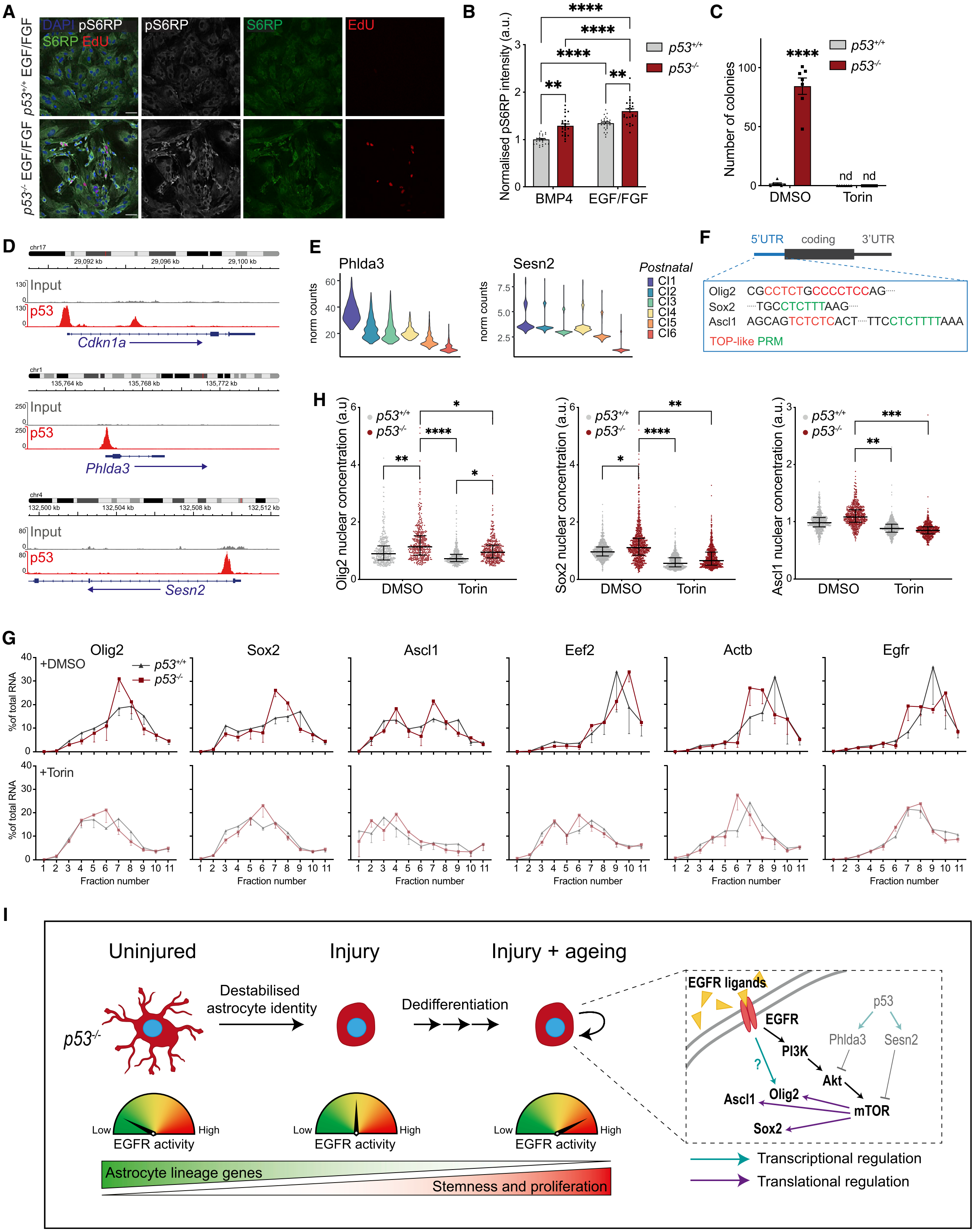

(legend on next page)

Sox2 transcript.[47] In our system, Sox2 protein, but not mRNA, levels increased significantly upon dedifferentiation (Figures 5E, S4M, and S4N). In addition, Ascl1 and Olig2 protein levels were much greater in late colonies than early ones, while mRNA levels were similar (Figures 5E, 5F, 5H, and S4M). We therefore hypothesized that mTOR-dependent translation of neurodevelopmental transcripts might underlie dedifferentiation by resetting astrocytes to a stem-like state. To test this hypothesis, we first examined the 5′ UTRs of *Ascl1* and *Olig2* mRNAs and found that both contained terminal oligopyrimidine (TOP)-like sequences,[51] with *Ascl1* also containing a PRM region[47] (Figure 6F). Second, we performed polysome fractionation experiments to measure ribosomal loading of *Sox2*, *Ascl1*, and *Olig2* transcripts in $p53^{+/+}$ and $p53^{-/-}$ astrocytes before and after Torin treatment (Figures 6G and S6F). This revealed a slightly higher proportion of *Olig2*, *Sox2*, and *Ascl1* transcripts in the polysomal fractions (fraction number >6) of $p53^{-/-}$ relative to $p53^{+/+}$ astrocytes, consistent with p53 loss promoting their translation. Furthermore, Torin treatment strongly repressed translation of all three transcripts, as judged by a pronounced shift toward the light and nontranslating polysome fractions, which was similar to that of the well-described mTOR-regulated transcript *Eef2*.[51] In contrast, only a modest shift was observed in transcripts devoid of PRM and TOP motifs (*Actb* and *Egfr*), which was likely due to global decrease in protein translation upon mTOR blockade, rather than a transcript-selective effect.[51] Finally, Torin treatment resulted in a parallel reduction in the levels of all three proteins in both wild-type and p53-deleted cells (Figure 6H). We conclude that p53 loss drives astrocyte dedifferentiation through downregulation of mTOR inhibitor genes, which leads to derepression of mTOR signaling downstream of EGFR. In turn, increased mTOR activity induces translation of stemness transcription factors to reinstate an NSC-like state (Figure 6I).

DISCUSSION

Cortical astrocytes are notoriously refractory to dedifferentiative cues *in vivo*, but the underlying mechanisms have remained unclear.[1,5,6,52] Here we identified p53 as a key mediator. This is consistent with emerging functions of p53 in controlling cellular plasticity, for example, in cancer initiation, reprogramming, and epithelial-to-mesenchymal transition.[53–57] However, while most of those functions have been attributed to cell-intrinsic p53 effects, our data reveal an unexpected dependence on microenvironmental cues. We found that p53 loss remained silent in healthy astrocytes and only following an injury and accompanying increase in neuroinflammation did astrocytes dedifferentiate. Thus, in analogy to cancer initiation, inflammatory cues then act as a "second hit" that enables the dedifferentiation of cells that have been made competent to respond to these cues by p53 loss.

Our findings are in line with increasing evidence that healthy tissues bear a variety of driver mutations, which are tolerated within a normal tissue microenvironment.[58–62] They further suggest that it is a shift toward an inflammatory tissue microenvironment that enables mutated cells to express their malignant potential, with dedifferentiation to NSC-like cells and increased fate plasticity being key responses.

Notably, we identified EGFR as main receiver of dedifferentiative inflammatory signals. EGFR has long been known to play a fundamental role in controlling NSC decisions and suppressing differentiation.[35,48,63] It is also a commonly amplified gene in glioblastoma.[64] Our findings indicate that EGFR signaling acts as a rheostat in the control of astrocyte fate, whereby high signaling is required for dedifferentiation. Such levels cannot readily be achieved in wild-type cells, which converted to AD-tdTomato$^+$ cells but failed to dedifferentiate even upon EGF infusion or injury-induced neuroinflammation. However, our data suggest that p53 loss lowers the threshold at which astrocytes respond

Figure 6. Increased mTOR activity downstream of p53 loss and EGFR activation leads to increased translation of stemness transcription factors

(A) Representative images of phospho-S6 ribosomal protein (pS6RP) and S6RP staining in astrocytes cultured in EGF/FGF. Colonies of dedifferentiated cells have higher pS6RP staining. Scale bars, 50 μm.

(B) Quantification of pS6RP intensity normalized to total S6RP shown in (A). Pixel intensity of cellular cytoplasmic mask per whole field of view was quantified for n = 3 independent experiments. Each point represents a single field of view. (Mean ± SEM; a.u., arbitrary units; $^{**}p < 0.01$, $^{****}p < 0.0001$; two-way ANOVA with Tukey's multiple-comparisons test.)

(C) Quantification of number of dedifferentiated colonies visualized by crystal violet staining for $p53^{+/+}$ or $p53^{-/-}$ astrocytes cultured in EGF/FGF media supplemented with mTOR inhibitor, Torin, or vehicle control (DMSO). Torin treatment abolishes astrocyte dedifferentiation. (Mean ± SEM; nd, not detected; $^{****}p < 0.0001$; two-way ANOVA with Tukey's multiple-comparisons test.

(D) ChIP sequencing of wild-type primary astrocytes shows p53 binding to canonical target *Cdkn1a* (positive control) and negative regulators of the mTOR pathway *Phlda3* and *Sesn2*.

(E) Violin plots of indicated genes in the ordered clusters from postnatal *in vitro* scRNA-seq data. Expression of *Phlda3* and *Sesn2* decreases sequentially with p53 pathway inactivation (as in Figure 5E).

(F) Terminal oligopyrimidine (TOP)-like motif or pyrimidine-rich motif (PRM) identified in 5′ UTRs of indicated genes.

(G) Distribution of mRNAs of indicated genes across the gradient fractions of the polysome profiles of $p53^{+/+}$ or $p53^{-/-}$ astrocytes cultured in EGF/FGF media in the absence (+DMSO) or presence of Torin (+Torin). (Mean ± SEM; n = 3 independent experiments).

(H) Quantification of Olig2, Sox2, and Ascl1 nuclear protein staining intensity. Each point represents an individual cell. Line represents median with interquartile range. (n = 3 independent experiments; $^{*}p < 0.05$, $^{**}p < 0.01$, $^{***}p < 0.001$, $^{****}p < 0.0001$; two-way ANOVA with Tukey's multiple comparisons test on average intensity per condition per replicate.)

(I) Schematic of proposed mechanisms of astrocyte dedifferentiation. In uninjured brains where EGFR activity is low, derepression of mTOR upon p53 loss has no effect. Upon injury, the progressive increase in levels of EGFR ligands first destabilizes the identity of p53 null astrocytes in young adults and, in later life, causes their full dedifferentiation. This occurs through synergistic activation of mTOR signaling, which is switched on by EGFR and derepressed by p53 loss and drives translation of neurodevelopmental transcription factors to restore stemness programs in astrocytes. Teal and purple arrows indicate transcriptional and translational regulation, respectively.

See also Figure S6.

to EGFR signaling, enabling their full conversion to the AD-tdTomato^{+}-NSCL state within the injury microenvironment (Figure 6I). Thus, cell-extrinsic EGFR activation and cell-intrinsic p53 loss together create the "perfect storm" for subverting the astrocyte lineage barrier. Interestingly, although EGFR amplification and p53 alterations are both frequent mutations in glioblastoma, they rarely co-occur in patients.[16,64] While more studies will be required to mechanistically unravel this mutual exclusivity, our findings may provide a partial explanation as they predict that p53 inactivation would be sufficient to hyperactivate wild-type EGFR, thus eliminating any selective pressure for EGFR mutations.

At the molecular level, we found the synergy between EGFR signaling and p53 loss to coalesce upon mTOR, which our *in vitro* data suggest reset stemness by inducing translation of neurodevelopmental transcription factors. It was recently shown that the differentiation of active NSCs to neuroblasts requires mTOR-dependent translational repression of stemness transcription factors, including Sox2.[47] Thus, p53-dependent dedifferentiation of astrocytes may represent a reversal of normal developmental programs. Further conservation with normal neurogenesis was observed at the transcriptional level, where dedifferentiation programs closely mirrored the activation of quiescent NSCs to a proliferative state.[43] This is consistent with recent findings and further underscores the notion that parenchymal astrocytes may represent dormant NSCs.[1,3]

Our findings have implications for brain cancer etiology. The "cell of origin" in glioma remains a highly controversial and debated topic. As astrocytes rarely divide in the healthy brain, they have been considered unlikely candidates.[65,66] Our data challenge this view, as they suggest that mutations acquired during periods of active astrocyte proliferation, such as development or reactive astrogliosis, may lay silent in the healthy brain only to be activated by injury. In this model, dedifferentiated p53-deficient astrocyte-derived NSC-like cells that re-enter the cell cycle might then acquire additional mutations and initiate tumorigenesis. Indeed, seminal studies examining the role of p53 in SVZ neurogenesis demonstrated that, while p53 loss alone is not tumor-initiating, it does sensitize NSCs to mutagens and enables spontaneous acquisition of additional mutations, which ultimately lead to glioma formation.[19,67–70] Importantly, the earliest detectable glioma cells in those models expressed the same molecular markers (Ascl1/Olig2) that we detected in AD-tdTomato^{+}-NSCLs.[69,70] To examine the possibility that astrocyte-derived *p53*$^{-/-}$-NSCL cells may become prone to transformation as their *p53*$^{-/-}$ NSC counterparts, we used soft-agar assays comparing *p53*$^{-/-}$ and *p53*$^{+/+}$ astrocytes cultured in BMP4 or EGF over time. We found that dedifferentiated *p53*$^{-/-}$ NSCL cells in EGF acquired anchorage independence (a readout of tumorigenicity) after 9 passages, whereas *p53*$^{-/-}$ astrocytes in BMP4 and *p53*$^{+/+}$ astrocytes in either culture condition, did not (Figures S6G and S6H), providing proof of principle that p53-dependent dedifferentiation increases the probability of astrocyte transformation via a dedifferentiation step. Future studies should examine the *in vivo* tumorigenic potential of AD-tdTomato^{+}-NSCL after longer-term proliferation and exposure to injury signals, for example, following repeated trauma or chronic inflammation.[71]

A link between brain injury and human glioma has long been proposed and is supported by some studies.[72–77] To probe this more directly, we used electronic health records and propensity-score matching to estimate the impact of head injury on brain cancer in patients. We identified 23,232 patients who had a diagnosis of head injury and 194,942 matched controls using propensity-score matching, matched by year of birth, sex, and socioeconomic deprivation. The known glioma risk factors, i.e., phakomatoses and radiation exposure, were used as positive, whereas diabetes and fatty-liver disease were used as negative controls. As expected, the risk of developing brain cancer was the highest in patients with phakomatoses (hazard ratio [HR] 26.18, CI: 14.56–47.05, $p < 0.0001$), followed by those exposed to radiation (HR 6.36, CI: 5.15–7.85, $p < 0.0001$), whereas no association was observed with fatty-liver disease or diabetes (HR 0.83; CI: 0.64–1.07, $p = 0.151$). Strikingly, patients who experienced a head injury were also approximately 3.8 times more likely to develop a brain cancer later in life, which is indicative of injury being a significant risk factor for brain cancer (HR 3.78, CI: 2.85–5.02, $p < 0.0001$) (Figure S6I).

It is tempting to speculate that the mechanisms identified here may be relevant to glioblastoma recurrence, which in the majority of patients occurs within 2 cm of the resection cavity.[78,79] The exposure of residual tumor cells to injury signals induced by surgery, chemotherapy, and radiotherapy in this region[12,80,81] may convert residual p53-deficient tumor cells to a cancer-initiating, NSC-like state.

Finally, our observations have implications for age-related pathology. They suggest that the inflammation caused by injury in early life, even if relatively mild, has a long-term impact on tissue function and ultimately exacerbates age-dependent neuroinflammation in the periwound area.[82–84] It is of note that p53 levels in healthy astrocytes increased upon aging. Future studies should explore whether this reduces astrocyte plasticity or injury responses with age.

In conclusion, our study shows that early-life injury primes mutated cells to dedifferentiation in later life by sensitizing them to age-dependent inflammatory cues and suggests that unraveling the complex interactions between injury and aging may unlock new strategies for cancer prevention.

STAR★METHODS

Detailed methods are provided in the online version of this paper and include the following:

- KEY RESOURCES TABLE
- RESOURCE AVAILABILITY
  - Lead contact
  - Materials availability
  - Data and code availability
- EXPERIMENTAL MODEL AND SUBJECT DETAILS
  - Mice
  - Primary postnatal astrocyte isolation and culture
  - Primary adult astrocyte isolation and culture
  - Human astrocyte culture
- METHOD DETAILS
  - *In vivo* administrations and surgeries
  - p53 recombination in primary astrocytes
  - Clonogenic assays

- ○ Neurosphere assay and re-differentiation
- ○ Astrocyte soft agar experiments
- ○ Immunofluorescence and Immunohistochemistry
- ○ Image analysis and processing
- ○ scRNA-sequencing of *in vitro* cells
- ○ Acute purification of tdTomato$^+$ cells from aged injured mice and Smartseq3 scRNA-seq
- ○ Preprocessing and normalization of scRNA-seq data
- ○ ChIP-sequencing and analysis
- ○ Polysome fractionation
- ○ RNA isolation and qRT-PCR
- ○ Re-analysis of published RNA-seq datasets
- ○ Estimating the impact of head injury and additional risk factors on brain cancer outcomes using electronic health records
- QUANTIFICATION AND STATISTICAL ANALYSIS

SUPPLEMENTAL INFORMATION

Supplemental information can be found online at https://doi.org/10.1016/j.cub.2023.02.013.

ACKNOWLEDGMENTS

This work was funded by Cancer Research UK (A21203; S.P., H.S.R., and M.E.), NIHR Biomedical Research Centre (NIHR-INF-0389, M.C.), the Medical Research Council (MC_UP_A652_1002; [S.P. and H.S.R.], MC-A658-5TY20; [S.M.]), Wellcome Trust Investigator awards 103717/Z/14/Z and 217213/Z/19/Z (to A.R. and C.A.), and the MRC LMCB Core Grant MC/U12266B (to A.R.). This work was supported by The Oli Hilsdon Foundation through The Brain Tumour Charity, grant number (GN-000595), in connection with the program "mapping the spatio-temporal heterogeneity of glioblastoma invasion" (W.T. and M.C.). It used the computing resources of the UK Medical Bioinformatics partnership (UK MED-BIO; aggregation, integration, visualization, and analysis of large, complex data), which is supported by the Medical Research Council and Imperial College High Performance Computing Service. The authors acknowledge the use of the UCL Legion and Myriad High Performance Computing Facility (Legion@UCL and Myriad@UCL), and associated support services, in the completion of this work. E.D. is funded by CCLG/LPT (CCLGA 2021 17 Enver). We thank Cure Cancer@UCL and Mrs. Sandra Hamilton for the generous support. A.G.L. is supported by funding from the Wellcome Trust (204841/Z/16/Z), National Institute for Health Research University College London Hospitals Biomedical Research Centre (BRC714/HI/RW/101440), NIHR Great Ormond Street Hospital Biomedical Research Centre (19RX02), and Academy of Medical Sciences (SBF006\1084). Work at the CRUK City of London Centre Single Cell Genomics Facility and Cancer Institute Genomics Translational Technology Platform was supported by the CRUK City of London Centre Award (C7893/A26233). We thank M. Götz for Glast-CreERT2 mice, J. Manji and T. Lenn for help with microscopy, Y. Guo and G. Morrow for FACS, L. Conde for help with bioinformatics, A. McLatchie and P. Dhami for sequencing advice, H. Bulstrode for human NSCs, J. Gil for reagents and access to laboratory facilities, and F. Guillemot for the Ascl1 antibody. Parts of schematic figures (Figures 1A, 1G, 2A, 3A, 4A, 4D, 5A, 6I, and S4I) were adapted from artwork from Servier Medical Art, provided by Servier, licensed under a Creative Commons Attribution 3.0 Unported License (https://creativecommons.org/licenses/by/3.0/). The graphical abstract figure was created with BioRender.com.

AUTHOR CONTRIBUTIONS

Conceptualization, S.M. and S.P.; methodology, H.S.R. and S.P.; investigation, H.S.R., M.C., E.D., C.A., M.P., I.A., I.U., M.E., and S.P.; formal analysis, H.S.R., W.T., A.G.L., W.H.C., and S.M.; resources, E.D., C.A., L.G., T.E., A.R., and S.M.; writing—original draft, H.S.R. and S.P.; writing—review and editing, H.S.R., M.C., S.M., and S.P.; visualization, H.S.R., W.T., S.M., and S.P.; supervision, T.E., A.R., S.M., and S.P.; funding acquisition, L.G., T.E., A.R., S.M., and S.P.

DECLARATION OF INTERESTS

The authors declare no competing interests.

INCLUSION AND DIVERSITY

We support inclusive, diverse, and equitable conduct of research.

Received: April 8, 2022
Revised: December 8, 2022
Accepted: February 2, 2023
Published: February 24, 2023

REFERENCES

1. Zamboni, M., Llorens-Bobadilla, E., Magnusson, J.P., and Frisén, J. (2020). A widespread neurogenic potential of neocortical astrocytes is induced by injury. Cell Stem Cell *27*, 605–617.e5. https://doi.org/10.1016/j.stem.2020.07.006.
2. Magnusson, J.P., Göritz, C., Tatarishvili, J., Dias, D.O., Smith, E.M.K., Lindvall, O., Kokaia, Z., and Frisén, J. (2014). A latent neurogenic program in astrocytes regulated by Notch signaling in the mouse. Science *346*, 237–241. https://doi.org/10.1126/science.346.6206.237.
3. Magnusson, J.P., Zamboni, M., Santopolo, G., Mold, J.E., Barrientos-Somarribas, M., Talavera-Lopez, C., Andersson, B., and Frisén, J. (2020). Activation of a neural stem cell transcriptional program in parenchymal astrocytes. eLife *9*, e59733. https://doi.org/10.7554/eLife.59733.
4. Nato, G., Caramello, A., Trova, S., Avataneo, V., Rolando, C., Taylor, V., Buffo, A., Peretto, P., and Luzzati, F. (2015). Striatal astrocytes produce neuroblasts in an excitotoxic model of Huntington's disease. Development *142*, 840–845. https://doi.org/10.1242/dev.116657.
5. Buffo, A., Rite, I., Tripathi, P., Lepier, A., Colak, D., Horn, A.P., Mori, T., and Götz, M. (2008). Origin and progeny of reactive gliosis: A source of multipotent cells in the injured brain. Proc. Natl. Acad. Sci. USA *105*, 3581–3586. https://doi.org/10.1073/pnas.0709002105.
6. Sirko, S., Behrendt, G., Johansson, P.A., Tripathi, P., Costa, M., Bek, S., Heinrich, C., Tiedt, S., Colak, D., Dichgans, M., et al. (2013). Reactive glia in the injured brain acquire stem cell properties in response to sonic hedgehog. [corrected]. Cell Stem Cell *12*, 426–439. https://doi.org/10.1016/j.stem.2013.01.019.
7. Friedmann-Morvinski, D., Bushong, E.A., Ke, E., Soda, Y., Marumoto, T., Singer, O., Ellisman, M.H., and Verma, I.M. (2012). Dedifferentiation of neurons and astrocytes by oncogenes can induce gliomas in mice. Science *338*, 1080–1084. https://doi.org/10.1126/science.1226929.
8. Neftel, C., Laffy, J., Filbin, M.G., Hara, T., Shore, M.E., Rahme, G.J., Richman, A.R., Silverbush, D., Shaw, M.L., Hebert, C.M., et al. (2019). An integrative model of cellular states, plasticity, and genetics for glioblastoma. Cell *178*, 835–849.e21. https://doi.org/10.1016/j.cell.2019.06.024.
9. Carén, H., Stricker, S.H., Bulstrode, H., Gagrica, S., Johnstone, E., Bartlett, T.E., Feber, A., Wilson, G., Teschendorff, A.E., Bertone, P., et al. (2015). Glioblastoma stem cells respond to differentiation cues but fail to undergo commitment and terminal cell-cycle arrest. Stem Cell Rep. *5*, 829–842. https://doi.org/10.1016/j.stemcr.2015.09.014.
10. Brooks, L.J., Clements, M.P., Burden, J.J., Kocher, D., Richards, L., Devesa, S.C., Zakka, L., Woodberry, M., Ellis, M., Jaunmuktane, Z., et al. (2021). The white matter is a pro-differentiative niche for glioblastoma. Nat. Commun. *12*, 2184. https://doi.org/10.1038/s41467-021-22225-w.
11. Richards, L.M., Whitley, O.K.N., MacLeod, G., Cavalli, F.M.G., Coutinho, F.J., Jaramillo, J.E., Svergun, N., Riverin, M., Croucher, D.C., Kushida, M., et al. (2021). Gradient of Developmental and Injury Response

CellPress
OPEN ACCESS

transcriptional states defines functional vulnerabilities underpinning glioblastoma heterogeneity. Nat. Cancer *2*, 157–173. https://doi.org/10.1038/s43018-020-00154-9.

12. Brooks, L.J., Simpson Ragdale, H., Hill, C.S., Clements, M., and Parrinello, S. (2022). Injury programs shape glioblastoma. Trends Neurosci. *45*, 865–876. https://doi.org/10.1016/j.tins.2022.08.006.

13. Hong, H., Takahashi, K., Ichisaka, T., Aoi, T., Kanagawa, O., Nakagawa, M., Okita, K., and Yamanaka, S. (2009). Suppression of induced pluripotent stem cell generation by the p53–p21 pathway. Nature *460*, 1132–1135. https://doi.org/10.1038/nature08235.

14. Lin, T., and Lin, Y. (2017). p53 switches off pluripotency on differentiation. Stem Cell Res. Ther. *8*, 44. https://doi.org/10.1186/s13287-017-0498-1.

15. Kawamura, T., Suzuki, J., Wang, Y.V., Menendez, S., Morera, L.B., Raya, A., Wahl, G.M., and Izpisúa Belmonte, J.C.I. (2009). Linking the p53 tumor suppressor pathway to somatic cell reprogramming. Nature *460*, 1140–1144. https://doi.org/10.1038/nature08311.

16. Brennan, C.W., Verhaak, R.G.W., McKenna, A., Campos, B., Noushmehr, H., Salama, S.R., Zheng, S., Chakravarty, D., Sanborn, J.Z., Berman, S.H., et al. (2013). The somatic genomic landscape of glioblastoma. Cell *155*, 462–477. https://doi.org/10.1016/j.cell.2013.09.034.

17. Liu, H., Jia, D., Li, A., Chau, J., He, D., Ruan, X., Liu, F., Li, J., He, L., and Li, B. (2013). p53 regulates neural stem cell proliferation and differentiation via BMP-Smad1 signaling and Id1. Stem Cells Dev. *22*, 913–927. https://doi.org/10.1089/scd.2012.0370.

18. Meletis, K., Wirta, V., Hede, S.M., Nistér, M., Lundeberg, J., and Frisén, J. (2006). p53 suppresses the self-renewal of adult neural stem cells. Development *133*, 363–369. https://doi.org/10.1242/dev.02208.

19. Gil-Perotin, S., Marin-Husstege, M., Li, J., Soriano-Navarro, M., Zindy, F., Roussel, M.F., Garcia-Verdugo, J.-M., and Casaccia-Bonnefil, P. (2006). Loss of p53 induces changes in the behavior of subventricular zone cells: implication for the genesis of glial tumors. J. Neurosci. *26*, 1107–1116. https://doi.org/10.1523/JNEUROSCI.3970-05.2006.

20. Li, J., Pan, L., Pembroke, W.G., Rexach, J.E., Godoy, M.I., Condro, M.C., Alvarado, A.G., Harteni, M., Chen, Y.-W., Stiles, L., et al. (2021). Conservation and divergence of vulnerability and responses to stressors between human and mouse astrocytes. Nat. Commun. *12*, 3958. https://doi.org/10.1038/s41467-021-24232-3.

21. Robel, S., Berninger, B., and Götz, M. (2011). The stem cell potential of glia: lessons from reactive gliosis. Nat. Rev. Neurosci. *12*, 88–104. https://doi.org/10.1038/nrn2978.

22. Sirko, S., Irmler, M., Gascón, S., Bek, S., Schneider, S., Dimou, L., Obermann, J., De Souza Paiva, D., Poirier, F., Beckers, J., et al. (2015). Astrocyte reactivity after brain injury-: the role of galectins 1 and 3. Glia *63*, 2340–2361. https://doi.org/10.1002/glia.22898.

23. Hirrlinger, P.G., Scheller, A., Braun, C., Hirrlinger, J., and Kirchhoff, F. (2006). Temporal control of gene recombination in astrocytes by transgenic expression of the tamoxifen-inducible DNA recombinase variant CreERT2. Glia *54*, 11–20. https://doi.org/10.1002/glia.20342.

24. Marino, S., Vooijs, M., van Der Gulden, H., Jonkers, J., and Berns, A. (2000). Induction of medulloblastomas in P53-null mutant mice by somatic inactivation of Rb in the external granular layer cells of the cerebellum. Genes Dev. *14*, 994–1004.

25. Madisen, L., Zwingman, T.A., Sunkin, S.M., Oh, S.W., Zariwala, H.A., Gu, H., Ng, L.L., Palmiter, R.D., Hawrylycz, M.J., Jones, A.R., et al. (2010). A robust and high-throughput Cre reporting and characterization system for the whole mouse brain. Nat. Neurosci. *13*, 133–140. https://doi.org/10.1038/nn.2467.

26. Benedykcinska, A., Ferreira, A., Lau, J., Broni, J., Richard-Loendt, A., Henriquez, N.V., and Brandner, S. (2015). Generation of brain tumors by Cre-mediated recombination of neural progenitors in situ with the tamoxifen metabolite endoxifen. Dis. Models Mech. *9*, 211–220. https://doi.org/10.1242/dmm.022715.

27. Faiz, M., Sachewsky, N., Gascón, S., Bang, K.W., Morshead, C.M., and Nagy, A. (2015). Adult neural stem cells from the subventricular zone give rise to reactive astrocytes in the cortex after stroke. Cell Stem Cell *17*, 624–634. https://doi.org/10.1016/j.stem.2015.08.002.

28. Burda, J.E., and Sofroniew, M.V. (2014). Reactive gliosis and the multicellular response to CNS damage and disease. Neuron *81*, 229–248. https://doi.org/10.1016/j.neuron.2013.12.034.

29. Mori, T., Tanaka, K., Buffo, A., Wurst, W., Kühn, R., and Götz, M. (2006). Inducible gene deletion in astroglia and radial glia-A valuable tool for functional and lineage analysis. Glia *54*, 21–34. https://doi.org/10.1002/glia.20350.

30. Norden, D.M., and Godbout, J.P. (2013). Review: Microglia of the aged brain: primed to be activated and resistant to regulation. Neuropathol. Appl. Neurobiol. *39*, 19–34. https://doi.org/10.1111/j.1365-2990.2012.01306.x.

31. Wyss-Coray, T. (2016). Aging, neurodegeneration and brain rejuvenation. Nature *539*, 180–186. https://doi.org/10.1038/nature20411.

32. Buttini, M., and Boddeke, H. (1995). Peripheral lipopolysaccharide stimulation induces interleukin-1β messenger RNA in rat brain microglial cells. Neuroscience *65*, 523–530. https://doi.org/10.1016/0306-4522(94)00525-A.

33. Hoogland, I.C.M., Houbolt, C., Van Westerloo, D.J., Van Gool, W.A., and Van De Beek, D. (2015). Systemic inflammation and microglial activation: systematic review of animal experiments. J. Neuroinflammation *12*, 114. https://doi.org/10.1186/s12974-015-0332-6.

34. Batista, C.R.A., Gomes, G.F., Candelario-Jalil, E., Fiebich, B.L., and De Oliveira, A.C.P. (2019). Lipopolysaccharide-induced neuroinflammation as a bridge to understand neurodegeneration. Int. J. Mol. Sci. *20*, 2293. https://doi.org/10.3390/ijms20092293.

35. Dulken, B.W., Leeman, D.S., Boutet, S.C., Hebestreit, K., and Brunet, A. (2017). Single-cell transcriptomic analysis defines heterogeneity and transcriptional dynamics in the adult neural stem cell lineage. Cell Rep. *18*, 777–790. https://doi.org/10.1016/j.celrep.2016.12.060.

36. Guttenplan, K.A., Weigel, M.K., Adler, D.I., Couthouis, J., Liddelow, S.A., Gitler, A.D., and Barres, B.A. (2020). Knockout of reactive astrocyte activating factors slows disease progression in an ALS mouse model. Nat. Commun. *11*, 3753. https://doi.org/10.1038/s41467-020-17514-9.

37. Gyoneva, S., Hosur, R., Gosselin, D., Zhang, B., Ouyang, Z., Cotleur, A.C., Peterson, M., Allaire, N., Challa, R., Cullen, P., et al. (2019). Cx3cr1-deficient microglia exhibit a premature aging transcriptome. Life Sci. Alliance *2*, e201900453. https://doi.org/10.26508/lsa.201900453.

38. Foo, L.C. (2013). Purification of rat and mouse astrocytes by immunopanning. Cold Spring Harbor Protoc. *2013*, 421–432. https://doi.org/10.1101/pdb.prot074211.

39. Scholze, A.R., Foo, L.C., Mulinyawe, S., and Barres, B.A. (2014). BMP signaling in astrocytes downregulates EGFR to modulate survival and maturation. PLoS One *9*, e110668. https://doi.org/10.1371/journal.pone.0110668.

40. Bayraktar, O.A., Bartels, T., Holmqvist, S., Kleshchevnikov, V., Martirosyan, A., Polioudakis, D., Ben Haim, L., Young, A.M.H., Batiuk, M.Y., Prakash, K., et al. (2020). Astrocyte layers in the mammalian cerebral cortex revealed by a single-cell in situ transcriptomic map. Nat. Neurosci. *23*, 500–509. https://doi.org/10.1038/s41593-020-0602-1.

41. Batiuk, M.Y., Martirosyan, A., Wahis, J., De Vin, F., Marneffe, C., Kusserow, C., Koeppen, J., Viana, J.F., Oliveira, J.F., Voet, T., et al. (2020). Identification of region-specific astrocyte subtypes at single cell resolution. Nat. Commun. *11*, 1220. https://doi.org/10.1038/s41467-019-14198-8.

42. Weinreb, C., Wolock, S., and Klein, A.M. (2018). Spring: a kinetic interface for visualizing high dimensional single-cell expression data. Bioinformatics *34*, 1246–1248.

43. Llorens-Bobadilla, E., Zhao, S., Baser, A., Saiz-Castro, G., Zwadlo, K., and Martin-Villalba, A. (2015). Single-cell transcriptomics reveals a population of dormant neural stem cells that become activated upon brain

injury. Cell Stem Cell *17*, 329–340. https://doi.org/10.1016/j.stem.2015.07.002.

44. Shin, J., Berg, A., Daniel, Z.Y., Shin, Y., Joseph, S.J., Bonaguidi, A., Michael, E.G., Nauen, W., David, C.M., Kimberly, M.G.-L., and Song, H. (2015). Single-Cell RNA-Seq with Waterfall Reveals Molecular Cascades underlying Adult Neurogenesis. Cell Stem Cell *17*, 360–372. https://doi.org/10.1016/j.stem.2015.07.013.

45. Laywell, E.D., Rakic, P., Kukekov, V.G., Holland, E.C., and Steindler, D.A. (2000). Identification of a multipotent astrocytic stem cell in the immature and adult mouse brain. Proc. Natl. Acad. Sci. USA *97*, 13883–13888. https://doi.org/10.1073/pnas.250471697.

46. Korsunsky, I., Millard, N., Fan, J., Slowikowski, K., Zhang, F., Wei, K., Baglaenko, Y., Brenner, M., Loh, P.R., and Raychaudhuri, S. (2019). Fast, sensitive and accurate integration of single-cell data with Harmony. Nat. Methods *16*, 1289–1296. https://doi.org/10.1038/s41592-019-0619-0.

47. Baser, A., Skabkin, M., Kleber, S., Dang, Y., Gülcüler Balta, G.S., Kalamakis, G., Göpferich, M., Ibañez, D.C., Schefzik, R., Lopez, A.S., et al. (2019). Onset of differentiation is post-transcriptionally controlled in adult neural stem cells. Nature *566*, 100–104. https://doi.org/10.1038/s41586-019-0888-x.

48. Romano, R., and Bucci, C. (2020). Role of EGFR in the nervous system. Cells *9*, 1887. https://doi.org/10.3390/cells9081887.

49. Kawase, T., Ohki, R., Shibata, T., Tsutsumi, S., Kamimura, N., Inazawa, J., Ohta, T., Ichikawa, H., Aburatani, H., Tashiro, F., and Taya, Y. (2009). PH domain-only protein PHLDA3 is a p53-regulated repressor of Akt. Cell *136*, 535–550. https://doi.org/10.1016/j.cell.2008.12.002.

50. Budanov, A.V., and Karin, M. (2008). p53 target genes Sestrin1 and Sestrin2 connect genotoxic stress and mTOR signaling. Cell *134*, 451–460. https://doi.org/10.1016/j.cell.2008.06.028.

51. Thoreen, C.C., Chantranupong, L., Keys, H.R., Wang, T., Gray, N.S., and Sabatini, D.M. (2012). A unifying model for mTORC1-mediated regulation of mRNA translation. Nature *485*, 109–113. https://doi.org/10.1038/nature11083.

52. Magnusson, J.P., and Frisén, J. (2016). Stars from the darkest night: unlocking the neurogenic potential of astrocytes in different brain regions. Development *143*, 1075–1086. https://doi.org/10.1242/dev.133975.

53. Amit, M., Takahashi, H., Dragomir, M.P., Lindemann, A., Gleber-Netto, F.O., Pickering, C.R., Anfossi, S., Osman, A.A., Cai, Y., Wang, R., et al. (2020). Loss of p53 drives neuron reprogramming in head and neck cancer. Nature *578*, 449–454. https://doi.org/10.1038/s41586-020-1996-3.

54. Boutelle, A.M., and Attardi, L.D. (2021). p53 and tumor suppression: it takes a network. Trends Cell Biol. *31*, 298–310. https://doi.org/10.1016/j.tcb.2020.12.011.

55. Kim, N.H., Kim, H.S., Li, X.-Y., Lee, I., Choi, H.-S., Kang, S.E., Cha, S.Y., Ryu, J.K., Yoon, D., Fearon, E.R., et al. (2011). A p53/miRNA-34 axis regulates Snail1-dependent cancer cell epithelial–mesenchymal transition. J. Cell Biol. *195*, 417–433. https://doi.org/10.1083/jcb.201103097.

56. Choi, Y.J., Lin, C.-P., Ho, J.J., He, X., Okada, N., Bu, P., Zhong, Y., Kim, S.Y., Bennett, M.J., Chen, C., et al. (2011). miR-34 miRNAs provide a barrier for somatic cell reprogramming. Nat. Cell Biol. *13*, 1353–1360. https://doi.org/10.1038/ncb2366.

57. Marjanovic, N.D., Hofree, M., Chan, J.E., Canner, D., Wu, K., Trakala, M., Hartmann, G.G., Smith, O.C., Kim, J.Y., Evans, K.V., et al. (2020). Emergence of a high-plasticity cell state during lung cancer evolution. Cancer Cell *38*, 229–246.e13. https://doi.org/10.1016/j.ccell.2020.06.012.

58. Martincorena, I., and Campbell, P.J. (2015). Somatic mutation in cancer and normal cells. Science *349*, 1483–1489. https://doi.org/10.1126/science.aab4082.

59. Martincorena, I., Fowler, J.C., Wabik, A., Lawson, A.R.J., Abascal, F., Hall, M.W.J., Cagan, A., Murai, K., Mahbubani, K., Stratton, M.R., et al. (2018). Somatic mutant clones colonize the human esophagus with age. Science *362*, 911–917. https://doi.org/10.1126/science.aau3879.

60. Yizhak, K., Aguet, F., Kim, J., Hess, J.M., Kübler, K., Grimsby, J., Frazer, R., Zhang, H., Haradhvala, N.J., Rosebrock, D., et al. (2019). RNA sequence analysis reveals macroscopic somatic clonal expansion across normal tissues. Science *364*, eaaw0726. https://doi.org/10.1126/science.aaw0726.

61. Moore, L., Leongamornlert, D., Coorens, T.H.H., Sanders, M.A., Ellis, P., Dentro, S.C., Dawson, K.J., Butler, T., Rahbari, R., Mitchell, T.J., et al. (2020). The mutational landscape of normal human endometrial epithelium. Nature *580*, 640–646. https://doi.org/10.1038/s41586-020-2214-z.

62. Lee-Six, H., Olafsson, S., Ellis, P., Osborne, R.J., Sanders, M.A., Moore, L., Georgakopoulos, N., Torrente, F., Noorani, A., Goddard, M., et al. (2019). The landscape of somatic mutation in normal colorectal epithelial cells. Nature *574*, 532–537. https://doi.org/10.1038/s41586-019-1672-7.

63. Codega, P., Silva-Vargas, V., Paul, A., Maldonado-Soto, A.R., Deleo, A.M., Pastrana, E., and Doetsch, F. (2014). Prospective identification and purification of quiescent adult neural stem cells from their in vivo niche. Neuron *82*, 545–559. https://doi.org/10.1016/j.neuron.2014.02.039.

64. Verhaak, R.G.W., Hoadley, K.A., Purdom, E., Wang, V., Qi, Y., Wilkerson, M.D., Miller, C.R., Ding, L., Golub, T., Mesirov, J.P., et al. (2010). Integrated genomic analysis identifies clinically relevant subtypes of glioblastoma characterized by abnormalities in PDGFRA, IDH1, EGFR, and NF1. Cancer Cell *17*, 98–110. https://doi.org/10.1016/j.ccr.2009.12.020.

65. Alcantara Llaguno, S., Sun, D., Pedraza, A.M., Vera, E., Wang, Z., Burns, D.K., and Parada, L.F. (2019). Cell-of-origin susceptibility to glioblastoma formation declines with neural lineage restriction. Nat. Neurosci. *22*, 545–555. https://doi.org/10.1038/s41593-018-0333-8.

66. Azzarelli, R., Simons, B.D., and Philpott, A. (2018). The developmental origin of brain tumors: a cellular and molecular framework. Development *145*, dev162693. https://doi.org/10.1242/dev.162693.

67. Leonard, J.R., D'Sa, C., Klocke, B.J., and Roth, K.A. (2001). Neural precursor cell apoptosis and glial tumorigenesis following transplacental ethyl-nitrosourea exposure. Oncogene *20*, 8281–8286. https://doi.org/10.1038/sj.onc.1205024.

68. Akgül, S., Li, Y., Zheng, S., Kool, M., Treisman, D.M., Li, C., Wang, Y., Gröbner, S., Ikenoue, T., Shen, Y., et al. (2018). Opposing tumor-promoting and -Suppressive functions of Rictor/mTORC2 signaling in adult glioma and pediatric SHH medulloblastoma. Cell Rep. *24*, 463–478.e5. https://doi.org/10.1016/j.celrep.2018.06.050.

69. Li, Y., Li, B., Li, W., Wang, Y., Akgül, S., Treisman, D.M., Heist, K.A., Pierce, B.R., Hoff, B., Ho, C.Y., et al. (2020). Murine models of IDH-wild-type glioblastoma exhibit spatial segregation of tumor initiation and manifestation during evolution. Nat. Commun. *11*, 3669. https://doi.org/10.1038/s41467-020-17382-3.

70. Wang, Y., Yang, J., Zheng, H., Tomasek, G.J., Zhang, P., McKeever, P.E., Lee, E.Y., and Zhu, Y. (2009). Expression of mutant p53 proteins implicates a lineage relationship between neural stem cells and malignant astrocytic glioma in a murine model. Cancer Cell *15*, 514–526. https://doi.org/10.1016/j.ccr.2009.04.001.

71. Boscolo Sesillo, F., Fox, D., and Sacco, A. (2019). Muscle stem cells give rise to rhabdomyosarcomas in a severe mouse model of Duchenne muscular dystrophy. Cell Rep. *26*, 689–701.e6. https://doi.org/10.1016/j.celrep.2018.12.089.

72. Tyagi, V., Theobald, J., Barger, J., Bustoros, M., Bayin, N.S., Modrek, A.S., Kader, M., Anderer, E.G., Donahue, B., Fatterpekar, G., and Placantonakis, D.G. (2016). Traumatic brain injury and subsequent glioblastoma development: review of the literature and case reports. Surg. Neurol. Int. *7*, 78. https://doi.org/10.4103/2152-7806.189296.

73. Anselmi, E., Vallisa, D., Bertè, R., Vanzo, C., and Cavanna, L. (2006). Post-traumatic glioma: report of two cases. Tumori *92*, 175–177.

74. Zhou, B., and Liu, W. (2010). Post-traumatic glioma: report of one case and review of the literature. Int. J. Med. Sci. *7*, 248–250. https://doi.org/10.7150/ijms.7.248.

75. Coskun, S., Coskun, A., Gursan, N., and Aydin, M.D. (2011). Post-traumatic glioblastoma multiforme: a case report. Eurasian J. Med. *43*, 50–53. https://doi.org/10.5152/eajm.2011.10.

76. Juškys, R., and Chomanskis, Ž. (2020). Glioblastoma following traumatic brain injury: case report and literature review. Cureus *12*, e8019. https://doi.org/10.7759/cureus.8019.

77. Stensjøen, A.L., Solheim, O., Kvistad, K.A., Håberg, A.K., Salvesen, Ø., and Berntsen, E.M. (2015). Growth dynamics of untreated glioblastomas in vivo. Neuro. Oncol *17*, 1402–1411. https://doi.org/10.1093/neuonc/nov029.

78. Burger, P.C., Dubois, P.J., Schold, S.C., Smith, K.R., Odom, G.L., Crafts, D.C., and Giangaspero, F. (1983). Computerized tomographic and pathologic studies of the untreated, quiescent, and recurrent glioblastoma multiforme. J. Neurosurg. *58*, 159–169. https://doi.org/10.3171/jns.1983.58.2.0159.

79. De Bonis, P., Anile, C., Pompucci, A., Fiorentino, A., Balducci, M., Chiesa, S., Lauriola, L., Maira, G., and Mangiola, A. (2013). The influence of surgery on recurrence pattern of glioblastoma. Clin. Neurol. Neurosurg. *115*, 37–43. https://doi.org/10.1016/j.clineuro.2012.04.005.

80. Okolie, O., Bago, J.R., Schmid, R.S., Irvin, D.M., Bash, R.E., Miller, C.R., and Hingtgen, S.D. (2016). Reactive astrocytes potentiate tumor aggressiveness in a murine glioma resection and recurrence model. Neuro. Oncol *18*, 1622–1633. https://doi.org/10.1093/neuonc/now117.

81. Turnquist, C., Harris, B.T., and Harris, C.C. (2020). Radiation-induced brain injury: current concepts and therapeutic strategies targeting neuroinflammation. Neurooncol. Adv. *2*, vdaa057. https://doi.org/10.1093/noajnl/vdaa057.

82. Niraula, A., Sheridan, J.F., and Godbout, J.P. (2017). Microglia priming with aging and stress. Neuropsychopharmacology *42*, 318–333. https://doi.org/10.1038/npp.2016.185.

83. Lourbopoulos, A., Ertürk, A., and Hellal, F. (2015). Microglia in action: how aging and injury can change the brain's guardians. Front. Cell. Neurosci. *9*, 54. https://doi.org/10.3389/fncel.2015.00054.

84. Gan, S., Shi, W., Wang, S., Sun, Y., Yin, B., Bai, G., Jia, X., Sun, C., Niu, X., Wang, Z., et al. (2021). Accelerated brain aging in mild traumatic brain injury: longitudinal pattern recognition with white matter integrity. J. Neurotrauma *38*, 2549–2559. https://doi.org/10.1089/neu.2020.7551.

85. Godar, S., Ince, T.A., Bell, G.W., Feldser, D., Donaher, J.L., Bergh, J., Liu, A., Miu, K., Watnick, R.S., Reinhardt, F., et al. (2008). Growth-inhibitory and tumor- suppressive functions of p53 depend on its repression of CD44 expression. Cell *134*, 62–73. https://doi.org/10.1016/j.cell.2008.06.006.

86. Moffat, J., Grueneberg, D.A., Yang, X., Kim, S.Y., Kloepfer, A.M., Hinkle, G., Piqani, B., Eisenhaure, T.M., Luo, B., Grenier, J.K., et al. (2006). A lentiviral RNAi library for human and mouse genes applied to an arrayed viral high-content screen. Cell *124*, 1283–1298. https://doi.org/10.1016/j.cell.2006.01.040.

87. Tang, W., Bertaux, F., Thomas, P., Stefanelli, C., Saint, M., Marguerat, S., and Shahrezaei, V. (2020). bayNorm: bayesian gene expression recovery, imputation and normalization for single-cell RNA-sequencing data. Bioinformatics *36*, 1174–1181. https://doi.org/10.1093/bioinformatics/btz726.

88. Stuart, T., Butler, A., Hoffman, P., Hafemeister, C., Papalexi, E., Mauck, W.M., III, Hao, Y., Stoeckius, M., Smibert, P., and Satija, R. (2019). Comprehensive integration of single-cell data. Cell *177*, 1888–1902.e21. e1821.

89. Ewels, P.A., Peltzer, A., Fillinger, S., Patel, H., Alneberg, J., Wilm, A., Garcia, M.U., Di Tommaso, P., and Nahnsen, S. (2020). The nf-core framework for community-curated bioinformatics pipelines. Nat. Biotechnol. *38*, 276–278. https://doi.org/10.1038/s41587-020-0439-x.

90. McQuin, C., Goodman, A., Chernyshev, V., Kamentsky, L., Cimini, B.A., Karhohs, K.W., Doan, M., Ding, L., Rafelski, S.M., Thirstrup, D., et al. (2018). CellProfiler 3.0: next-generation image processing for biology. PLoS Biol. *16*, e2005970. https://doi.org/10.1371/journal.pbio.2005970.

91. Schildge, S., Bohrer, C., Beck, K., and Schachtrup, C. (2013). Isolation and culture of mouse cortical astrocytes. J. Vis. Exp. https://doi.org/10.3791/50079.

92. Sun, X., Hu, X., Wang, D., Yuan, Y., Qin, S., Tan, Z., Gu, Y., Huang, X., He, C., and Su, Z. (2017). Establishment and characterization of primary astrocyte culture from adult mouse brain. Brain Res. Bull. *132*, 10–19. https://doi.org/10.1016/j.brainresbull.2017.05.002.

93. Pollard, S.M., Yoshikawa, K., Clarke, I.D., Danovi, D., Stricker, S., Russell, R., Bayani, J., Head, R., Lee, M., Bernstein, M., et al. (2009). Glioma stem cell lines expanded in adherent culture have tumor-specific phenotypes and are suitable for chemical and genetic screens. Cell Stem Cell *4*, 568–580. https://doi.org/10.1016/j.stem.2009.03.014.

94. Belenguer, G., Domingo-Muelas, A., Ferrón, S.R., Morante-Redolat, J.M., and Fariñas, I. (2016). Isolation, culture and analysis of adult subependymal neural stem cells. Differentiation *91*, 28–41. https://doi.org/10.1016/j.diff.2016.01.005.

95. Schindelin, J., Arganda-Carreras, I., Frise, E., Kaynig, V., Longair, M., Pietzsch, T., Preibisch, S., Rueden, C., Saalfeld, S., Schmid, B., et al. (2012). Fiji: an open-source platform for biological-image analysis. Nat. Methods *9*, 676–682. https://doi.org/10.1038/nmeth.2019.

96. Hagemann-Jensen, M., Ziegenhain, C., Chen, P., Ramsköld, D., Hendriks, G.J., Larsson, A.J.M., Faridani, O.R., and Sandberg, R. (2020). Single-cell RNA counting at allele and isoform resolution using Smart-seq3. Nat. Biotechnol. *38*, 708–714. https://doi.org/10.1038/s41587-020-0497-0.

97. Grün, D. (2020). Revealing dynamics of gene expression variability in cell state space. Nat. Methods *17*, 45–49.

98. Finak, G., McDavid, A., Yajima, M., Deng, J., Gersuk, V., Shalek, A.K., Slichter, C.K., Miller, H.W., McElrath, M.J., and Prlic, M. (2015). MAST: a flexible statistical framework for assessing transcriptional changes and characterizing heterogeneity in single-cell RNA sequencing data. Genome Biol. *16*, 278.

99. Subramanian, A., Tamayo, P., Mootha, V.K., Mukherjee, S., Ebert, B.L., Gillette, M.A., Paulovich, A., Pomeroy, S.L., Golub, T.R., Lander, E.S., et al. (2005). Gene set enrichment analysis: a knowledge-based approach for interpreting genome-wide expression profiles. Proc. Natl. Acad. Sci. USA *102*, 15545–15550. https://doi.org/10.1073/pnas.0506580102.

100. Mootha, V.K., Lindgren, C.M., Eriksson, K.F., Subramanian, A., Sihag, S., Lehar, J., Puigserver, P., Carlsson, E., Ridderstråle, M., Laurila, E., et al. (2003). PGC-1alpha-responsive genes involved in oxidative phosphorylation are coordinately downregulated in human diabetes. Nat. Genet. *34*, 267–273. https://doi.org/10.1038/ng1180.

101. Bergen, V., Lange, M., Peidli, S., Wolf, F.A., and Theis, F.J. (2020). Generalizing RNA velocity to transient cell states through dynamical modeling. Nat. Biotechnol. *38*, 1408–1414.

102. La Manno, G., Soldatov, R., Zeisel, A., Braun, E., Hochgerner, H., Petukhov, V., Lidschreiber, K., Kastriti, M.E., Lönnerberg, P., Furlan, A., et al. (2018). RNA velocity of single cells. Nature *560*, 494–498. https://doi.org/10.1038/s41586-018-0414-6.

103. Cao, J., Spielmann, M., Qiu, X., Huang, X., Ibrahim, D.M., Hill, A.J., Zhang, F., Mundlos, S., Christiansen, L., and Steemers, F.J. (2019). The single-cell transcriptional landscape of mammalian organogenesis. Nature *566*, 496–502.

104. Angerer, P., Haghverdi, L., Büttner, M., Theis, F.J., Marr, C., and Buettner, F. (2016). destiny: diffusion maps for large-scale single-cell data in R. Bioinformatics *32*, 1241–1243. https://doi.org/10.1093/bioinformatics/btv715.

105. Hafemeister, C., and Satija, R. (2019). Normalization and variance stabilization of single-cell RNA-seq data using regularized negative binomial regression. Genome Biol. *20*, 296. https://doi.org/10.1186/s13059-019-1874-1.

106. Zhu, L.J., Gazin, C., Lawson, N.D., Pagès, H., Lin, S.M., Lapointe, D.S., and Green, M.R. (2010). ChIPpeakAnno: a Bioconductor package to

annotate ChIP-seq and ChIP-chip data. BMC Bioinformatics *11*, 237. https://doi.org/10.1186/1471-2105-11-237.

107. Andreassi, C., Luisier, R., Crerar, H., Darsinou, M., Blokzijl-Franke, S., Lenn, T., Luscombe, N.M., Cuda, G., Gaspari, M., Saiardi, A., and Riccio, A. (2021). Cytoplasmic cleavage of IMPA1 3′ UTR is necessary for maintaining axon integrity. Cell Rep. *34*, 108778. https://doi.org/10.1016/j.celrep.2021.108778.

108. Johannes, G., and Sarnow, P. (1998). Cap-independent polysomal association of natural mRNAs encoding c-myc, BiP, and eIF4G conferred by internal ribosome entry sites. RNA *4*, 1500–1513. https://doi.org/10.1017/s1355838298981080.

109. Picelli, S., Faridani, O.R., Björklund, A.K., Winberg, G., Sagasser, S., and Sandberg, R. (2014). Full-length RNA-seq from single cells using Smart-seq2. Nat. Protoc. *9*, 171–181. https://doi.org/10.1038/nprot.2014.006.

110. Singh, B., Carpenter, G., and Coffey, R.J. (2016). EGF receptor ligands: recent advances. F1000Res *5*, 2270. https://doi.org/10.12688/f1000research.9025.1.

CellPress
OPEN ACCESS

STAR★METHODS

KEY RESOURCES TABLE

REAGENT or RESOURCE	SOURCE	IDENTIFIER
**Antibodies**		
goat polyclonal anti-Apolipoprotein E (ApoE)	Millipore	Cat#AB947; RRID: AB_2258475
rabbit polyclonal anti-Ascl1	Cosmo Bio	Cat#CAC-SK-T01-003; RRID: AB_10709354
rabbit anti-β-actin (D6A8)	Cell Signalling Technology	Cat#8547; RRID: AB_10950489
rabbit anti-cleaved caspase 3 (Asp175) (5A1E)	Cell Signalling Technology	Cat#9664; RRID: AB_2070042
rat monoclonal anti-CD68 (clone FA-11)	Abcam	Cat#ab53444; RRID: AB_869007
rabbit polyclonal anti-doublecortin (DCX)	Abcam	Cat#ab18723; RRID: AB_732011
rabbit polyclonal anti-GFAP	DAKO	Cat#Z0334; RRID: AB_10013382
mouse monoclonal anti-GFAP (clone GA5)	Millipore	Cat#MAB3402; RRID: AB_94844
rabbit polyclonal anti-Iba1	Wako	Cat#019-19741; RRID: AB_839504
rabbit monoclonal anti-Ki67 [SP6]	Abcam	Cat#ab16667; RRID: AB_302459
rabbit polyclonal anti-NFIA	Atlas antibodies	Cat#HPA006111; RRID: AB_1854422
mouse IgM anti-O4	R&D	Cat#MAB1326; RRID: AB_357617
rabbit polyclonal anti-Olig2	Millipore	Cat#AB9610; RRID: AB_570666
rabbit anti-Olig2 [EPR2673]	Abcam	Cat#ab109186; RRID: AB_10861310
rabbit polyclonal anti-p53	Leica	Cat#p53-CM5; RRID: AB_2895247
rabbit polyclonal anti-PCP4	Atlas antibodies	Cat#HPA005792; RRID: AB_1855086
goat polyclonal anti-PDGFRα	R&D	Cat#AF1062; RRID: AB_2236897
rabbit polyclonal anti-RFP	Antibodies-online	Cat#ABIN129578; RRID: AB_10781500
mouse anti-S6 Ribosomal Protein (54D2)	Cell Signalling Technology	Cat#2317; RRID: AB_2238583
rabbit anti-phospho-S6 ribosomal protein (S240/244)	Cell Signalling Technology	Cat#5364; RRID: AB_10694233
rabbit polyclonal anti-Sox2	Abcam	Cat#ab97959; RRID: AB_2341193
mouse monoclonal anti-Sox2 [9-9-3]	Abcam	Cat#ab79351; RRID: AB_10710406
rabbit anti-Tubulin3 (TUBB3) (Tuj1)	Biolegend	Cat#845501; RRID: AB_2566588
mouse anti-vimentin (clone V9)	Abcam	Cat#ab8069; RRID: AB_306239
**Bacterial and virus strains**		
Ad-CMV-Null	Vector Biolabs	Cat#1300
Ad-CMV-iCre	Vector Biolabs	Cat#1045
Ad-GFAP-Cre	University of Iowa Viral Vector Core	n/a
**Chemicals, peptides, and recombinant proteins**		
EdU	Santa Cruz	Cat#sc-284628
Tamoxifen	Sigma	Cat#T5648
Endoxifen	Tocris	Cat#3705
LPS (*E. coli* O55:B5)	Merck	Cat#L2637
Osimertinib	AKT Labs	Cat#A985134
EGF	Peprotech	Cat# 315-09-1000
Torin1	Generon	Cat# A8312
4OHT	Sigma	Cat#H7904
Trypsin	Sigma	Cat#T4799
Fetal bovine serum (FBS)	Sigma	Cat#F7524
Poly-L-lysine hydrobromide (PLL)	Sigma	Cat#P1274
BMP4	Peprotech	Cat#315-27
FGF	Peprotech	Cat#450-33A

(*Continued on next page*)

Continued		
REAGENT or RESOURCE	SOURCE	IDENTIFIER
AstroMACS Medium	Miltenyi	Cat#130-117-031
Low melting point agarose	Invitrogen	Cat#16520-100
AMPure XP beads	Beckman Coulter	Cat#A63881
Critical commercial assays		
Adult Brain Dissociation Kit	Miltenyi	Cat#130-107-677
Click-iT™ EdU Alexa Fluor™ 647 Imaging kit	Invitrogen	Cat#C10340
Click-iT™ Plus OPP Alexa Fluor™ 488 kit	Invitrogen	Cat#C10456
iScript gDNA clear cDNA synthesis kit	Bio-rad	Cat#1725034
10x Genomics Single Cell 3’ v2 kit	10X Genomics	Cat#PN-120237
Chromium Next GEM Single Cell 3’ Kit v3.1	10X Genomics	Cat#1000269
EZ-Magna ChIP kit	Millipore	Cat#MAGNA0002
NEBNext Ultra II DNA library prep kit	NEB	Cat# E7645S
Deposited data		
Postnatal scRNA-seq data	This paper	GEO: GSE196408
Adult scRNA-seq data	This paper	GEO: GSE220477
In vivo scRNA-seq data	This paper	GEO: GSE220479
ChIP-seq data	This paper	GEO: GSE196863
Zamboni et al scRNA-seq dataset	Zamboni et al.[1]	GEO: GSE139842
Li et al RNA-seq dataset	Li et al.[20]	GEO: GSE147870
Guttenplan et al RNA-seq dataset	Guttenplan et al.[36]	GEO: GSE143598
Gyoneva et al RNA-seq dataset	Gyoneva et al.[37]	GEO: GSE131869
Experimental models: Cell lines		
Primary Human astrocytes	Sciencell	Cat#1800
Experimental models: Organisms/strains		
Mouse: GFAP-CreER: Tg(GFAP-cre/ERT2)1Fki	Hirrlinger et al.[23]	MGI:4418665
Mouse: Glast-CreERT2: Slc1a3$^{tm1(cre/ERT2)Mgoe}$	Mori et al.[29]	MGI:3830051
Mouse: *p53*$^{flox/flox}$: Trp53^{tm1Brn}	Marino et al.[24]	MGI:1931011
Mouse: LSL-tdTomato: B6.Cg-Gt(ROSA) 26Sor$^{tm14(CAG-tdTomato)Hze}$/J	The Jackson Laboratory; Madisen et al.[25]	Cat#007914; RRID:IMSR_JAX:007914
Oligonucleotides		
See Table S1 for primer sequences		
Recombinant DNA		
Plasmid: shp53 pLKO.1	Godar et al.[85]	Addgene plasmid # 19119; RRID:Addgene_19119
Plasmid: pLKO.1 - TRC control	Moffat et al.[86]	Addgene plasmid # 10879; RRID:Addgene_10879
Software and algorithms		
CellRanger v3.1.0	10X Genomics	https://support.10xgenomics.com/single-cell-gene-expression/software
bcl2fastq v2.20.0	Illumina	https://emea.support.illumina.com/sequencing/sequencing_software/bcl2fastq-conversion-software.html
R v4.0.4	CRAN	https://www.r-project.org/
bayNorm v1.8.0	Tang et al.[87]	https://bioconductor.org/packages/bayNorm/
Seurat v4.0.4	Stuart et al.[88]	https://cloud.r-project.org/web/packages/Seurat/index.html
SPRING	Weinreb et al.[42]	https://kleintools.hms.harvard.edu/tools/spring.html

(*Continued on next page*)

Continued

REAGENT or RESOURCE	SOURCE	IDENTIFIER
Nextflow nf-core/chipseq pipeline v1.0dev	Ewels et al.[89]	https://github.com/nf-core/chipseq; https://doi.org/10.5281/zenodo.3240506
Fiji Image J	Schindelin et al.[88]	https://imagej.nih.gov/ij/
Cellprofiler	McQuin et al.[90]	http://cellprofiler.org RRID:SCR_007358
Other		
Alzet Brain Infusion kit 3	Alzet	Cat#0008851
Alzet Osmotic minipump (model 1002)	Alzet	Cat#1002

RESOURCE AVAILABILITY

Lead contact

Further information and requests for resources and reagents should be directed to and will be fulfilled by the lead contact, Simona Parrinello (s.parrinello@ucl.ac.uk).

Materials availability

This study did not generate new unique reagents.

Data and code availability

- The scRNA-seq and ChIP-seq data have been deposited at GEO and are publicly available as of the date of publication. Accession numbers are listed in the key resources table.
- This paper does not report original code.
- Any additional information required to reanalyse the data reported in this paper is available from the lead contact upon request.

EXPERIMENTAL MODEL AND SUBJECT DETAILS

Mice

All animal procedures were carried out in accordance with the Animal Scientific Procedures Act, 1986 and approved by the UCL Animal Welfare and Ethical Review Body (AWERB) in accordance with local ethical and care guidelines and the International guidelines of the Home Office (UK). In order to fate map and induce gene deletion in astrocytes, GFAP-CreER (Tg(GFAP-cre/ERT2)1Fki)[23] or Glast-CreERT2 (Slc1a3$^{tm1(cre/ERT2)Mgoe}$)[29] were crossed to mice with conditional p53 deletion (*p53*$^{flox/flox}$) (Trp53^{tm1Brn})[24] or *p53*$^{+/+}$ (*p53*$^{wt/wt}$). A CAG-LoxP-STOP-LoxP tdTomato reporter in the Rosa26 locus (LSL-tdTomato) (B6.Cg-Gt(ROSA)26Sor$^{tm14(CAG-tdTomato)Hze}$/J)[25] was included in all crosses to trace recombined cells. All animals were in a mixed 129Sv x C57BL/6 background and both males and females were analysed. Approximately an equal number of male and female mice were used per experiment. Mice were group-housed (where possible) in individually ventilated cages and maintained with 12-hour light/dark cycles with water and chow available *ad libitum*.

Primary postnatal astrocyte isolation and culture

For isolation of primary cortical astrocytes, litters of mice of indicated genotypes aged postnatal day (P3) were sacrificed by decapitation. For experiments with GFAP-CreER astrocytes, brains were processed separately and combined at first passage after genotyping for GFAP-CreER allele. For other experiments, brains were pooled prior to digestion. Digestion protocol was based on.[91] Briefly, the cortices were dissected in HEPES-buffered HBSS, meninges removed, and cut into small pieces before digestion in 0.25 % trypsin (Sigma, T4799) in HBSS for 30 min at 37°C followed by centrifugation (5 min, 300xg). Tissue pieces were dissociated by trituration in astrocyte expansion media (DMEM-Glutamax, 10% FBS (Sigma, F7524), 100 μg/ml kanamycin, 2 μg/ml gentamicin) and plated onto poly-L-lysine hydrobromide (PLL, 1:2500, Sigma P1274) coated plates. Cells were cultured at 37 °C, 5 % CO_2. Debris was removed the following day by washing in PBS and after 6-7 days in culture microglia and macrophages, oligodendrocytes and OPCs and neurons were removed by immunopanning using CD45, O4 and L1 antibodies as described in [38]. Recovered astrocytes were plated in astrocyte expansion media. Once confluency was reached astrocytes were passaged and plated at a density of $20x10^3$ cells/cm^2 and cultured until induction of recombination in astrocyte maturation media (50% Neurobasal, 50% DMEM, 1mM sodium pyruvate, 292μg/ml L-glutamine, 1X SATO, 5μg/ml M-acetylcysteine, 100μg/ml kanamycin, 2μg/ml gentamicin,[38] supplemented with 25ng/ml BMP4 (Peprotech)).

Primary adult astrocyte isolation and culture

For isolation of primary cortical astrocytes from adult mice, cortices from two 8–12 week old GFAP-CreERT2;*p53*$^{flox/flox}$;LSL-tdTomato mice were pooled and dissociated using the Adult Brain Dissociation Kit (Miltenyi 130-107-677), according to

manufacturer's instructions. Astrocytes were purified from cell suspension after removal of debris and red blood cells with ACSA-2 MicroBeads (Miltenyi 130-097-678) according to manufacturer's instructions and plated onto two wells of a PLL and laminin coated 12 well plate in AstroMACS Medium (# 130-117-031). The following day, plates were gently washed with media to remove debris. Media was replaced every 3 days thereafter. While in culture, adult astrocytes showed very limited divisions, as previously described.[92]

Human astrocyte culture

Primary human astrocytes (Sciencell #1800) were subcultured according to manufacturer's instructions. Alternatively, astrocytes were prepared by differentiation of primary human fetal neural stem cells (BRC2351, BRC2389 and BRC2404) by incubation in "basal media" (DMEM-F12, N2 (1/200), B27 (1/100), 5mM HEPES, 1x MEM NEAA. 50μM betamercaptoethanol, 0.012% BSA, 6.525mg/ml glucose, 100μg/ml kanamycin, 2μg/ml gentamicin)[93] supplemented with 10% FBS for 14 days.

METHOD DETAILS

In vivo administrations and surgeries

For localised intracortical astrocyte recombination, 2-3-month-old mice underwent stereotaxic injection of 1.807μl of endoxifen (13.3μM Tocris 3705/10) (anteroposterior -1.2, mediolateral 1.7, dorsoventral -1 relative to bregma) under isofluorane anaesthesia. To induce recombination in astrocytes in an injury-free setting, 2-3-month-old mice were injected once daily for 5 days intraperitoneally (i.p.) with 100mg/kg tamoxifen (Sigma, T5648 prepared in 10% ethanol, 90% corn oil). For lipopolysaccharide (LPS) experiments, mice were injected with LPS (*E. coli* O55:B5, Merck L2637, dissolved in PBS) 1mg/kg i.p. 11 days after endoxifen injection and sacrificed 3 days later. To investigate the response of injured cortical astrocytes in the absence of EGFR signalling the EGFR inhibitor osimertinib was used. 2-4-month-old mice were injected i.p. with 15 mg/kg osimertinib (A985134, AKT Labs, in 10% DMSO/90% corn oil) or vehicle control. Injections were started 2 days prior to intracortical endoxifen injection (as above) and on alternate days for the following 2 weeks when animals were sacrificed. For EGF infusion experiments, a brain infusion cannula (Alzet Brain Infusion kit 3 adjusted for cortical targeting using 4 spacers) attached to an overnight pre-equilibrated mini-osmotic pump (Alzet, 1002) filled with EGF (Peprotech 60μg/ml in 0.9% saline;1% BSA) or vehicle control was inserted into the cortex following endoxifen injection. The mini-osmotic pump was inserted into a subcutaneous pocket according to the manufacturer's instructions.

p53 recombination in primary astrocytes

For adenoviral experiments, allele recombination was induced by overnight adenoviral infection with Ad-CMV-Null, Ad-CMV-iCre or Ad-GFAP-Cre) at multiplicity of infection of 30. Following infection, astrocytes were cultured in "basal media" (DMEM-F12, N2 (1/200), B27 (1/100), 5mM HEPES, 1x MEM NEAA. 50μM betamercaptoethanol, 0.012% BSA, 6.525mg/ml glucose, 100μg/ml kanamycin, 2μg/ml gentamicin)[93] supplemented with 20ng/mL EGF and 10ng/mL FGF-2 (Peprotech) or 25ng/ml BMP4. For 4-hydroxytamoxifen (4-OHT) experiments, after 48h in astrocyte maturation media, media was changed to "basal media" supplemented with EGF/FGF or BMP4 as above with 4OHT (200nM, Sigma H7904) or vehicle control (ethanol). For experiments involving mTOR inhibitor, Torin, cells were incubated with Torin1 (250nM in DMSO, Generon A8312,) or DMSO vehicle control.

For primary adult astrocytes, after 7 days in culture, media was changed to astrocyte maturation media, as described above, for 2 days. Recombination was induced, as described above, by changing media to "basal media" supplemented with EGF and FGF and 4OHT (Sigma H7904, 200nM). Astrocytes were incubated for 5 days before collection for scRNA-seq as described below.

For human astrocytes, p53 downregulation was induced by lentiviral infection with shp53 or vector control. shp53 pLKO.1 puro was a gift from Bob Weinberg (Addgene plasmid # 19119; http://n2t.net/addgene:19119; RRID:Addgene_19119).[85] pLKO.1 - TRC control was a gift from David Root (Addgene plasmid # 10879; http://n2t.net/addgene:10879; RRID:Addgene_10879).[86] Lentivirus was produced by co-transfection of above plasmids with VSVG and Delta8.9 into HEK293T cells using polyethylenimine, followed by viral concentration by centrifugation (3 hrs at 50,000xg at 4°C). After lentiviral transduction, astrocytes were incubated for 7 days in "basal media" supplemented with EGF and FGF or BMP4 as above, before subjecting to clonogenic or neurosphere assays or re-differentiation experiments.

Clonogenic assays

For clonogenic assays, cells were fixed (4% PFA for 15 minutes) and stained with crystal violet (0.2% in 10% ethanol) 7 days after recombination. Excess crystal violet was washed off and plates were fully dried, before scanning on a standard scanner at 1000 dpi. The number of colonies were scored manually in ImageJ.

Neurosphere assay and re-differentiation

After 7 days in basal media containing EGF and FGF, cells were passaged and plated for neurosphere assays. To assess self-renewal, 10,000 cells were plated in 7ml neurosphere media (DMEM-F12, 1x B27 without Vitamin A, 4μg/ml heparin, 10ng/ml FGF, 20ng/ml EGF, 100μg/ml kanamycin, 2μg/ml gentamicin) in uncoated T25 flasks. After 7 days, neurospheres were scored, cells passaged as described in Belenguer et al.,[94] and replated as described above.

For assessment of multi-lineage differentiation, 50,000 cells were seeded onto PLL and laminin-coated glass coverslips in "basal media" supplemented with EGF and FGF. The following day, media was replaced with basal media supplemented with FGF only.

After 48 hours, media was replaced with “basal media” in the absence of additional growth factors and cells were incubated for a further 48 hours before fixation.

Astrocyte soft agar experiments

Primary *p53*$^{flox/flox}$ astrocytes were recombined with adenovirus (Ad-Null or Ad-GFAP-Cre) and incubated in media supplemented with EGF+FGF or BMP4 as described above. Upon reaching confluency, *p53*$^{-/-}$ EGF/FGF cells were passaged with accutase and replated onto laminin-coated dishes. Cells from other conditions were non-dividing, so did not reach confluency, but were periodically passaged to smaller wells. Anchorage-independence (a read out of tumorigenicity) was monitored roughly every 3 passages by soft agar assay. 3333 cells were plated per well in the top layer of 0.33% low melt agarose (Invitrogen) over bottom layer of 0.5% low melt agarose in a 6 well plate. Dedifferentiation basal media supplemented with EGF+FGF was overlayed to prevent drying and half media was changed every 3-4 days. After 12 days, plates were fixed and stained with crystal violet as described above. Soft agar colonies were observed after passage 9 in the *p53*$^{-/-}$ EGF/FGF condition, and not in the others.

Immunofluorescence and Immunohistochemistry

For immunohistochemical analysis, mice were sacrificed at indicated time points by transcardial perfusion with PBS followed by 4% paraformaldehyde (PFA) under terminal anaesthesia and the brain post-fixed in 4% PFA at 4°C overnight. To assess cell proliferation, EdU (50mg/kg) was administered by i.p. injection 6 hours prior to collection. 50 μm vibratome sections or 35 μm floating cryosections were permeabilised and blocked in 1% Triton X-100, 10% donkey serum, PBS for 1.5hrs then incubated overnight at 4°C with primary antibodies diluted in 0.1% Triton X-100, 10% serum, PBS. The following day, sections were washed in 0.1 % Triton X-100, PBS then incubated with secondary antibodies and DAPI in 0.1% Triton X-100, 10% serum, PBS for 1 hour at RT. Sections were washed and mounted in ProlongGold Antifade mountant.

For *in vitro* immunofluorescence, cells were incubated for 7 days as described, and fixed for 15 min in 4% PFA, permeabilised in 0.5% Triton X-100 for 20 min and blocked in 10% serum in PBS for 30 min before incubation with primary antibody overnight at 4°C in 10% serum, PBS. Coverslips were incubated with secondary antibodies and DAPI in 10% serum, PBS at RT for 1h, before mounting in ProlongGold. For experiments involving assessment of cell proliferation, cells were incubated with 10μM EdU for 2 hours prior to fixation. For experiments involving assessment of protein synthesis, cells were incubated with 20μM O-propargyl-puromycin (OP-Puro) for 30 mins prior to fixation. Click-iT EdU or OP-Puro (Invitrogen C10086 and C10458 respectively) labelling was carried out according to manufacturer’s instructions. For phalloidin staining, cells were stained with Alexa Fluor 647 phalloidin (Invitrogen, A22287) according to manufacturer’s instructions.

Primary antibodies used were goat anti-ApoE (1:1000, Millipore AB947), mouse anti-Ascl1 (1:200, gift from Francois Guillemot), rabbit anti-Ascl1 (1:500, Cosmo Bio CAC-SK-T01-003), rabbit anti-β-actin (1:200, CST 8457), rabbit anti-cleaved caspase 3 (1:500, CST 9664), rat anti-CD68 (1:500, Abcam ab53444), rabbit anti-DCX (1:1000, Abcam ab18723), rabbit anti-GFAP (1:1000, DAKO Z0334), mouse anti-GFAP (1:1000, Millipore MAB3402), rabbit anti-Iba1 (1:2000, Wako 019-19741), rabbit anti-Ki67 (1:500, Abcam ab16667), rabbit anti-NFIA (1:1000, Atlas antibodies HPA006111), mouse IgM anti-O4 (1:500 on live cells 20 min before fixation, R&D MAB1326), rabbit anti-Olig2 (1:500 *in vivo*, 1:1000 *in vitro*, Millipore AB9610), rabbit anti-Olig2 (1:500 human *in vitro*, Abcam ab109186), rabbit anti-p53 (1:500, Leica p53-CM5), rabbit anti-PCP4 (1:500, Atlas antibodies HPA005792), goat anti-PDGFRα (1:500, R&D AF1062), rabbit anti-RFP (1:400, Antibodies-online ABIN129578), mouse anti-S6 ribosomal protein (1:500, CST 2317) rabbit anti-phospho-S6 ribosomal protein (S240/244) (1:500, CST 5364), rabbit anti-Sox2 (1:400, Abcam ab97959), mouse anti-Sox2 (1:400, Abcam ab79351), rabbit anti-Tuj1 (1:500, Biolegend 845501), mouse anti-vimentin (1:500, Abcam ab8069). Alexa Fluor conjugated secondary antibodies were obtained from ThermoFisher.

Image analysis and processing

Quantifications were carried out in Fiji ImageJ.[95] For quantification of tdTomato morphology, a minimum of 200 tdTomato$^+$ cells were scored for rounded/astrocytic morphology over at least 3 sections per mouse. In injured mice, the sections chosen all had evidence of injury (dip in tissue or inflammation).

For quantification of distance of cells from wound site used in Figures 1F and 2G, tdTomato$^+$ cells were scored as astrocytic or AD-tdTomato, and the perpendicular distance was measured in ImageJ to the top of injection dip or to the injection tract, whichever was shortest. For Figures 1F, >100 cells were measured over n=3 *p53*$^{Gfap\text{-}icKO}$ animals. For Figure 2G, >200 cells were measured over n=5 *p53*$^{Gfap\text{-}icKO}$ animals.

For skeleton analysis of microglia in Figure S3C, maximum intensity projection (MIP) of confocal images of Iba1 staining were processed by despeckling, followed by binarization using the Auto Local Threshold function in ImageJ with method=Phansalkar. Images were then processed with Skeletonize followed by Analyze Skeleton. As a measure of microglia arborisation, number of junctions were normalised to the number of microglia in the field of view. For CD68 intensity analysis in Figure S3D, MIPs were despeckled and used to generate a mask of CD68 particles using the Auto Local Threshold function in ImageJ with method=Phansalkar. Analyze Particles function was then redirected to the original MIP to measure the Integrated density (IntDen) of particles >2 pixels in size. Average particle IntDen per field of view was calculated in R before normalisation to the average of uninjured young animals.

For single cell analysis of Ascl1, Sox2, Olig2 or OP-Puro intensity, a minimum of 10 fields of view were used per condition per replicate. Images were split into individual channels using ImageJ. Intensity analysis was performed using CellProfiler,[90] using a custom pipeline. Briefly, nuclei were detected and used to generate the masks. An image was generated of the masks, which was checked

for accuracy and the image/data discarded if segregation was unsuccessful. Nuclei overlapping the image border were discarded. Where applicable, tdTomato intensity was measured, and nuclei filtered for positivity. MGV was measured for the nuclear mask within each cell. Downstream analysis was performed in R: for each replicate, values were normalised to the average of the *p53*$^{+/+}$ EGF/FGF condition, before random downsampling data to lowest condition and combining replicates. Plots were generated in Graphpad Prism, whereby each point indicates a single cell.

For analysis of intensity of phospho-S6RP, images were taken of random fields of view using consistent exposure/laser settings. Images were analysed in ImageJ to generate a mask of the cytoplasm by thresholding tdTomato or pS6RP channel and DAPI to binary masks and subtracting the nuclear mask from the tdTomato mask. This mask was used to measure the pixel mean gray value (MGV) of pS6RP which was normalised to MGV of S6RP.

scRNA-sequencing of *in vitro* cells

After 5 days of incubation of GFAP-CreERT2;*p53*$^{flox/flox}$;LSL-tdTomato primary postnatal or adult astrocytes in media supplemented with EGF and FGF and 4OHT, cells were harvested by trypsinisation. Cells were washed and filtered through a 30μm MACS SmartStrainer (Miltenyi), as described in the 10X Genomics manual. Cells were counted with a haemocytometer using trypan blue for exclusion of dead cells. The volume was adjusted to cell concentration of 700-1200 cells/μl, with >80% viability. For the postnatal astrocytes, two replicates were performed from two independent astrocyte preparations, A19 and A26, processed on different days. Gel Bead-In-EMulsions (GEMs) and library preparation was performed as per the 10x Genomics Single Cell 3' v2 reagent kit protocol. The two libraries from postnatal astrocytes preps were sequenced on a single lane of Illumina HiSeq 2500 (Paired End 2 x 100bp) and de-multiplexed and analysed using CellRanger v3.1.0 and bcl2fastq v2.20.0.

For the adult *in vitro* astrocytes experiment, library preparation from single cells was performed as per the 10X Genomics Chromium Next GEM Single Cell 3' Kit v3.1 and sequenced on an Illumina Novaseq 6000 Instrument (Paired End, 2x 150bp).

Acute purification of tdTomato$^+$ cells from aged injured mice and Smartseq3 scRNA-seq

Approximately 1 year after endoxifen intracortical injection, mice were sacrificed by cervical dislocation and the area around the injection site containing tdTomato$^+$ cells was dissected under fluorescent guidance. As a negative control for tdTomato gating, the cortex of one uninjured aged mouse was used. Tissue was dissociated using the Adult brain dissociation kit (Miltenyi (130-107-677) using the protocol for <100mg tissue. To obtain sufficient injured material, we pooled mice of the same genotype (2 *p53*$^{Gfap\text{-}wt/wt}$ and 3 *p53*$^{Gfap\text{-}icKO}$). Following dissociation and debris removal, cells were sorted on a BD FACSAria III for tdTomato+ and DAPI- for live/dead discrimination. Single cells were sorted into individual wells of 384 well plates containing 3μl Smart-seq3 lysis buffer (for exact components, please refer to Hagemann-Jensen et al.[96]). Even after pooling, brains yielded small number of tdTomato+ cells from FACS (301 *p53*$^{Gfap\text{-}wt/wt}$ and 382 *p53*$^{Gfap\text{-}icKO}$), consistent with small region targeted by endoxifen injection. Plates were vortexed, spun down at 350rcf and frozen immediately at -80°C.

Prior to reverse transcription, plates were thawed on ice and incubated at 72°C for 3 min. 1μl of reverse transcription mix (for exact components please refer to Hagemann-Jensen et al.[96]) was added to each well using Echo 525 acoustic liquid handler, plate was mixed and reaction was carried out at 42°C for 90 min followed by 10 cycles of 2min at 50°C and 2min at 42°C. Reaction was terminated by incubation at 85°C for 5 mins. For pre-amplification step 6μl of PCR mix containing 1X KAPA HiFi HotStart ReadyMix, water, 0.5μM PCR forward primer and 0.1μM PCR reverse primer (for exact sequences please refer to Hagemann-Jensen et al.[96]) was added to each well using Echo 525 acoustic liquid handler, plates were vortexed and PCR performed as follows: 3 min at 98°C for initial denaturation, 24 cycles of 20 s at 98°C, 30 s at 65°C and 6 min at 72°C. Final elongation was performed for 5 min at 72°C. cDNA samples were size selected using 0.6x AMPure XP beads (A63881; Beckman Coulter), concentration was measured with Varioscan Lux (ThermoFisher) in a 384-well plate using Qubit Fluorometer solution and quality was checked on the 5200 Fragment Analyzer System (Agilent). Samples displaying satisfactory amplification traces between 300 and 9000 bp were selected for tagmentation (125 *p53*$^{Gfap\text{-}wt/wt}$ and 145 *p53*$^{Gfap\text{-}icKO}$ cells).

Sequencing libraries were made using Nextera XT DNA Library Preparation Kit (Illumina) according to manufacturer's instructions at a 20x reduced scale and using 25pg of cDNA. All reagents were distributed using Echo 525 acoustic liquid handler. After tagmentation, equal volumes of each sample were pooled and the pool was purified with 0.6x AMPure XP beads (Beckman Coulter). Concentration of the final pool was measured on Qubit 2.0 and library size distribution was checked on a high-sensitivity DNA chip (Agilent Bioanalyzer). Pool was loaded on a MiSeq Reagent Micro Kit v2 (300 cycles) at a 12pM final concentration and re-pooled based on mapping rates (123 *p53*$^{Gfap\text{-}wt/wt}$ and 141 *p53*$^{Gfap\text{-}icKO}$ cells). Final re-pooled libraries were sequenced, 150-bp paired end, on a high-output flow cell (Illumina 20024907) using an Illumina NextSeq500 instrument.

Preprocessing and normalization of scRNA-seq data

Cells and genes filtering (postnatal in vitro *astrocytes)*

We generated data for 12040 cells. High-quality cells were selected according to the following two criteria: 1) Number of detected genes ranging between 200 and 5000; 2) Proportion of mitochondria genes below 0.15 and 0.1 for batches A19 and A26 respectively; this led to 11635 high-quality cells (Figure 5B). Cells were further filtered based on tdTomato expression leading to 8699 cells with tdTomato counts above 0. In term of features, 13 mitochondrial genes were filtered out and genes with non-zero count in at least 3 cells across both batches were kept for downstream analysis. As a result, 17046 genes were included in this study. The two batches were combined before further normalisation and imputation.

Cells and genes filtering (adult in vitro *astrocytes)*

High-quality cells were selected according to the following criteria: tdTomato total counts > 0, proportion of mitochondrial genes < 0.15, and $\log_2$ total counts between 14 and 17.48. After filtering, 523 out of 1562 adult astrocytes were kept for further analysis. Genes with non-zero counts in at least 10% of all cells were kept.

Cells and genes filtering (adult in vivo *astrocytes)*

High-quality cells were selected according to the following criteria: tdTomato total counts > 0, which resulted in 99 $p53^{Gfap\text{-}wt/wt}$ and 108 $p53^{Gfap\text{-}icKO}$ cells. Genes with zero count in all cells were removed.

Normalisation and imputation (postnatal and adult in vitro *astrocytes)*

For normalisation and imputation, the bayNorm package with mean_version = TRUE was applied.[87] The bayNorm normalized counts were then imported into the Seurat package[88] for dimension reduction and clustering analysis.

Identification of cluster marker genes (postnatal in vitro *astrocytes)*

Clustering was performed in Seurat using the Louvain approach in PCA space on bayNorm normalised counts of the 2000 most variable genes. RaceID[97] was then applied to narrow down the most variable genes to 1000 genes. Marker genes in each cluster were identified using this set of 1000 genes by comparing cells in each one of the 9 clusters with all other cells using MAST.[98] Gene lists and signatures were from the GSEA website.[99,100]

Cell clustering (adult in vitro *and* in vivo *astrocytes)*

Clustering of adult astrocytes in Figure S5Kiii was performed in Seurat using the Louvain approach in PCA space on bayNorm normalised counts. Clustering of *in vivo* astrocytes in Figure S5Kiv was performed in Seurat on raw counts using the Louvain approach in PCA space.

RNA velocity and pseudo-time analysis (postnatal and adult in vitro *astrocytes)*

Low-dimension coordinates from t-SNE together with cluster labels annotated by Seurat were used as inputs for RNA velocity inference with the Velocyto and scvelo packages.[101,102] Monocle3[103] was used for pseudo-time and SPRING[42] for graph analyses. Note that in SPRING, the input data were raw counts since SPRING has its own preprocessing framework. Postnatal clusters (Cl1-Cl6) were ordered according to SPRING second dimension (Figure 5D), adult astrocyte clusters (A0-A5) according to diffusion map[104] (DC1 in Figure S5L).

For pathway analysis in Figures 5E, S5M, and S6C HALLMARK_P53_PATHWAY, GO_CYTOSKELETON, GO_RIBONUCLEOPROTEIN_COMPLEX_BIOGENESIS, GO_CELL_CYCLE, LEIN_ASTROCYTE_MARKERS, HALLMARK_PI3K_AKT_MTOR_SIGNALING, HALLMARK_MYC_TARGETS_V1 and HALLMARK_MYC_TARGETS_V2 were used (named ‘‘p53 pathway’’, ‘‘Actin remodelling’’, ‘‘Ribosomal biogenesis’’, ‘‘Cell cycle’’, ‘‘Astrocyte markers’’, ‘‘Pi3K Akt mTOR signalling’’, ‘‘Myc targets v1’’ and ‘‘Myc targets v2’’ in the title of panels, respectively). HALLMARK and GO genesets were downloaded from MsigDB (msigdbr R package V7.5.1).[99,100]

Harmony integration of postnatal, adult and in vivo *astrocytes*

In Figure S5K, postnatal, adult and *in vivo* astrocytes datasets were integrated using the Harmony batch correction algorithm.[46] SCT transform was applied to each dataset independently,[105] then PCA analysis was performed on the combined data. Harmony was applied to the PCA first 20 principal components and a UMAP was obtained based on harmony low dimension space.

ChIP-sequencing and analysis

For ChIP-sequencing experiment, approx $2x10^7$ wild-type astrocytes were incubated for 5 days in basal media supplemented with 25ng/ml BMP4. ChIP was performed using the EZ-Magna ChIP kit (Millipore), according to manufacturer’s instructions, with the following modifications: sonication was performed with Bioruptor Pico with 10-20 cycles (30s on, 30s off at 4°C) until chromatin was average of 100 – 400bp. IP was performed overnight at 4°C with p53-CM5 antibody (Rabbit, Leica, 1:50) or 1mg Normal Rabbit IgG (Santa Cruz). Libraries were prepared with NEBNext Ultra II DNA library prep kit per manufacturer’s instructions with 13 cycles of PCR amplification. Libraries were quantified by Qubit and size distribution assessed by Agilent Bioanalyzer High Sensitivity DNA chip. Indexed libraries were pooled and sequenced on Illumina NextSeq 500 platform with NextSeq 500/550 Mid Output Kit v2.5 (150 Cycles) with Paired End 2x75 bp. Sequencing data was analysed using the nf-core/chipseq Nextflow pipeline.[89] Briefly, QC was performed on Fastq files with FastQC before adapter trimming with TrimGalore. Reads were aligned to the mouse genome GRCm38 and blacklisted regions filtered with BWA. Duplicate reads were removed with Picard, bigwigs generated, and peaks called with MACS2 NarrowPeak setting. Downstream peak analysis was performed in R with the Chippeakanno package[106] and data was visualised with IGV or IgvR R package.

Polysome fractionation

Polysome fractionation was performed as previously described.[107,108] Briefly, approx. $2x10^7$ primary $p53^{flox/flox}$ astrocytes were infected with adenoviral Ad-Null or Ad-iCre virus and incubated for 5 days in basal media supplemented with EGF and FGF, and Torin (250nM) or DMSO control. Cells were lysed in ice cold gradient buffer (15mM Tris-HCl (pH 7.5), 0.3M NaCl, 15mM $MgCl_2$, 0.1mg/ml cycloheximide, 1mg/ml heparin) supplemented with 1% Triton X-100 and 500U/ml recombinant RNase inhibitors. Samples were centrifuged after 2 minutes of lysis, and a small aliquot of supernatant taken as ‘‘Input’’ sample as in Figure S6F, the remainder of the supernatant was layered over a 10-50% sucrose gradient in gradient buffer. Samples were ultracentrifuged at 38000rpm on a SW40Ti rotor (Beckman) for 2 hours at 4°C. Eleven 1ml fractions were collected into 3ml of 7.7M guanidine-HCl and RNA precipitated and purified as in Johannes and Sarnow[108] with additional heparin removal with LiCl. RNA profile was assessed with Agilent

Tapestation High Sensitivity RNA ScreenTape. RNA was reverse transcribed to cDNA using Superscript IV (Invitrogen) according to manufacturer's protocol, before quantitative PCR analysis as described below.

RNA isolation and qRT-PCR

For analysis of astrocyte Trp53 expression in Figure S3A, astrocytes were purified from cortices of 2-3 month, or 1 year, old mice using adult brain dissociation kit followed by ACSA-2 purification as described above for Primary adult astrocyte isolation. RNA was extracted from acutely-purified cell suspension using RNeasy Plus Micro kit according to manufacturer's instructions. RNA was amplified, and cDNA libraries produced using the Smartseq2 method[109] (protocol steps 1-27 with 17 PCR cycles at step 14). Resulting cDNA libraries were diluted to 1ng/μl before qRT-PCR analysis.

For analysis of tissue from the cortical injection site as in Figure S3I, mice were injected stereotaxically as described above. Seven days later, mice were culled by cervical dislocation, and a small area around the injection site was dissected. As a control, a similar size piece was taken from the contralateral cortex. For analysis of young/aged cortical tissue in Figure S3J, 3 month or 1 year old mice were culled by cervical dislocation and a small piece of cortical tissue from the same region as above was taken. For each experiment phenol/chloroform RNA extraction was performed, followed by reverse transcription of RNA to cDNA using iScript gDNA clear cDNA synthesis kit (BioRad) according to manufacturer's instructions. cDNA was diluted at least 1:1 in water before qPCR was performed with qPCRBIO SyGreen Blue Mix Lo-ROX (PCR Biosystems). *Gusb* and *Ppia* were used as housekeeping genes for astrocyte and unpurified tissue lysate studies, respectively. The sequences of primers used in this study are listed in Table S1. Housekeeping-normalised Ct values for EGFR ligand genes (*Areg*, *Btc*, *Egf*, *Epgn*, *Ereg*, *Hbegf*, and *Tgfa*[110]) for the test group (''Ipsi'' and ''aged'' on Figures S3I and S3J respectively) were normalised to the mean value from the reference groups (''contra'' and ''young'' on Figures S3I and S3J respectively). All the genes and replicates were then pooled and displayed as boxplots.

Re-analysis of published RNA-seq datasets

For pathway analysis in Figures S1A and S1B, HALLMARK_P53_PATHWAY was used. HALLMARK genesets were downloaded from MsigDB (msigdbr R package V7.5.1).[99,100] In Figure S1A, bayNorm with setting mean_version=TRUE was applied to single cells from the Zamboni scRNA-seq dataset[1] and normalized counts were plotted. In Figure S1B, RPKM values provided in Li et al.[20] were used. For Figures S3K and S3L (Gyoneva et al.[37] and Guttenplan et al.[36]), read counts for each EGFR ligand gene (*Areg*, *Btc*, *Egf*, *Epgn*, *Ereg*, *Hbegf*, and *Tgfa*[110]) in all conditions were divided by the mean read counts of the reference groups (''Control'' and ''Young'' on Figures S3K and S3L respectively).

Estimating the impact of head injury and additional risk factors on brain cancer outcomes using electronic health records

Electronic health records (EHRs) from secondary care with linkage to patient-level Index of Multiple Deprivation (i.e., an area-based indicator of socioeconomic deprivation) were utilised. Information governance approval was obtained from the Medicines and Healthcare products Regulatory Agency (19222). We considered these three diagnostic categories as exposures: (i) head injury, (ii) phakomatoses (i.e., neurocutaneous syndromes) and (iii) radiation exposure. Diagnoses were recorded in ICD-10 (see Table S2 for code list). To estimate the risk of brain cancer in patients with head injury, phakomatoses or in those who were exposed to radiation, we excluded patients who had a history of brain cancer occurring before the diagnosis of the aforementioned conditions. Controls were identified using propensity score matching, matched by year of birth, sex and socioeconomic deprivation. We performed optimal pair matching using the R matchit package. Cases and matched controls were followed from the date of diagnosis of head injury, phakomatoses or radiation exposure (matched dates were used for controls) until the first record of brain tumour, date of death or date of deregistration, whichever occurred first. For each of the three diagnostic categories, Cox proportional hazard regression models were fitted to estimate the hazard ratios and 95% confidence intervals (CIs) for the risk of brain cancer. To address potential biases, we evaluated the association between head injury and two negative outcome controls (diabetes and fatty liver disease) as there are no plausible mechanisms that head injury would affect the risk of diabetes or fatty liver disease. We evaluated the proportional hazards assumption using the Schoenfeld residuals. Analyses were performed using R (3.6.3) with these packages: data.table, matchit, tidyverse and survival.

QUANTIFICATION AND STATISTICAL ANALYSIS

Statistical analysis was performed using GraphPad Prism 9 or R. All data are expressed as mean±SEM, unless otherwise stated. Significance is stated as follows: p>0.05 (ns), p<0.05 (*), p<0.01 (**), p<0.001 (***), p<0.001 (****). Significance was calculated as indicated in the figure legends. 1- or 2-tailed Student's t test, Wilcoxon test were used for statistical comparisons between two groups. Two-way ANOVA with Tukey's multiple comparisons was used to determine statistical significance of data with two grouping variables. No statistical method was used to predetermine sample size. Sample size was determined based on existing literature and our previous experience.

Current Biology, Volume *33*

Supplemental Information

Injury primes mutation-bearing astrocytes

for dedifferentiation in later life

Holly Simpson Ragdale, Melanie Clements, Wenhao Tang, Elitza Deltcheva, Catia Andreassi, Alvina G. Lai, Wai Hoong Chang, Maria Pandrea, Ivan Andrew, Laurence Game, Imran Uddin, Michael Ellis, Tariq Enver, Antonella Riccio, Samuel Marguerat, and Simona Parrinello

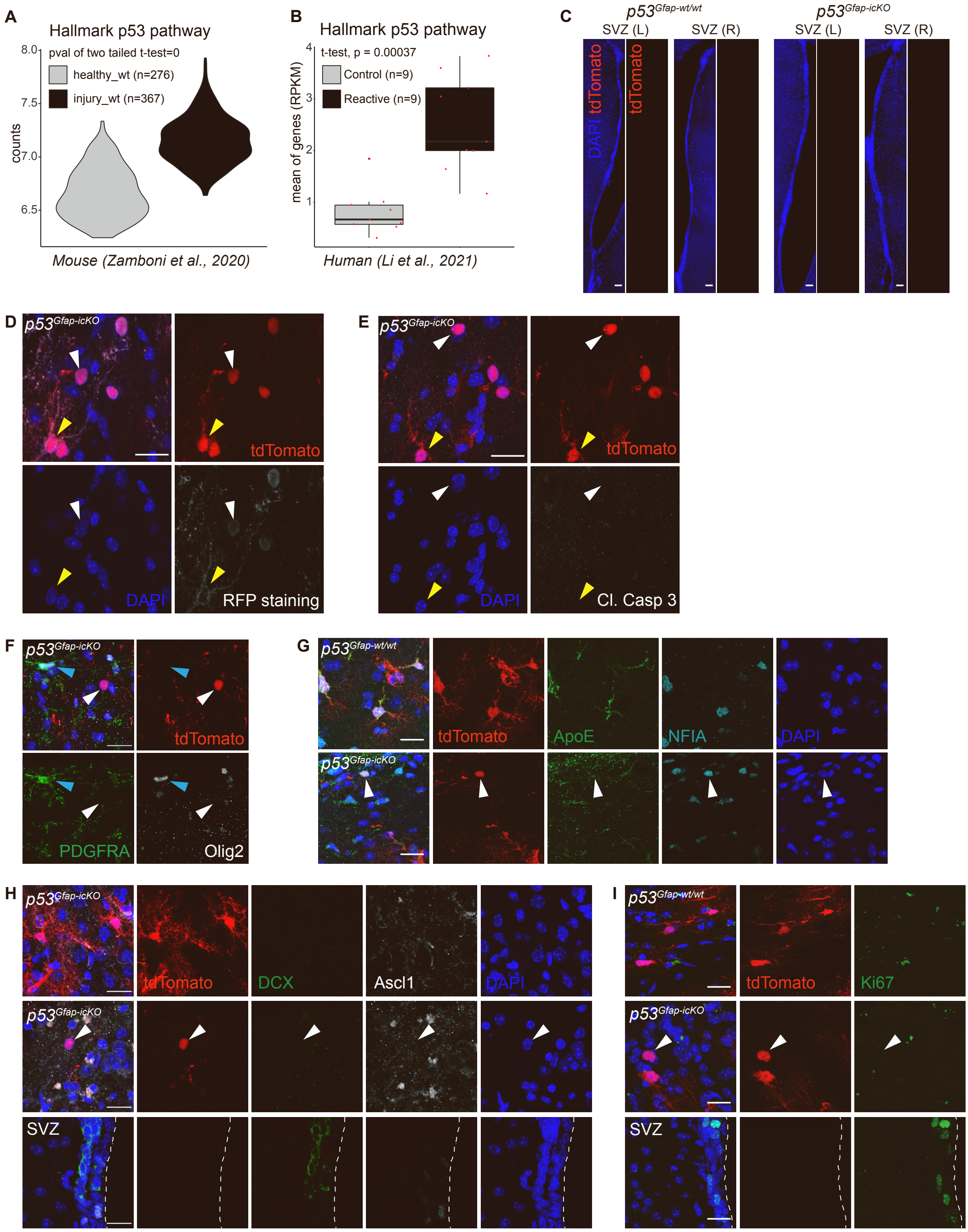

Figure S1. Characterisation of astrocyte-derived tdTomato⁺ cell state, related to Figure 1. A, Analysis of the scRNA-seq dataset from Zamboni et al.[S1] showing a significant increase in p53 pathway gene signatures after injury in mouse cortical astrocytes *in vivo*. **B,** Analysis of RNA-seq dataset from Li et al[S2] shows a significant increase in p53 pathway signatures upon activation in an *in vitro* human astrocyte model of neuroinflammation (TNFα treatment). **C,** Representative images of the subventricular zone (SVZ) from brains presented in Figure 1B, demonstrating that

recombination is localised to endoxifen injection site and not observed in the SVZ. Scale bar=50μm. **D-I,** Representative immunofluorescence images of *p53*$^{Gfap\text{-}wt/wt}$ or *p53*$^{Gfap\text{-}icKO}$ brains 6 weeks after endoxifen intracortical injection stained for the indicated marker proteins. Arrowheads indicate astrocytes (yellow) and astrocyte-derived tdTomato^{+} (AD-tdTomato^{+}, white) cells. Scale bar=20μm. **D,** Red fluorescent protein (RFP) staining indicates process loss in AD-tdTomato^{+} cells is not due to tdTomato fluorophore bleaching. **E,** tdTomato^{+} astrocytes and AD-tdTomato^{+} cells are not positive for cleaved caspase-3. **F,** AD-tdTomato^{+} cells are Olig2^{-}/PDGFRα^{-} and therefore not OPCs. Blue arrowhead indicates a representative Olig2^{+}/PDGFRα^{+} OPC. **G,** Astrocytes and AD-tdTomato^{+} cells are NFIA^{+} but lose ApoE expression. **H,** tdTomato^{+} astrocytes and AD-tdTomato^{+} cells are Ascl1 and doublecortin (DCX) negative. SVZ was included as positive control for staining. **I,** tdTomato^{+} astrocytes and AD-tdTomato^{+} cells are Ki67 negative. SVZ was included as positive control for staining.

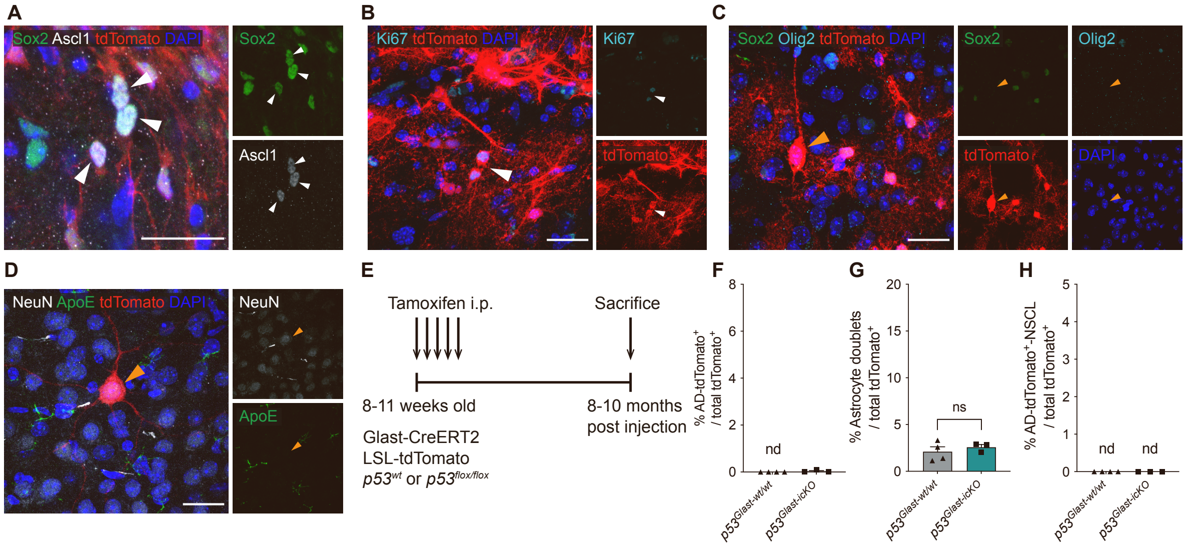

Figure S2. The ageing microenvironment drives dedifferentiation of p53-deficient cortical astrocytes, related to Figure 2. A, Representative image of Ascl1$^+$ AD-tdTomato$^+$ neural stem-like (AD-tdTomato$^+$-NSCL) cells (white arrowhead) in *p53$^{Gfap\text{-}icKO}$* aged cortex 10 months post injection. Scale bar=25µm. **B,** Representative image of Ki67$^+$ cell pair (white arrowhead) in *p53$^{Gfap\text{-}icKO}$* aged cortex. Scale bar=25µm. **C,** Representative image of Sox2$^-$/Olig2$^-$ tdTomato$^+$ astrocyte-derived cell of neuronal morphology (orange arrowhead) in *p53$^{Gfap\text{-}icKO}$* aged cortex. Scale bar=25µm. **D,** Representative image of a tdTomato$^+$ astrocyte-derived cell of neuronal morphology that acquired NeuN expression (orange arrowhead) in *p53$^{Gfap\text{-}icKO}$* aged cortex. Scale bar=25µm. **E,** Schematic of experimental outline for (F-H). 8-11 week old *p53$^{Glast\text{-}wt/wt}$* or *p53$^{Glast\text{-}icKO}$* mice were injected with tamoxifen i.p. for 5 days. 8-10 months after injections, mice were sacrificed and tdTomato$^+$ fate-mapped astrocytes assessed. **F-H,** Quantification of indicated tdTomato$^+$ cell populations in the absence of injury. Mean+SEM, aged *p53$^{Glast\text{-}wt/wt}$* n=4, aged *p53$^{Glast\text{-}icKO}$* n=3, ns not significant. **F** Quantification of AD-tdTomato$^+$ cells, shown as percentage of total tdTomato$^+$ cells. Unpaired two-tailed t-test. **G** Quantification of tdTomato$^+$ astrocyte doublets, shown as percentage of total tdTomato$^+$ cells. Unpaired two-tailed t-test. Note, scale of graph has been matched for comparison to Figure 2D. **H** Quantification of Sox2$^+$/Olig2$^+$ AD-tdTomato$^+$-NSCL cells, shown as percentage of total tdTomato$^+$ cells.

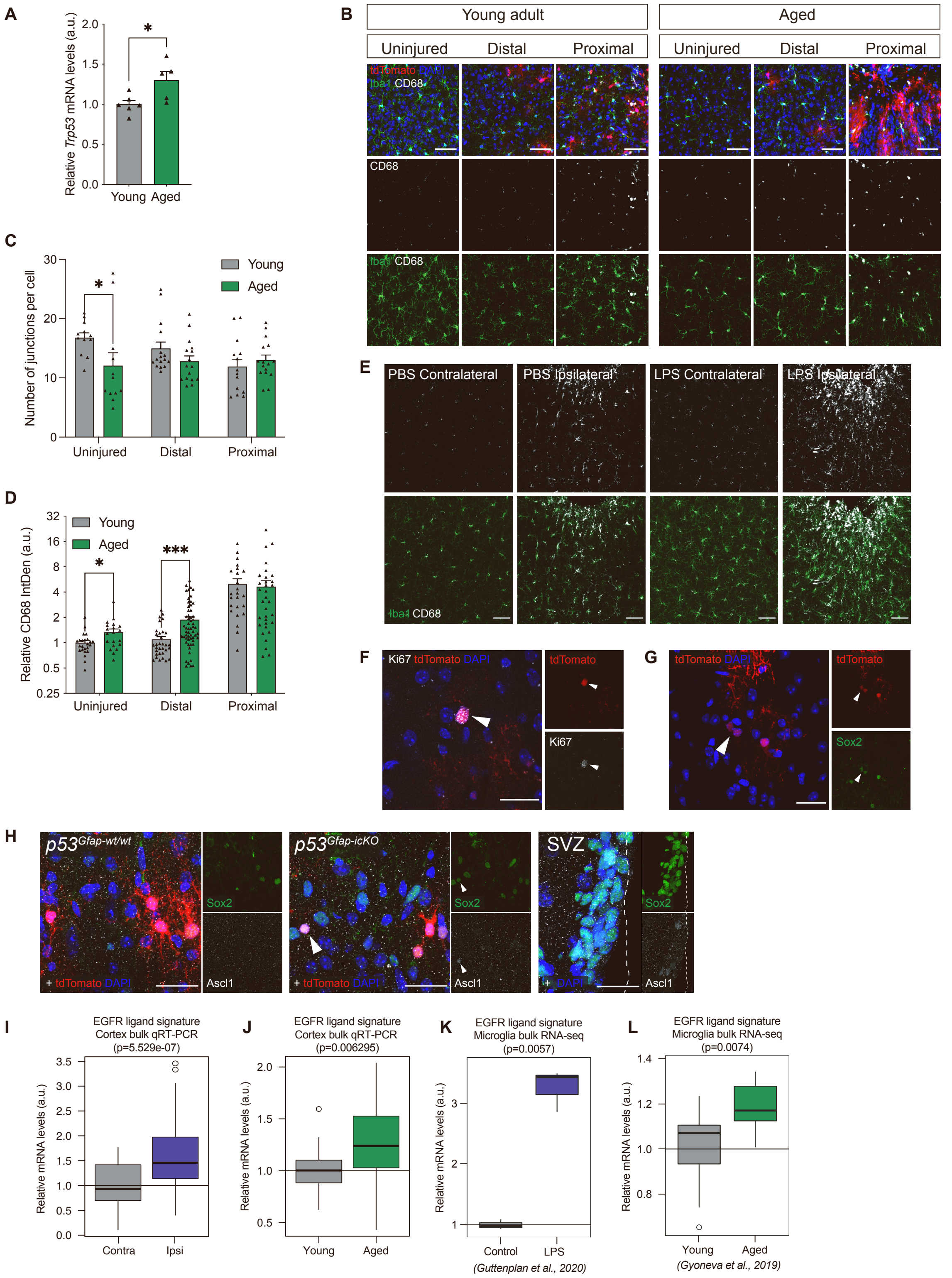

Figure S3. Increased neuroinflammation underlies age-dependent dedifferentiation of p53-deficient astrocytes following early life injury, related to Figure 3. A, qRT-PCR analysis of *Trp53* mRNA levels in astrocytes acutely purified from young (2-3 month old) or aged (1 year old) mouse cortex. Mean±SEM, n=6 young, n=5 aged, *p<0.05, unpaired two-tailed t test. **B,** Representative images of microglia in intact cortices, or in the region distal or proximal to the injection site of

young adults or aged mice. Tissue was immunostained for Iba1 and CD68 to analyse microglia morphology and activation, respectively. Scale bar=50μm. **C,** Skeleton analysis Iba1 staining of microglia in experiments from B. n=3 wildtype animals per condition. Each point represents one field of view. Mean±SEM, a.u. arbitrary units, *p<0.05, Multiple unpaired t tests with Holm-Šídák's multiple comparisons test. **D,** Quantification of relative CD68 intensity IntDen (mean intensity multiplied by particle area) in experiments from B. n=3 wildtype animals per condition. Each point represents one field of view. Mean±SEM, a.u. arbitrary units, ns not significant, *p<0.05, ****p<0.0001, Multiple unpaired t tests with Holm-Šídák's multiple comparisons test. **E,** Representative images of microglial activation in LPS or PBS (vehicle control) injected mice 3 days after i.p. injection as described in Figure 3A, confirming efficacy of LPS treatment. Scale bar=50μm. **F,** Representative image of a $Ki67^+$ AD-$tdTomato^+$ cell (white arrowhead) in $p53^{Gfap\text{-}icKO}$ treated with LPS. Scale bar=25μm. **G,** Representative image of an AD-$tdTomato^+$-NSCL cell doublet (white arrowhead) in $p53^{Gfap\text{-}icKO}$ treated with LPS. Scale bar=25μm. **H,** Representative image of an $Ascl1^+$ AD-$tdTomato^+$-NSCL cell (white arrowhead) in $p53^{Gfap\text{-}icKO}$ treated with LPS. SVZ was included as positive control for Ascl1 staining. Scale bar=25μm. **I,** qRT-PCR analysis of EGFR ligand signature expression in tissue dissected from injury site (ipsilateral) or contralateral area 1 week after intracortical injection. n=5 animals. Wilcoxon one-sided paired test. **J,** qRT-PCR analysis of EGFR ligand signature expression in cortical tissue dissected from young (2-3 months old) or aged (12 months old) mice. n=3 animals. Wilcoxon one-sided unpaired test. **K,** Analysis of EGFR ligand signature expression from RNA-seq of primary microglia treated with LPS[S3]. Two-sided unpaired t-test. **L,** Analysis of EGFR ligand signature expression from RNA-seq of microglia isolated from wildtype young/aged mice[S4]. Two-sided unpaired t-test.

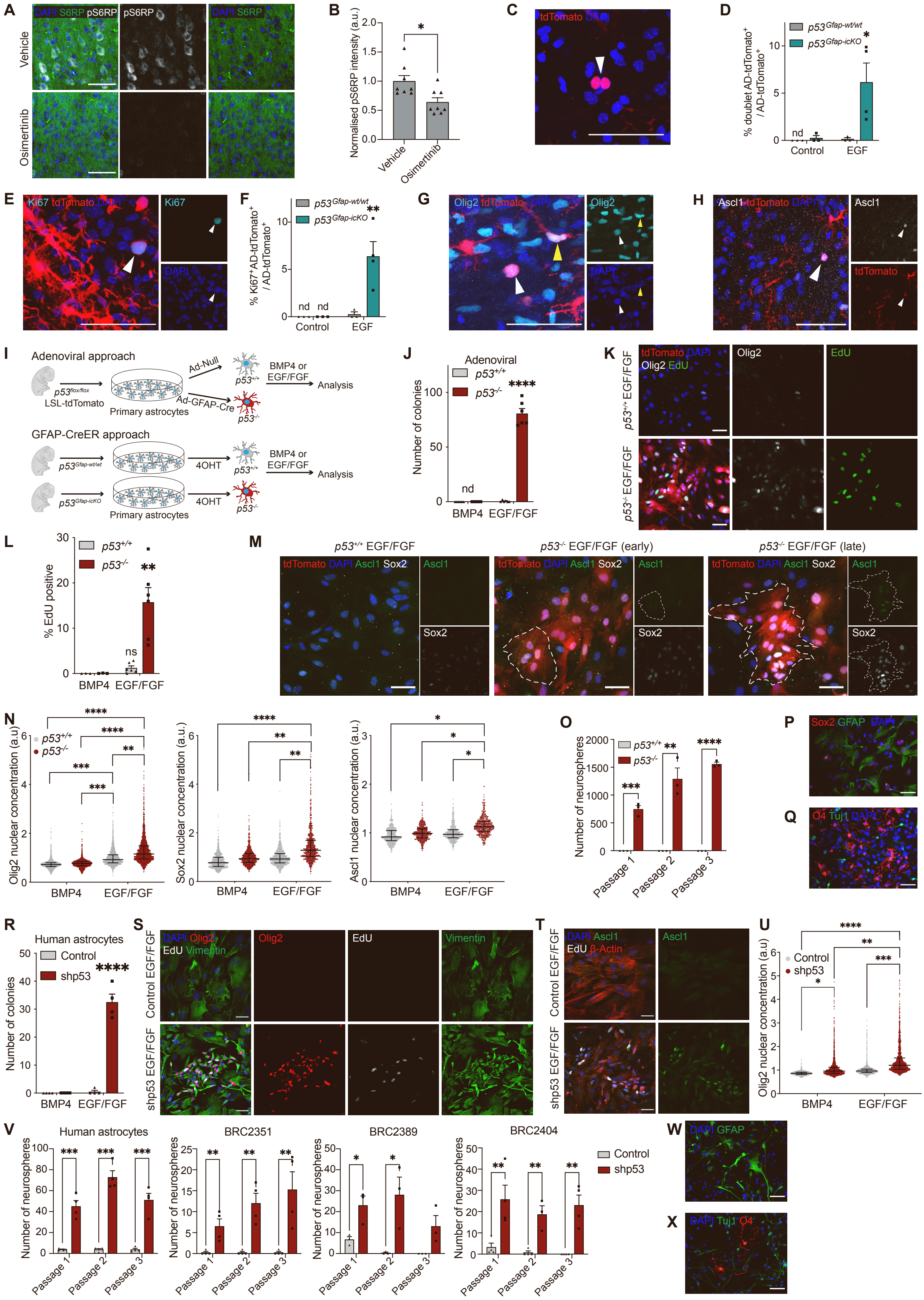

A
Vehicle
Osimertinib
DAPI S6RP pS6RP
pS6RP
DAPI S6RP
B
Normalised pS6RP intensity (a.u.)
Vehicle
Osimertinib
C
tdTomato DAPI
D
p53Gfap-wt/wt
p53Gfap-icKO
% doublet AD-tdTomato+ / AD-tdTomato+
nd
Control
EGF
E
Ki67 tdTomato DAPI
Ki67
DAPI
F
p53Gfap-wt/wt
p53Gfap-icKO
% Ki67+ AD-tdTomato+ / AD-tdTomato+
nd nd
Control
EGF
G
Olig2 tdTomato DAPI
Olig2
DAPI
H
Ascl1 tdTomato DAPI
Ascl1
tdTomato
I
Adenoviral approach
p53flox/flox
LSL-tdTomato
Primary astrocytes
Ad-Null
Ad-GFAP-Cre
p53+/+
p53-/-
BMP4 or EGF/FGF
Analysis
GFAP-CreER approach
p53Gfap-wt/wt
p53Gfap-icKO
4OHT
J
Adenoviral
p53+/+
p53-/-
Number of colonies
nd
BMP4
EGF/FGF
K
p53+/+ EGF/FGF
p53-/- EGF/FGF
tdTomato DAPI Olig2 EdU
Olig2
EdU
L
p53+/+
p53-/-
% EdU positive
ns
BMP4
EGF/FGF
M
p53+/+ EGF/FGF
p53-/- EGF/FGF (early)
p53-/- EGF/FGF (late)
tdTomato DAPI Ascl1 Sox2
Ascl1
Sox2
N
p53+/+
p53-/-
Olig2 nuclear concentration (a.u)
Sox2 nuclear concentration (a.u.)
Ascl1 nuclear concentration (a.u.)
BMP4
EGF/FGF
O
p53+/+
p53-/-
Number of neurospheres
Passage 1
Passage 2
Passage 3
P
Sox2 GFAP DAPI
Q
O4 Tuj1 DAPI
R
Human astrocytes
Control
shp53
Number of colonies
BMP4
EGF/FGF
S
Control EGF/FGF
shp53 EGF/FGF
DAPI Olig2 EdU Vimentin
Olig2
EdU
Vimentin
T
Control EGF/FGF
shp53 EGF/FGF
DAPI Ascl1 EdU β-Actin
Ascl1
U
Control
shp53
Olig2 nuclear concentration (a.u)
BMP4
EGF/FGF
V
Human astrocytes
BRC2351
BRC2389
BRC2404
Number of neurospheres
Passage 1
Passage 2
Passage 3
Control
shp53
W
DAPI GFAP
X
DAPI Tuj1 O4

Figure S4. Dedifferentiation of p53-deficient astrocytes is EGFR-dependent, related to Figure 4. A, Validation of osimertinib efficacy in suppressing EGFR signalling in experiment shown in Figure 4A-C by immunostaining for phospho-S6 ribosomal protein (pS6RP) and total S6RP. **B,** Quantification of pS6RP staining intensity in the cytoplasmic area of injection site shown in A, normalised to total S6RP. n=3 mice per condition. Mean±SEM, *p<0.05, unpaired two-tailed t-test. **C,** Representative image of an AD-tdTomato$^+$-NSCL cell doublet (white arrowhead) in *p53$^{Gfap\text{-}icKO}$* EGF-infused cortex. **D,** Quantification of AD-tdTomato$^+$ cells occurring in doublets, shown as the percentage of total AD-tdTomato$^+$ cells in EGF infusion experiment. n=3 per condition except *p53$^{Gfap\text{-}icKO}$* EGF n=4. Mean+SEM, nd not detected, *p<0.05, Two-way ANOVA with Tukey's multiple comparisons test. **E,** Representative image of a Ki67$^+$ AD-tdTomato$^+$-NSCL cell (white arrowhead) in *p53$^{Gfap\text{-}icKO}$* EGF-infused cortex. **F,** Quantification of Ki67$^+$ AD-tdTomato$^+$ cells as percentage of total AD-tdTomato$^+$ cells. n=3 per condition except *p53$^{Gfap\text{-}icKO}$* EGF n=4. Mean+SEM, nd not detected, **p<0.01, Two-way ANOVA with Tukey's multiple comparisons test. **G,** Representative image of Olig2$^+$ astrocytic (yellow arrowhead) and Olig2$^+$ AD-tdTomato$^+$-NSCL (white arrowhead) in *p53$^{Gfap\text{-}icKO}$* EGF cortex. **H**, Representative image of a Ascl1$^+$ AD-tdTomato$^+$-NSCL cell (white arrowhead) in *p53$^{Gfap\text{-}icKO}$* EGF-infused cortex. **I,** Schematic of experimental outline. Primary cortical astrocytes were isolated from postnatal day 3 (P3) *p53$^{flox/flox}$*; LSL-tdTomato mice or GFAP-CreERT2; *p53$^{flox/flox}$* or *p53$^{wt/wt}$;* LSL-tdTomato mice. p53 recombination and tdTomato labelling was induced via adenoviral GFAP-Cre (or Null adenovirus for control; Figure S4J-Q, Figure S5F,G) or 4OHT treatment (Figure 5 and Figure S5 A-E, H-J), respectively. Cells were incubated in media supplemented with BMP4 or EGF/FGF for 7d before immunofluorescence or clonal analysis. **J,** Quantification of number of colonies visualised by crystal violet staining in indicated culture conditions following Adenovirus-induced recombination. Mean±SEM n=7, nd not detected, ****p<0.0001, Two-way ANOVA with Tukey's multiple comparisons test. **K,** Representative immunofluorescence images of EdU incorporation assays of *p53$^{+/+}$* and *p53$^{-/-}$* astrocytes cultured in EGF/FGF, indicating that colonies of tdTomato$^+$ cells forming in *p53$^{-/-}$* cultures are proliferating (EdU$^+$) and Olig2$^+$. **L,** Quantification of percentage of EdU$^+$ cells in each condition shown in C. Mean±SEM, n=6 independent experiments. ns not significant, **p<0.01, Two-way ANOVA with Tukey's multiple comparisons test. **M,** Representative immunofluorescence images of *p53$^{+/+}$* and *p53$^{-/-}$* astrocytes in EGF/FGF and stained for Ascl1 and Sox2, indicating that colonies of tdTomato$^+$ cells forming in *p53$^{-/-}$* cultures are positive for both neurodevelopmental transcription factors. **N,** Quantification of Olig2, Sox2 and Ascl1 nuclear protein staining intensity, as indicated. Each point represents an individual cell. Line represents median with interquartile range, n=3 independent experiments. *p<0.05, **p<0.01, ***p<0.001, ****p<0.0001, Two-way ANOVA with Tukey's multiple comparisons test on average intensity per condition per replicate. **O,** Quantification of number of neurospheres formed from *p53$^{+/+}$* and *p53$^{-/-}$* astrocytes cultured in EGF/FGF for 7 days and replated in suspension. *p53$^{-/-}$* dedifferentiated astrocytes selectively form neurospheres which can be serially passaged for at least 3 passages. *p53$^{+/+}$*

astrocytes do not form neurospheres. Mean±SEM, **p<0.01, ***p<0.001, ****p<0.0001, Multiple t-tests with Two stage linear step-up procedure of Benjamin, Krieger and Yekutieli. **P-Q,** Representative immunofluorescence images of dedifferentiated $p53^{-/-}$ astrocytes induced to re-differentiate by mitogen removal and stained for astrocyte markers (P, $GFAP^{+}$, $Sox2^{low}$), or oligodendrocytes (Q, $O4^{+}$) and neuroblasts (Q, $Tuj1^{+}$). **R,** Quantification of number of colonies visualised by crystal violet staining in indicated culture conditions following lentiviral mediated p53 downregulation (shp53) in primary human astrocytes. Mean±SEM n=4, ****p<0.0001, Two-way ANOVA with Tukey's multiple comparisons test. **S-T,** Representative immunofluorescence images of Olig2 (**S**), Ascl1 (**T**) immunostaining and EdU incorporation of wildtype of shp53 human astrocytes in EGF/FGF, indicating that colonies of dedifferentiated cells forming in shp53 cultures are positive for both neurodevelopmental transcription factors and proliferative. Vimentin (S) and β-actin (T) were used to label cell morphology. **U,** Quantification of Olig2 nuclear protein staining intensity. Each point represents an individual cell. Line represents median with interquartile range, n=3 independent experiments. *p<0.05, **p<0.01, ***p<0.001, ****p<0.0001, Two-way ANOVA with Tukey's multiple comparisons test on average intensity per condition per replicate. **V,** Quantification of number of neurospheres formed from control or shp53 human astrocytes (primary or fetal neural stem cell-derived (BRC2351, BRC2389, BRC2404), as indicated) cultured in EGF/FGF for 7 days and replated in suspension. shp53 dedifferentiated astrocytes selectively form neurospheres which can be serially passaged for at least 3 passages. p53 wildtype control astrocytes do not form neurospheres. Mean±SEM n=4 per condition except BRC2389 n=3. *p<0.05, **p<0.01, ***p<0.001, Multiple t-tests with Two stage linear step-up procedure of Benjamin, Krieger and Yekutieli. **W-X,** Representative immunofluorescence images of shp53-dedifferentiated human astrocytes induced to re-differentiate by mitogen removal and stained for astrocyte markers (W, $GFAP^{+}$), or oligodendrocytes (X, $O4^{+}$) and neuroblasts (X, $Tuj1^{+}$) markers. All images, scale bar=50μm.

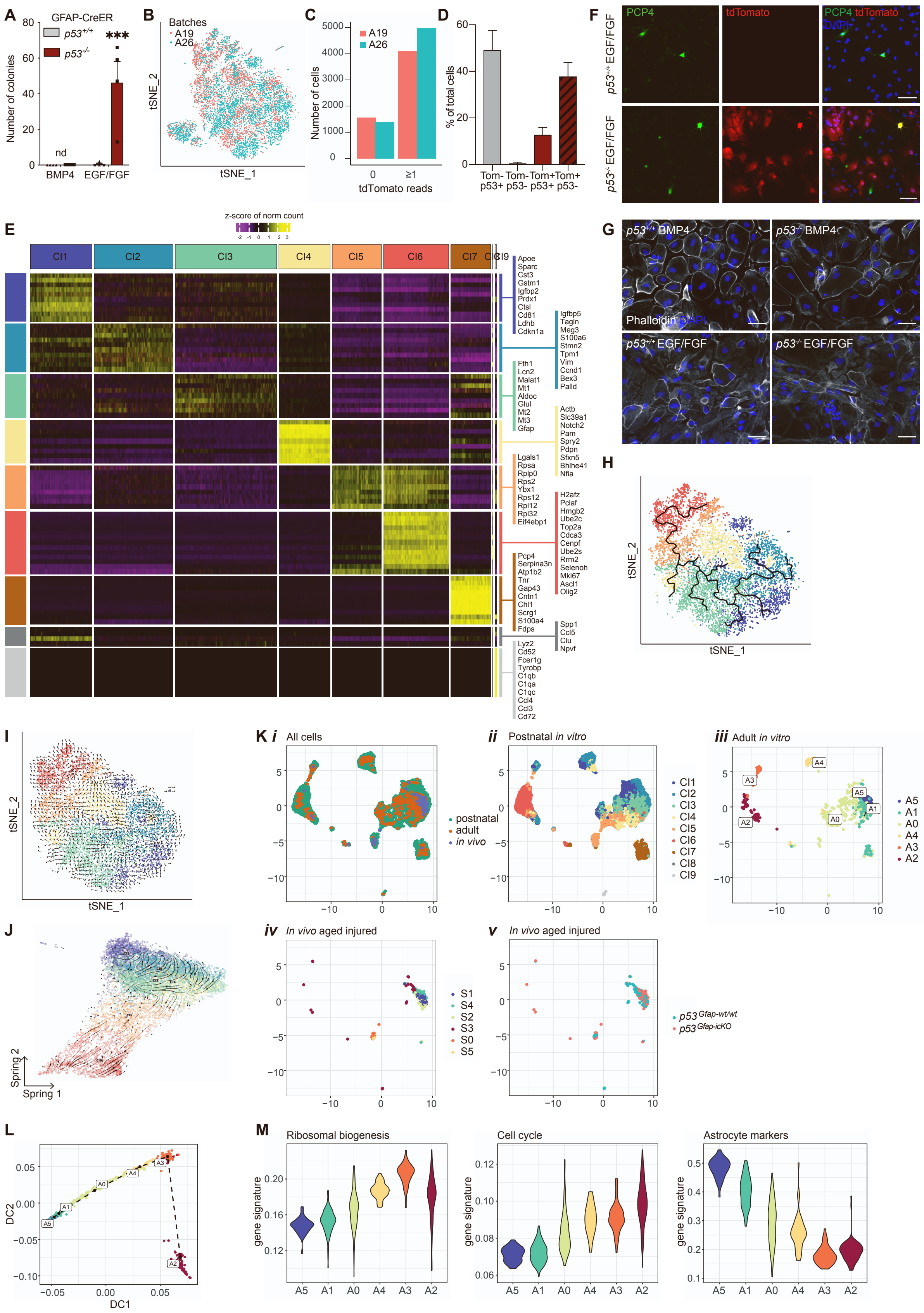

A
GFAP-CreER
p53+/+
p53-/-
***
nd
Number of colonies
BMP4
EGF/FGF
B
Batches
A19
A26
tSNE_2
tSNE_1
C
A19
A26
Number of cells
0
≥1
tdTomato reads
D
% of total cells
Tom- p53+
Tom- p53-
Tom+ p53+
Tom+ p53-
F
PCP4
tdTomato
PCP4 tdTomato DAPI
p53+/+ EGF/FGF
p53-/- EGF/FGF
E
z-score of norm count
Cl1
Cl2
Cl3
Cl4
Cl5
Cl6
Cl7
Cl8
Cl9
Apoe
Sparc
Cst3
Gstm1
Igfbp2
Prdx1
Ctsl
Cd81
Ldhb
Cdkn1a
Igfbp5
Tagln
Meg3
S100a6
Stmn2
Tpm1
Vim
Ccnd1
Bex3
Palld
Fth1
Lcn2
Malat1
Mt1
Aldoc
Glul
Mt2
Mt3
Gfap
Actb
Slc39a1
Notch2
Pam
Spry2
Pdpn
Sfxn5
Bhlhe41
Nfia
Lgals1
Rpsa
Rplp0
Rps2
Ybx1
Rps12
Rpl12
Rpl32
Eif4ebp1
H2afz
Pclaf
Hmgb2
Ube2c
Top2a
Cdca3
Cenpf
Ube2s
Rrm2
Selenoh
Mki67
Ascl1
Olig2
Pcp4
Serpina3n
Atp1b2
Tnr
Gap43
Cntn1
Chl1
Scrg1
S100a4
Fdps
Spp1
Ccl5
Clu
Npvf
Lyz2
Cd52
Fcer1g
Tyrobp
C1qb
C1qa
C1qc
Ccl4
Ccl3
Cd72
G
p53+/+ BMP4
p53-/- BMP4
Phalloidin DAPI
p53+/+ EGF/FGF
p53-/- EGF/FGF
H
tSNE_2
tSNE_1
I
tSNE_2
tSNE_1
J
Spring 2
Spring 1
K i All cells
postnatal
adult
in vivo
ii Postnatal in vitro
iii Adult in vitro
A5
A1
A0
A4
A3
A2
iv In vivo aged injured
S1
S4
S2
S3
S0
S5
v In vivo aged injured
p53Gfap-wt/wt
p53Gfap-icKO
L
DC2
DC1
M
Ribosomal biogenesis
Cell cycle
Astrocyte markers
gene signature

Figure S5. scRNA-seq of dedifferentiating *p53*$^{-/-}$ astrocytes *in vitro*, related to Figure 5. A, Quantification of number of colonies visualised by crystal violet staining in indicated culture conditions following 4OHT treatment. Mean±SEM, n=4 independent experiments. nd not detected, ***p<0.001, Two-way ANOVA with Tukey's multiple comparisons test. **B,** t-SNE as in Figure 5B, coloured by replicate (A19 and A26). **C,** Number of cells per replicate with tdTomato reads of 0 or ≥1, (see filtering steps in methods). **D,** Quantification of immunostaining for p53 at 5d post EGF/FGF + 4OHT treatment. Some tdTomato^{+} astrocytes retain p53 expression. Mean±SEM, n=2 independent replicates. **E,** Heatmap of z-score normalized gene expression for the 9 scRNA-seq clusters. **F**, Representative image of PCP4 immunostaining in *p53*$^{+/+}$ and *p53*$^{-/-}$ astrocyte cultures in EGF/FGF. PCP4^{+} cells are present in all conditions, indicating that these cells represent a contaminating cell population within the preparations. Scale bar=50μm. **G**, Representative image of phalloidin staining in *p53*$^{+/+}$ and *p53*$^{-/-}$ astrocyte cultures in BMP4 or EGF/FGF. Note that actin remodelling occurs in both genotypes upon mitogen exposure. Scale bar=50μm. **H,** Monocle pseudotime trajectory of tdTomato+ cells in tSNE. **I,** velocyto[S5] RNA velocity analysis overlayed onto t-SNE. **J,** scvelo pseudotime analysis of tdTomato+ cells in SPRING[S6]. **K,** Harmony analysis of integrated scRNAseq datasets (*i*) from: postnatal *in vitro* astrocytes (*ii*), adult *in vitro* astrocytes (*iii*) and *in vivo* tdTomato^{+} cells acutely FACS-purified from the cortices of aged and injured *p53*$^{Gfap\text{-}wt/wt}$ *and p53*$^{Gfap\text{-}icKO}$ mice (*iv*-*v*). Colouring is based on dataset (*i*), clusters (*ii-iv*) and genotypes (*v*). Note that dedifferentiated cells were only found in *p53*$^{Gfap\text{-}icKO}$, but not in *p53*$^{Gfap\text{-}wt/wt}$ (i*v, v*) brains. **L,** Diffusion map of adult *in vitro* astrocyte data coloured by clusters[S7]. **M,** Violin plots of pathway analysis in ordered clusters of adult *in vitro* astrocyte.

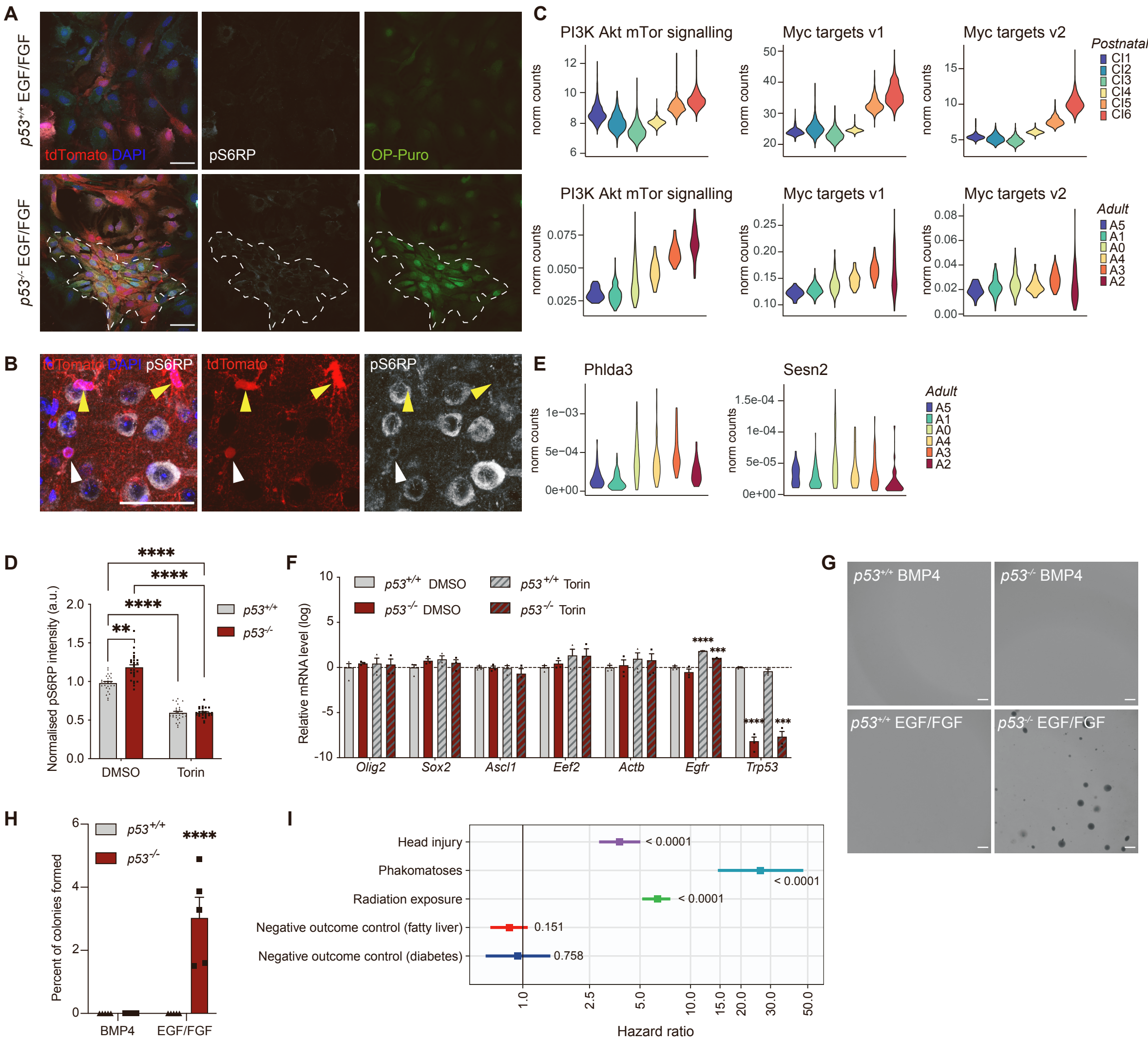

Figure S6. mTOR activity is increased upon p53 loss, related to Figure 6. A, Representative image of *p53*$^{+/+}$ and *p53*$^{-/-}$ astrocyte cultures in EGF/FGF media stained for OP-puro and pS6RP. mTOR activity correlates with increased protein synthesis, as assessed by OP-Puro incorporation. Scale bar=50μm. **B,** Representative image of pS6RP immunostaining in *p53*$^{Gfap\text{-}icKO}$ aged injured mouse. pS6RP is higher in AD-tdTomato^{+}-NSCL cells (white arrowheads) compared to astrocytic tdTomato^{+} cells (yellow arrowhead). Note that the highest expression of pS6RP is observed in tdTomato^{-} neurons. Scale bar=50μm. **C,** Violin plots of indicated genes in ordered clusters of postnatal (top) and adult (bottom) *in vitro* scRNAseq datasets. **D,** Quantification of pS6RP intensity in *p53*$^{+/+}$ or *p53*$^{-/-}$ astrocytes cultured in EGF/FGF in the presence of Torin or DMSO vehicle control, normalised to total S6RP. Torin treatment significantly reduced pS6RP intensity. Pixel intensity of cytoplasmic area of whole field of view was quantified for n=3 independent experiments. Each point indicates a field of view. Mean±SEM, **p<0.01, ****p<0.0001, Two-way ANOVA with Tukey's multiple comparisons test. **E,** Violin plots of indicated genes in the ordered clusters from adult *in vitro* scRNA-seq data. **F,** qRT-PCR of total mRNA of input of polysome profiling experiment in Figure 6G. Changes in polysome profile are not due to changes in mRNA levels of genes. ***p<0.001, ****p<0.0001, Two-way ANOVA with

Tukey’s multiple comparisons test. **G-H,** Soft agar assay of in vitro astrocytes. $p53^{-/-}$ astrocytes cultured in EGF/FGF conditions showed anchorage-independence after serial passaging, while other conditions did not. Scale bar=1mm. Mean±SEM, n=5, ****$p<0.0001$, Two-way ANOVA with Tukey’s multiple comparisons test. **I,** Matched cohort analysis to estimate the risk of developing glioma in people with a history of brain injury. Phakomatoses and exposure to radiation were used as positive controls as they are both known risk factors for brain cancer. Diabetes and fatty liver disease were used as negative outcome controls, showing no increased risk associated with brain injury. Hazard ratio and 95% confidence interval are shown.

Gene	Forward primer (5'-3')	Reverse primer (5'-3')	Reference (if applicable)
Actb	TCGTTGCCGGTCCACACCCG	CTCCTCAGGGGCCACACGCAG	S8
Areg	GCCATCATCCTCGCAGCTA	ATGATTCAACTTTTACCCTGCATTG	This study
Ascl1	ATGCAGCTACTGTCCAAACG	AACAGTAAGGGGTGGGTGTG	S9
Btc	AAACCCACTTCTCTCGGTGC	AAACAGGTCCACTCGCTCAC	This study
Eef2	GAGAATCCGTCGCCATCCGCCA	CGGGCTTGATGCGTTCAGCGA	S8
Egf	TCGAGAGAAGCGAGAGAAGC	TGTTCCATCTGGGTCAATCCG	This study
Egfr	TCTGCCACCTATGCCACGCCA	CCACTGCCATTGAACGTACCCAG	This study
Epgn	CATTTAACAACACCGAAGCTGACT	GCTCACATCGTTGTCCCGT	This study
Ereg	GACGCTGCTTTGTCTAGGTTCC	CACACGGGGATCGTCTTCC	This study
Gusb	AACAACACACTGACCCCTCA	ACCACAGATCGATGCAGTCC	This study
Hbegf	GTCGTCCGTCTGTCTTCTTGT	CTAGCCACGCCCAACTTCAC	This study
Olig2	CGAGCACCTCAAATCTAATTCA	GGACGATGGGCGACTAGA	S9
Ppia	GCGTCTCCTTCGAGCTGTT	AAAGTCACCACCCTGGCA	S10
Sox2	TCCAAAAACTAATCACAACAATCG	GAAGTGCAATTGGGATGAAAA	This study
Tgfa	CATTATCACCTGTGTGCTGATCC	CCTTTTCTTGGTTGGGCTGTCATA	This study
Trp53	GGACGGGACAGCTTTGAGGT	GTGGGCAGCGCTCTCTTTG	This study

Table S1. List of primer sequences used in this study, related to STAR methods.

ICD-10	Terms	Diagnostic category
S06	Intracranial injury	Head injury
S060	Concussion	Head injury
S061	Traumatic cerebral oedema	Head injury
S062	Diffuse brain injury	Head injury
S063	Focal brain injury	Head injury
S064	Epidural haemorrhage	Head injury
S065	Traumatic subdural haemorrhage	Head injury
S066	Traumatic subarachnoid haemorrhage	Head injury
S067	Intracranial injury with prolonged coma	Head injury
S068	Other intracranial injuries	Head injury
S069	Intracranial injury, unspecified	Head injury
Q850	Neurofibromatosis (nonmalignant)	Phakomatoses
Q851	Tuberous sclerosis	Phakomatoses
Q858	Other phakomatoses, not elsewhere classified	Phakomatoses
Q859	Phakomatosis, unspecified	Phakomatoses
W88	Exposure to ionizing radiation	Radiation
W91	Exposure to unspecified type of radiation	Radiation
X39	Exposure to other and unspecified forces of nature	Radiation
Y365	War operations involving nuclear weapons	Radiation
Z58	Problems related to physical environment	Radiation
Z584	Exposure to radiation	Radiation

Z923	Personal history of irradiation	Radiation
E10	Insulin-dependent diabetes mellitus	Diabetes
E11	Non-insulin-dependent diabetes mellitus	Diabetes
E12	Malnutrition-related diabetes mellitus	Diabetes
E13	Other specified diabetes mellitus	Diabetes
E14	Unspecified diabetes mellitus	Diabetes
G590	Diabetic mononeuropathy	Diabetes
G632	Diabetic polyneuropathy	Diabetes
H280	Diabetic cataract	Diabetes
H360	Diabetic retinopathy	Diabetes
M142	Diabetic arthropathy	Diabetes
N083	Glomerular disorders in diabetes mellitus	Diabetes
O240	Diabetes mellitus in pregnancy: Pre-existing diabetes mellitus, insulin-dependent	Diabetes
O241	Diabetes mellitus in pregnancy: Pre-existing diabetes mellitus, non-insulin-dependent	Diabetes
O242	Diabetes mellitus in pregnancy: Pre-existing malnutrition-related diabetes mellitus	Diabetes
O243	Diabetes mellitus in pregnancy: Pre-existing diabetes mellitus, unspecified	Diabetes
K700	Alcoholic fatty liver	Fatty liver
K758	Other specified inflammatory liver diseases	Fatty liver
K760	Fatty (change of) liver, not elsewhere classified	Fatty liver
C71	Malignant neoplasm of brain	Brain cancer
C710	Cerebrum, except lobes and ventricles	Brain cancer
C711	Frontal lobe	Brain cancer
C712	Temporal lobe	Brain cancer
C713	Parietal lobe	Brain cancer
C714	Occipital lobe	Brain cancer
C715	Cerebral ventricle	Brain cancer
C716	Cerebellum	Brain cancer
C717	Brain stem	Brain cancer
C718	Overlapping lesion of brain	Brain cancer
C719	Brain, unspecified	Brain cancer
C72	Malignant neoplasm of spinal cord, cranial nerves and other parts of central nervous system	Brain cancer
C728	Overlapping lesion of brain and other parts of central nervous system	Brain cancer
C729	Central nervous system, unspecified	Brain cancer

Table S2. Code list for conditions included in the matched cohort analysis, related to STAR methods.

Supplementary References

S1. Zamboni, M., Llorens-Bobadilla, E., Magnusson, J. P., and Frisen, J. (2020). A Widespread Neurogenic Potential of Neocortical Astrocytes Is Induced by Injury. Cell Stem Cell *27*, 605-617. 10.1016/j.stem.2020.07.006.

S2. Li, J., Pan, L., Pembroke, W. G., Rexach, J. E., Godoy, M. I., Condro, M. C., Alvarado, A. G., Harteni, M., Chen, Y.-W., Stiles, L., et al. (2021). Conservation and divergence of vulnerability and responses to stressors between human and mouse astrocytes. Nature Communications *12*, 3958. 10.1038/s41467-021-24232-3.

S3. Guttenplan, K. A., Weigel, M. K., Adler, D. I., Couthouis, J., Liddelow, S. A., Gitler, A. D., and Barres, B. A. (2020). Knockout of reactive astrocyte activating factors slows disease progression in an ALS mouse model. Nature Communications *11*. 10.1038/s41467-020-17514-9.

S4. Gyoneva, S., Hosur, R., Gosselin, D., Zhang, B., Ouyang, Z., Cotleur, A. C., Peterson, M., Allaire, N., Challa, R., Cullen, P., et al. (2019). Cx3cr1-deficient microglia exhibit a premature aging transcriptome. Life Science Alliance *2*, e201900453. 10.26508/lsa.201900453.

S5. Bergen, V., Lange, M., Peidli, S., Wolf, F. A., and Theis, F. J. (2020). Generalizing RNA velocity to transient cell states through dynamical modeling. Nature Biotechnology *38*, 1408-1414. 10.1038/s41587-020-0591-3.

S6. La Manno, G., Soldatov, R., Zeisel, A., Braun, E., Hochgerner, H., Petukhov, V., Lidschreiber, K., Kastriti, M. E., Lönnerberg, P., Furlan, A., et al. (2018). RNA velocity of single cells. Nature *560*, 494-498. 10.1038/s41586-018-0414-6.

S7. Angerer, P., Haghverdi, L., Büttner, M., Theis, F. J., Marr, C., and Buettner, F. (2016). destiny: diffusion maps for large-scale single-cell data in R. Bioinformatics *32*, 1241-1243.

S8. Thoreen, C. C., Chantranupong, L., Keys, H. R., Wang, T., Gray, N. S., and Sabatini, D. M. (2012). A unifying model for mTORC1-mediated regulation of mRNA translation. Nature *485*, 109-113. 10.1038/nature11083.

S9. Ottone, C., Krusche, B., Whitby, A., Clements, M., Quadrato, G., Pitulescu, M. E., Adams, R. H., and Parrinello, S. (2014). Direct cell–cell contact with the vascular niche maintains quiescent neural stem cells. Nature Cell Biology *16*, 1045-1056. 10.1038/ncb3045.

S10. Timaru-Kast, R., Herbig, E. L., Luh, C., Engelhard, K., and Thal, S. C. (2015). Influence of Age on Cerebral Housekeeping Gene Expression for Normalization of Quantitative Polymerase Chain Reaction after Acute Brain Injury in Mice. Journal of Neurotrauma *32*, 1777-1788. 10.1089/neu.2014.3784.
